# 12/15-lipoxygenase orchestrates murine wound healing via PPARγ-activating oxylipins acting holistically to dampen inflammation

**DOI:** 10.1073/pnas.2502640122

**Published:** 2025-09-04

**Authors:** Christopher P. Thomas, Victoria J. Tyrrell, James J. Burston, Sam R. C. Johnson, Maceler Aldrovandi, Jorge Alvarez-Jarreta, Rossa Inglis, Adam Leonard, Lydia Fice, Jeremie Costales, Antonio Vidal-Puig, Majd Protty, Carol Guy, Robert Andrews, Barbara Szomolay, Ben C. Cossins, Ana Cardus Figueras, Stefania Carobbio, Simon A. Jones, Valerie B. O’Donnell

**Affiliations:** ^a^School of Pharmacy and Pharmaceutical Sciences, Cardiff University, Cardiff CF10 3NB, United Kingdom; ^b^Systems Immunity Research Institute and Division of Infection and Immunity, School of Medicine, Cardiff University, Cardiff CF14 4XN, United Kingdom; ^c^Centro de Investigacion Principe Felipe, Spanish Biomedical Research Centre in Diabetes and Associated Metabolic Disorders (CIBERDEM), Valencia 46012, Spain; ^d^University of Cambridge Metabolic Research Laboratories Institute of Metabolic Science, Addenbrookes Hospital, Cambridge CB2 0QQ, United Kingdom

**Keywords:** lipid, wound, oxylipin, lipoxygenase

## Abstract

12/15-lipoxygenase (12/15-LOX, *Alox15*) generates bioactive oxygenated lipids during inflammation, however its homeostatic role(s) in normal healing are unclear. Here, the role of 12/15-LOX in resolving skin wounds was elucidated, focusing on how its lipids act together in physiologically relevant amounts. In mice, wounding caused acute appearance of 12/15-LOX-expressing macrophages and stem cells, coupled to early generation of ~12 monohydroxy-oxylipins and enzymatically oxidized phospholipids (eoxPL). *Alox15* deletion increased collagen deposition, stem cell/fibroblast proliferation, IL6/pSTAT3, pSMAD3, and interferon (IFN)-γ levels. Conversely, CD206 expression, F480+ cells, and MMP9 and MMP2 activities were reduced. *Alox15^−/−^* skin was deficient in PPARγ/adiponectin activity. Furthermore, while pro-inflammatory genes were upregulated as normal during wounding, many including *Il6, Il1b, ccl4, Cd14, Cd274, Clec4d, Clec4e, Csf3, Cxcl2, and miR-21* failed to revert to baseline during healing, indicating disruption of PPARγ’s anti-inflammatory brake on NLRP3/inflammasome and TGF-β signaling. Reconstituting *Alox15^−/−^* wounds with a physiological mixture of *Alox15*-derived primary oxylipins generated by healing wounds restored MMP and dampened collagen deposition. The oxylipin mixture activated the PPARγ response element in vitro, while in vivo, its coactivator, *Helz2*, was significantly upregulated as well as several fatty acid and prostaglandin PPARγ ligands. Additional inflammatory and proliferative gene networks impacted by *Alox15^−/−^* included *Elf4, Cebpb*, and Tcf3. In summary, 12/15-LOX generates abundant monohydroxy oxylipins that act together via PPARγ. The identification of multiple gene alterations reveals several targets for treating nonhealing wounds. Our studies demonstrate that 12/15-LOX oxylipins act in concert, dampening inflammation in vivo, revealing the need to consider lipid signaling holistically.

## Significance

Defective wound healing is a significant global problem. Macrophage 12/15-lipoxygenase (12/15-LOX, *Alox15*) generates abundant lipid mediators termed oxylipins during inflammation. However, its physiological role in resolving wound healing is unclear, with studies so far assessing the bioactivity of individual lipids pharmacologically rather than holistically in physiological amounts. We report that *Alox15* deficiency in mice caused a fibrotic response with failure to dampen inflammation due to a dysregulated peroxisome proliferator activated receptor (PPAR)γ axis, driven by failure to resolve NLRP3/inflammasome and transforming growth factor (TGF)-β signaling. Treatment of *Alox15^−/−^* wounds with physiological mixtures of PPARγ-activating 12/15-LOX monohydroxy products restored the phenotype. Several additional transcriptional networks (*Elf4, Cebpb,* and *Tcf3*) controlled by *Alox15* were uncovered, identifying targets for promoting physiological wound healing.

12/15-lipoxygenase (12/15-LOX) (*Alox15*) is a leukocyte enzyme highly expressed in murine resident peritoneal macrophages. The human homolog, 15-LOX1 (*ALOX15*), is inducible in peripheral monocytes in response to Th2 cytokines and expressed basally in reticulocytes, eosinophils, and airway epithelium (1, 2). *Alox15^−/−^* mice are protected against atherosclerosis, diabetes, hypertension, and abdominal aortic aneurysm and show reduced thrombosis, while conversely, they develop worse arthritis (3–7). This indicates that the pathway is a significant player in inflammatory vascular disease. However, the function of 12/15-LOX in normal healing is less clear.

12/15-LOX generates families of structurally related lipid mediators through oxidation of unsaturated fatty acids (FA) and complex lipids (8–11). The monohydroxy forms of oxidized FAs are first generated by LOXs, with the most abundant being usually derived from arachidonate (AA). These 12/15-LOX derived lipids can independently mediate bioactions relevant to inflammation, such as activation of PPARγ (which dampens cytokines such as IL-6 and TNFα) (7, 12–20). Studies to date have generally focused on their bioactions when added individually, for example (21–24). However, in vivo they are generated in mixtures comprising large numbers of species at varying amounts. This is particularly relevant to PPARγ, which recognizes overall ligand “tone” at relatively low affinity, rather than specific lipid structures at high affinity via GPCRs. The most quantitatively abundant free acid 12/15-LOX products are monohydroxy FAs, from arachidonic acid (AA) and other polyunsaturated fatty acids. Additionally, “specialized pro-resolving mediators” (SPM), such as resolvins, protectins, and maresins, are described as rarer products of the pathway (25). Here, the primary monohydroxy FAs are further metabolized, generating oxygenated di- and tri-hydroxy FAs reported to signal via activation of G protein-coupled receptors (GPCRs) that include ALX/FPR2, DRV1/GPR32, DRV2/GPR18, and ERV1/ChemR23 (26, 27). However, while SPM can dampen inflammation pharmacologically, their endogenous generation and GPCR binding were recently queried (28–34).

Skin wounding (punch biopsy) represents a tractable model of physiological inflammation resolution comprising four phases: hemostasis, inflammation, proliferation, and remodeling. This model provides an ideal framework for testing the impact of *Alox15*. Throughout this process, lymphoid, myeloid, and tissue-resident cells interact, producing signaling molecules that work in an orchestrated manner. During hemostasis, clotting factors and angiogenic factors decrease bleeding and stimulate angiogenesis (35). During the inflammatory phase, neutrophil and macrophage infiltration supports release of chemokines, cytokines, inflammatory agents, and antigen control factors (36). Later, the proliferation phase is characterized by fibroblast and keratinocyte migration from the wound edge, mediating contraction and closure (37). Last, during remodeling, increased deposition and cross-linking of collagen occurs, balanced by removal of excess extracellular matrix by myofibroblast-derived matrix metalloproteinases (MMPs) (38). Herein, we employed genetic, transcriptomic, and lipidomic approaches to investigate the role of *Alox15* and its lipids in physiological skin wound healing. We found that the gene plays a critical role in ensuring that the response is finely tuned to enable effective healing. Without 12/15-LOX, cellular and tissue responses proceed at accelerated rates suggestive of fibrosis. Abundant monohydroxy FAs, many known PPARγ ligands, appear responsible for the phenotype when applied in physiological amounts, acting through PPARγ response element (PPRE)-independent anti-inflammatory activities. Our study highlights a central role for *Alox15* in normal healing, defines several potential targets for promoting healing, and demonstrates the need to consider lipid biology holistically when delineating cellular signaling roles that drive health and disease.

## Methods

### Animal Model.

Mice (8 to 12 wk old C57/B6/J) were purchased from Charles River UK (Margate, UK), while *Alox15^−/−^* mice were bred in-house (F11, C57BL/6J) in isolators. All animal experiments were performed in accordance with the United Kingdom Home Office Animals (Scientific Procedures) Act of 1986, under License (PPL 30/3334). The generation of healing wounds is described in *SI Appendix*, *Supplementary Methods*.

### Generation of Histological Tissue Sections and Staining Protocols.

At various time points up to 14 d, wounds were harvested, processed, and stained either using DAB or fluorescence immunohistochemistry, as described in *SI Appendix*, *Supplementary Methods*. Collagen was stained using Masson Trichrome, and images were acquired and analyzed using microscopy as described in *SI Appendix*, *Supplementary Methods*.

### Ribonucleic acid sequencing (RNA-seq).

Wound tissue dissected from 2 mice (8 wounds in total, 4 wounds per mouse) to generate each sample (n = 4/condition) were snap-frozen in liquid N_2_ before being stored at −80 °C. RNA was isolated using the RNeasy MinElute Cleanup Kit (Catalogue number 74204 Qiagen, MD), as described in *SI Appendix*,*Supplementary Methods*. Total RNA was depleted of ribosomal RNA, and sequencing libraries were prepared with the Illumina®TruSeq Stranded Total RNA Library Prep Gold (Illumina, Inc) kit using TruSeq CD Index Adapters1 (Illumina, Inc). RNA was sequenced using a 75-base paired-end (2 × 75 bp PE) dual index read format on the HiSeq4000 (Illumina, Inc.) according to the manufacturer’s instructions, as described in *SI Appendix*, *Supplementary Methods*.

### Lipid Extraction.

Wounds were harvested and homogenized as outlined in *SI Appendix*, *Supplementary Methods*. Internal standards (5 ng each of PC 14:0_14:0 and PE 14:0_14:0) and 5 μL of eicosanoid internal standards were added. Samples were extracted using a solvent extraction (eoxPL) and solid phase extraction (oxylipins) as outlined in *SI Appendix*, *Supplementary Methods*. Lipids were reconstituted using methanol and stored at −80 °C until LC/MS/MS.

### LC/MS/MS Analysis of Oxylipins and eoxPL.

Lipids were quantified using reverse phase LC/MS/MS as described in *SI Appendix*, *Supplementary Methods*. Assay parameters are provided in *SI Appendix,* Table S3 and (39) for oxylipins and *SI Appendix,* Table S5 for eoxPL. For chiral analysis, lipids were separated using a Chiralpak IA-U column (50 × 3.0 mm, Diacel) in reverse phase mode, with assay parameters as for oxylipins.

### Gel Zymography for MMP Activity.

Wounds were snap frozen, then homogenized and analyzed using Novex™ 10% Zymogram Plus (Gelatin) gels (Thermo Fisher), as described in *SI Appendix*, *Supplementary Methods*.

### Cell Transfection and Reporter Assays.

HEK293 cells were transfected with mouse PPARγ and the *Firefly* luciferase under the control of 3× Ppar Responsive Element (PPRE) (40), as described in *SI Appendix*, *Supplementary Methods*.

## Results

### Tissue 12/15-LOX Is Upregulated by Wounding, Associated with Higher Macrophage Numbers and Induction of Interleukins-4 (IL-4) and -13 Signaling.

**Fig. 1.**
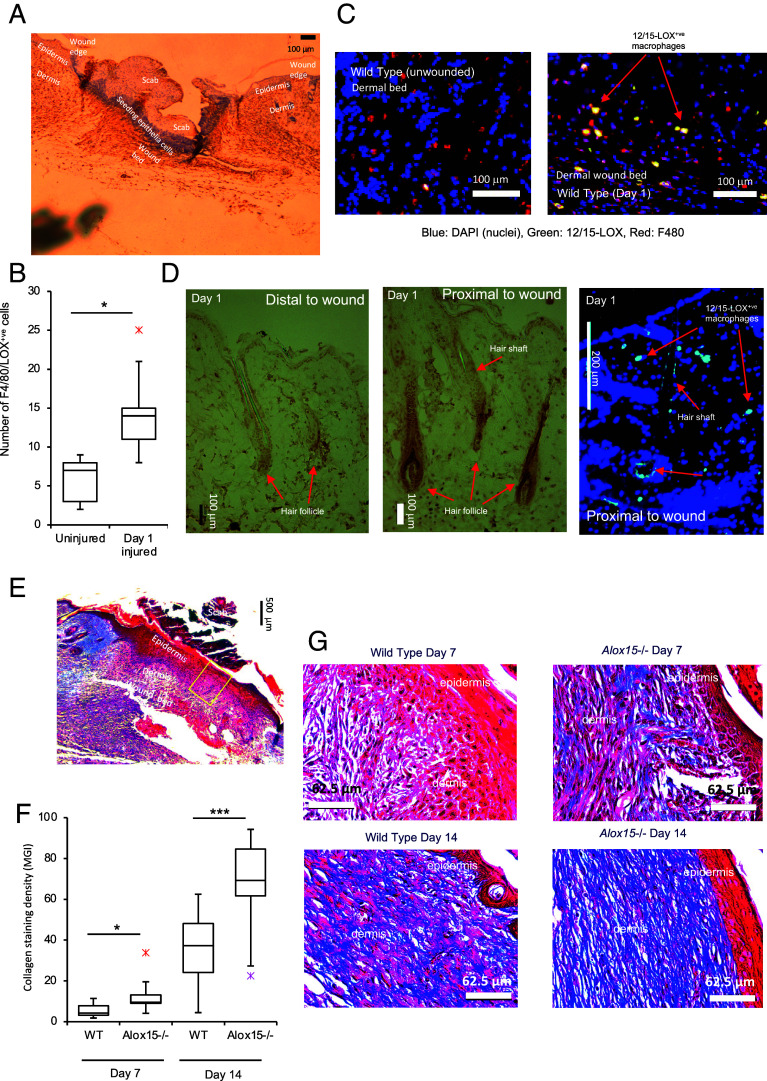
Wounding increases macrophage and hair follicle 12/15-LOX expression, and collagen levels. Panel (*A*) Representative image of wound architecture. Panel (*B*) Induction of macrophage 12/15-LOX by wounding 12/15-LOX^+ve^(green)/F4-80^+ve^(red)/DAPI^+ve^(blue) cells were measured at day 1 post wounding (n = 5/group), data were analyzed using one-way ANOVA with the Tukey post hoc test *P < 0.05, **P < 0.01. Panel (*C*) Representative images from Panel (*B*). Panels (*D*) Expression of 12/15-LOX in hair follicles near the wound edge postwounding. Hair shafts are shown on day 1, wild-type mice. The *Left* and *Center* panels show 12/15-LOX DAB^+ve^ staining, and the *Right* panel shows 12/15-LOX^+ve^ green fluorescent staining (with DAPI counterstain). Panel (*E*) Representative image of wound at low magnification showing region stained for collagen. Panel (*F*) Collagen is elevated in *Alox15*^−/−^ on days 7 and 14. Wounds were harvested and analyzed for collagen using Masson’s Trichrome staining (collagen: blue, epithelial cells: deep red, nuclei: black, noncollagen structures: pink) and pixels counted (n = 10-14/group). Unpaired Student’s *t* test, comparing WT and *Alox15*^−/−^ separately *P < 0.05, ***P < 0.005. Panel (*G*) Representative images from Panel (*F*).

The typical architecture of a wild-type mouse punch wound shows the dermis, wound bed, scab, and wound edge (Fig. 1*A*). Wounding caused a significant increase in 12/15-LOX^+ve^ cells in the skin at 24 h (Fig. 1 *B*–*D*). Most expression was associated with tissue-localized F480^+ve^ macrophages (Fig. 1*C*). 12/15-LOX was also induced in stem cells located at the base of hair follicles adjacent to the wound but not distal (Fig. 1*D*). This suggests that a soluble mediator signaling in response to wounding may be responsible, and that Alox15 is upregulated early postwounding in both cell types. IL-4/IL-13 strongly upregulate macrophage *Alox15* (41, 42). To test for their involvement, RNA-seq data were screened. Although *Il4* and *Il13* expression did not change, their respective receptors were strongly induced at day 4 in both wild-type and *Alox15^−/−^* wounds (*SI Appendix*, Fig. S1), also usually falling back to baseline levels by day 7. Significant induction of the IL-4R-inducible gene *Plod2* (43) evidenced that the pathway was also activated in the model (*SI Appendix*, Fig. S1). The total number of F480^+ve^ monocytes/macrophages in the wound on Day 1 was not impacted by *Alox15* deletion, although there was some reduction later, on Days 4 and 7 (*SI Appendix*, Fig. S2 *A* and *B*). In contrast, neutrophil numbers in the subendothelial compartment were unaffected by *Alox15* deletion (*SI Appendix*, Fig. S2*C*).

### *Alox15* Deletion Alters the Phenotype of the Healing Wound, Promoting Fibroblast Stem Cell Proliferation and Differentiation.

Healing is characterized by stem cell and fibroblast proliferation, collagen deposition, reconstituting the underlying tissue, as well as epithelial migration and differentiation to form a new covering. Here, smooth muscle actin (myofibroblast marker) and collagen deposition were elevated in *Alox15−/−* (*SI Appendix*, Fig. S2 *D* and *E* and Fig. 1 *E*–*G*). This was mainly noted during the remodeling phase (day 14), where the majority of the dermal layer in *Alox15^−/−^* wounds was collagen-dense (Fig. 1*G*). Next, we profiled SSEA3 (stem cells) and the nuclear protein Ki-67 (proliferation) in wound beds at day 4. Both were increased in *Alox15^−/−^,* with SSEA3 being significantly higher, suggesting that the healing wound at this early stage has a higher number of actively proliferating stem cells (*SI Appendix,* Fig. S2 *F*–*H*). We next determined re-epithelialization of the wound during the inflammatory stage (day 4) using cytokeratins 10 (C10, pink) and 14 (C14, green), which indicate epithelial (keratinocyte) cell migration from the wound edge into the wound bed. Basal keratinocytes, which are mitotically active, express C14, but during differentiation, they lose C14 and upregulate C10 (44). At day 4, the migratory distance of C14^+ve^ and C10^+ve^ epithelial cells into the wound edge was similar for both strains (*SI Appendix,* Fig. S3 *A* and *B*). Overall, this suggests that wound bed keratinocyte differentiation is not impacted by *Alox15^−/−^*. Furthermore, the phenotype of nonwounded skin was similar, where in both strains, C14 iexpressed lower in the epithelium, associated with hair bundle cells, with C10 mainly in terminally differentiated (dead) keratinocytes (corneocytes) on the surface (*SI Appendix,* Fig. S3*C*). Overall, the data indicate that while fibroblast and stem cell differentiation and proliferation in the dermis are impacted, keratinocyte differentiation on the surface of the wound is not significantly affected by *Alox15* deficiency.

### Elevated TGF-β/IFNγ/IL6 Activity Is Seen in the Absence of *Alox15*.

**Fig. 2.**
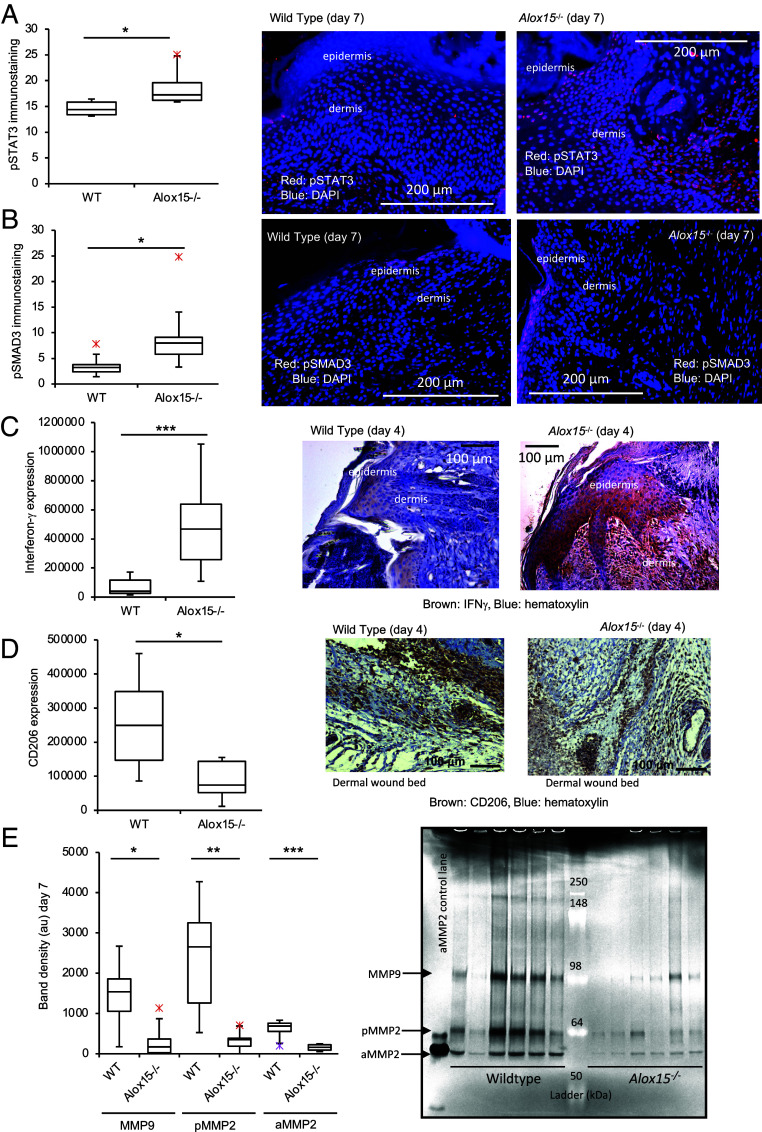
*Alox15^−/−^* wounds show elevated pSTAT3, pSMAD3, and INFγ but decreased CD206 and MMP activities. Panel (*A*) *Alox15*^−/−^ wounds show elevated pSTAT3. pSTAT3 was measured using fluorescence immunohistochemistry. n = 5-6/group. Panel (*B*) *Alox15^−/^*^−^ wounds show elevated pSMAD3. pSMAD3 was measured using fluorescence immunohistochemistry. n = 10-11/group. Panel (*C*) *Alox15*^−/−^ wounds show elevated IFNγ. IFNγ was measured using DAB immunohistochemistry. n = 9/group (4 to 6 fields per wound). Panel (*D*) *Alox15*^−/−^ wounds show reduced CD206 expression. CD206 was measured using DAB immunohistochemistry. n = 5/group (3 to 6 fields per wound). For all panels, data were analyzed an unpaired *t* test, *P < 0.05, **P < 0.01. *Right* panels show representative images for all the proteins analyzed. Panel (*E*) MMP activities are reduced in *Alox15*^−/−^ wounds. MMP activities were measured using zymography. n = 6/group. The ladder shows proteins corresponding to 250, 148, 98, 64, and 50 kDa. Image J was used to calculate the density of each band. The gel is shown (*Right*). The impact of *Alox12^−/−^* was analyzed using an unpaired *t* test, mean ± SEM, *P < 0.05, **P < 0.01, ***P < 0.005.

Next, a series of inflammatory pathways were profiled. Protein expression of IL-6 was slightly but not significantly higher (*SI Appendix,* Fig. S3*D*), but there was significantly elevated pSTAT3 and pSMAD3 (activated by TGF-β) detected in *Alox15^−/−^* wounds (Fig. 2 *A* and *B*). Increased IFNγ was found, primarily on epithelial cells, while conversely, CD206/mannose receptor (a marker of M2 cells), was somewhat reduced (Fig. 2 *C* and *D*, day 4). Taken together with the collagen, fibroblast, and proliferation data, a pro-inflammatory/pro-fibrotic phenotype is suggested for *Alox15*^-/-^.

### *Alox15^−/−^* Wounds Show Reduced Matrix metalloproteinase (MMP) Activities during Wound Healing.

MMPs are collagenases that regulate the extracellular matrix architecture during wound healing by removing and recycling collagen. Since *Alox15^−/−^* wounds showed elevated collagen deposition, zymography evaluated the activity of critical isoforms, MMP2 (active and pro-forms) and MMP9 on day 7. For all three, collagenase activity was significantly reduced in *Alox15^−/−^*wounds (Fig. 2*E*).

### The Temporal Profile of Oxylipins Is Altered by *Alox15^−/−^* with Many Lipids Reduced/Absent.

**Fig. 3.**
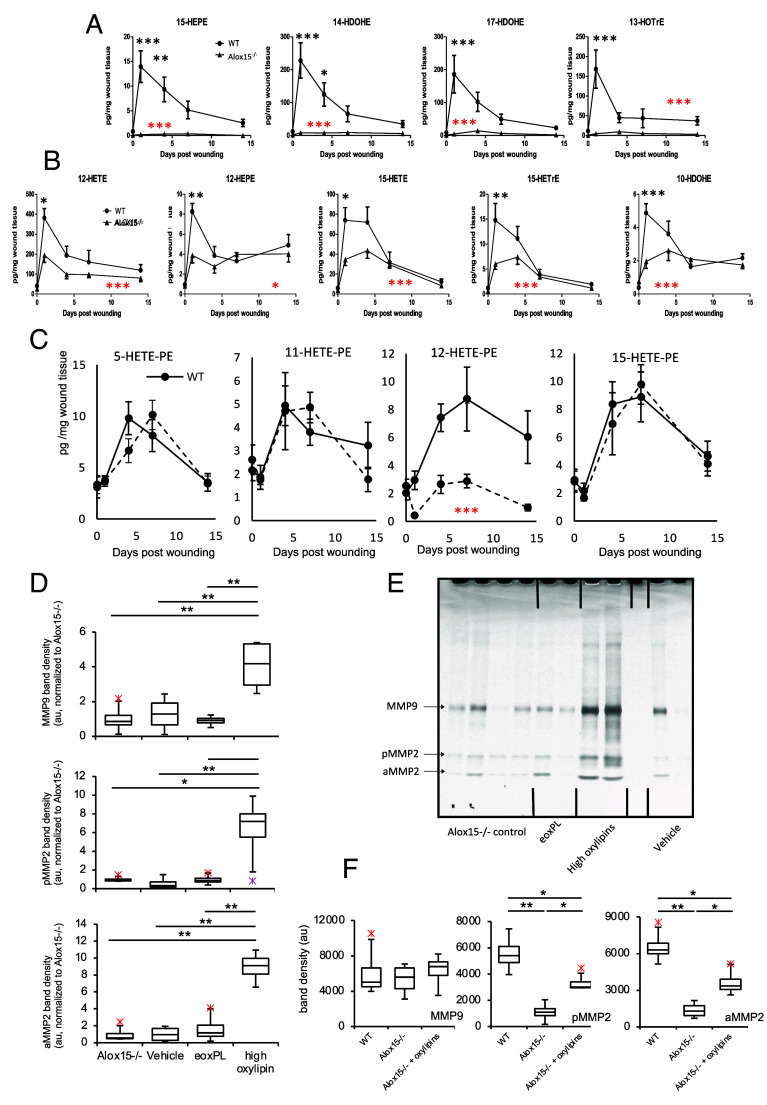
*Alox15^−/−^* wounds show lower levels of many oxylipins and 12-HETE-PEs, while physiological levels of high oxylipins restore MMP activity. Panels (*A* and *B*) Oxylipins are rapidly elevated postwounding, but many are reduced in *Alox15*^−/−^ wounds. Oxylipins were measured using LC/MS/MS as outlined in *Methods*. n = 6 samples/time point, with 4 wounds pooled/sample. For all panels, differences between groups were analyzed using two-way ANOVA (red stars), with the Bonferroni post hoc test between individual time points (black stars), mean ± SEM, *P < 0.05, **P < 0.01, ***P < 0.005. Panel (*C*) HETE-PEs elevate during healing, peaking on day 7, with significant loss of 12-HETE-PE isomers in *Alox15*^−/−^ wounds. Oxidized phospholipids were measured using LC/MS/MS as outlined in Methods (n = 5 samples/time point, with 4 wounds pooled/sample). Unpaired *t* test, *P < 0.05, **P < 0.01, ***P < 0.005. Panel (*D*) High oxylipins restored MMP activities in wounds from *Alox15*^−/−^ mice. Post wounding, lipids were added to wounds as indicated in *Methods* every second day. Wounds were harvested and analyzed for MMP activities using zymography as in *Methods* (n = 4, 4 to 5 wounds pooled/sample). Panel (*E*). A gel showing representative data from Panel (*C*). Panel (*F*) High oxylipins restore MMP activities to wild-type level. Wounds were harvested and analyzed for MMP activities using zymography as in *Methods* (n = 4, 4 to 5 wounds pooled/sample). For Panels (*C* and *D*) ANOVA with the Tukey post hoc test was used, *P < 0.05, **P < 0.01, ***P < 0.005.

Using reverse phase LC/MS/MS, ~100 oxidized fatty acids were profiled in wound tissue, including well-known prostaglandins, thromboxane, eicosanoids, docosanoids, and several SPMs. A summary of the 68 detected is shown (*SI Appendix,* Fig. S4*A*). Several monohydroxy lipids were strongly elevated at day 1 but absent in *Alox15^−/−^* wounds (15-HEPE, 14-HDOHE, 17-HDOHE, 13-HOTrE) (Fig. 3*A*). 12-HETE/12-HEPE, and 15-HETE/15-HETrE, which are generated by 12/15-LOX but also by platelet 12-LOX and COXs, were also highly increased and were reduced by 50% in *Alox15^−/−^* wounds (Fig. 3*B*). All these peaked at day 1, then declined, paralleling early transient expression of 12/15-LOX in the wound. This indicates that 12/15-LOX oxylipin generation is acute and transient, peaking during the inflammatory phase, with lipids reduced toward basal levels during healing.

Several prostaglandin dehydrogenase (PGDH) metabolites generated from oxidation of HODEs and HETEs (9-, 13-oxoODE, and 15-oxo-ETE) were similar in both strains, but peaked at day 4 with higher levels in *Alox15*^−/−^ (*SI Appendix,* Fig. S4*B*). Lipids from 5-LOX (5-HETE, LTB_4_) and COX (PGE_2_, PGD_2_, 11-HETE, 11-HEPE, 15d-PGJ_2_, TXB_2_, and other PGE_2_ isomers) were strongly elevated at day 1 and declined after but were not impacted by *Alox15*^−/−^ (*SI Appendix,* Fig. S4*B*). The LA products 9- and 13-HODE were elevated early and were slightly lower on day 1 in *Alox15*^−/−^ (*SI Appendix,* Fig. S5). Several cytochromeP450/soluble epoxide hydrolase (sEH) metabolites were detected at very low amounts with small increases around day 4 which fell by days 7 and 14 (5,6-diHETrE, 8,9-diHETrE, 11,12-diHETrE, 14,15-diHETrE, LTB4, 5,6-EET, 7,8-EpDPA, 13,14-EpDPA) (*SI Appendix,* Fig. S5). Last, 9,10-EpOME and 12,13-EpOME elevated beyond day 4, although levels fluctuated significantly, similar to their sEH metabolites 9,10-diHOME and 12,13-diHOME (*SI Appendix,* Fig. S5). None were reduced by *Alox15*^−/−^ indicating they were from other biochemical or nonenzymatic pathways. Indeed, many from CYP/sEH were significantly higher at day 4, potentially due to differences in substrate availability. Out of several SPM monitored, only trace amounts of resolvinD5 were detected, with its detailed structural analysis including chiral chromatography shown in *SI Appendix*, *Supplementary Methods and Data*.

### Enzymatically Oxidized Phospholipids (eoxPL) Are Generated during Wound Healing, Peaking during the Proliferative Stage, with 12-HETE-PEs Significantly Impacted by *Alox15* Deficiency.

Free oxylipins generated by COXs or LOXs are also formed as complex lipids attached to phospholipids (PL), termed eoxPL. The most abundant are HETE-containing phosphatidylethanolamine (PE), either generated by direct attack on PE by 12/15-LOX or by esterification of newly formed HETEs to lysoPE (10). 12/15-LOX generates 12-HETE-containing eoxPL, while 5-HETE-PE arise via 5-LOX (neutrophils) (8, 45). Platelet 12-LOX is a source of 12-HETE-PEs (46). 15-HETE-PEs form either via 12/15-LOX, or through esterification of 15-HETE by COX, which is also a source of 11-HETE-PEs. Following wounding, sustained elevations of 5, 11, 12, and 15-HETE-PEs occurred, peaking on day 4, then declining (Fig. 3*C*). Each represents a series of isomers differing by *sn1* fatty acid, with 3-4 per HETE isomer (Fig. 3*C* and *SI Appendix,* Fig. S6*A*). 8-HETE-PE were below LOQ, indicating that there is little/no nonenzymatic oxidation and confirming the others are from LOX and COX. Overall, esterified HETEs were less abundant than their corresponding free acids. Consistent with generation by 12/15-LOX, 12-HETE-PE was reduced by >50% in *Alox15*^−/−^ wounds, while others were unaffected (Fig. 3*C*). Individual 12-HETE-PEs were all significantly lower in *Alox15*^−/−^ wounds (*SI Appendix,* Fig. S6*B*). These data confirm an enzymatic origin, but similar to free HETE, a significant amount is from other sources, such as platelet 12*S*- or skin 12*R*-LOXs. All HETE-PEs peaked around days 5 to 10, later than free acid HETEs (Fig. 3*C*). Thus, as free HETEs declined, the esterified forms were elevating. This may reflect onset of esterification processes driven by Lands cycle.

### MMP Activities and Collagen Deposition Are Restored to Wild-Type Levels in *Alox15^−/−^* Wounds by High Abundance Oxylipins, but Not eoxPL.

Oxylipins are not generated in isolation but as complex mixtures in vivo. Here, lipidomics data informed the formulation of relevant mixtures to be added to healing *Alox15^−/−^* wounds (*SI Appendix,* Table S1). *High oxylipins* contained lipids generated acutely in higher amounts that were relatively deficient in *Alox15*^−/−^ wounds, with amounts added via topical dermal delivery aiming to match the maximum levels detected postwounding [12-HETE, 17-HDOHE, 13-HOTrE, 15-HETE, 12-HEPE, 12-oxo-ETE, 15-HEPE, 12(13)-EpOME, 13-HODE, 14-HDOHE]. Wounds treated with *high oxylipins* demonstrated increased levels of MMP9, aMMP2, and pMMP2 (Fig. 3 *D* and *E* and *SI Appendix,* Fig. S7*A*). Notably, this treatment reversed the phenotype, resulting in MMP activities that were significantly elevated to levels between those of WT and Alox15^−/−^ (Fig. 3*F* and *SI Appendix,* Fig. S7*B*). No impact was seen with a pharmacological dose of PE 18:0a/12-HETE, an eoxPL which was 50% reduced by *Alox15* deletion (Fig. 3 *D* and *E* and *SI Appendix,* Fig. S7*A*). Next, the ability of lipids to reduce the accelerated collagen deposition of *Alox15*^−/−^ was tested. Here, treatment with vehicle alone caused a nonsignificant increase in collagen, but the high oxylipin preparation completely suppressed this. However, as for MMPs, there was no impact of eoxPL (*SI Appendix*, Fig. S4 *A* and *B*).

### Transcriptional Analysis Reveals Reduced Anti-Inflammatory Lipid Metabolism in *Alox15^−/−^* Healthy Skin but a Relatively Normal Acute Response to Wounding.

**Fig. 4.**
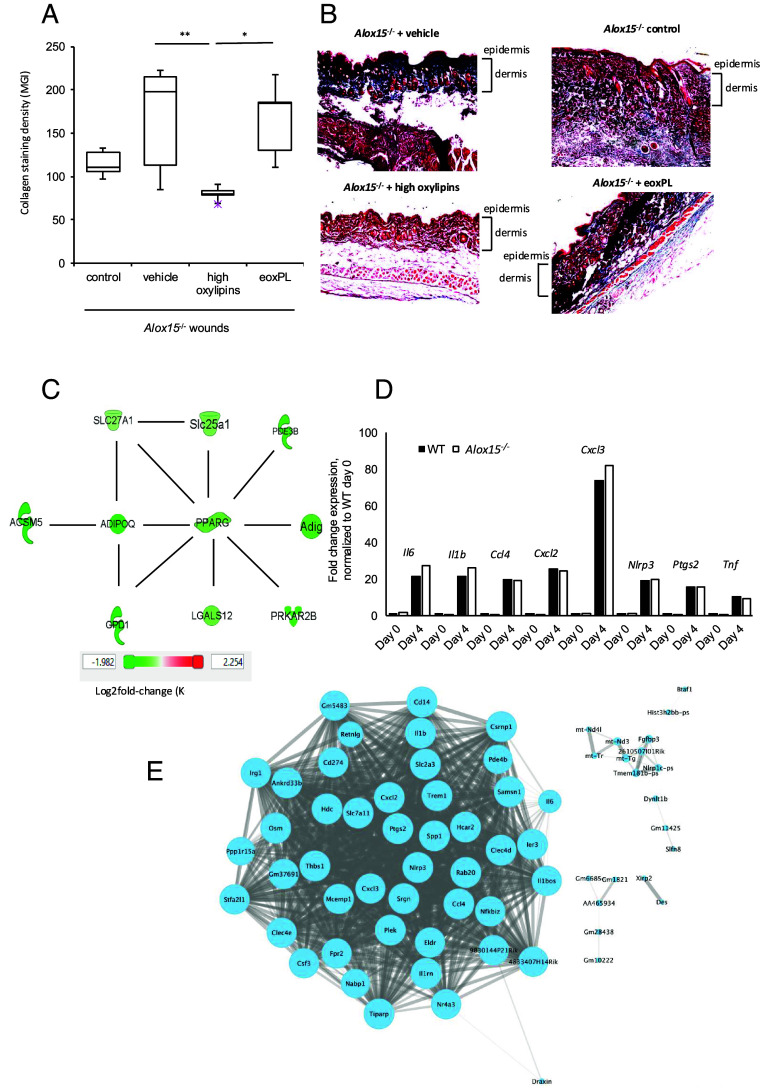
High oxylipins dampen collagen deposition, while unwounded *Alox15^−/−^* skin shows reduced PPARγ activity but a normal pro-inflammatory response to wounding, while a series of highly correlated genes fail to revert to baseline in *Alox15^−/−^* wounds at Day 7. Panel (*A*) Collagen generation is dampened by high oxylipins. Post wounding, lipids were added to wounds as indicated in *Methods* every second day. Wounds were harvested at Day 7 and analyzed for collagen using Masson’s Trichrome staining (collagen: blue, epithelial cells: deep red, nuclei: black, noncollagen structures: pink) and pixels counted (n = 5-6 wounds/group. Panel (*B*) Representative images from Panel (*A*). Panel (*C*) Unwounded *Alox15*^−/−^ skin shows significantly reduced PPARγ/adiponectin expression/activity. RNA-seq was carried out as indicated in Methods on nonwounded skin (n = 3 to 4 samples per group, each sample was a pool of 4 wounds per animal with 2 animals per pool = 8 wounds per sample). All genes shown are significantly reduced in *Alox15^−/−^* skin and are either controlled by or regulate PPARγ/adiponectin. Panel (*D*) Upregulation of a series of canonical “inflammatory” genes is preserved in *Alox15*^−/−^ wounds. Gene expression was normalized for each gene to its Day 0 mean value, then expressed as fold-change (n = 3 to 4 per group) each sample was a pool of 4 wounds per animal with 2 animals per pool = 8 wounds per sample). For Panel (*A*), ANOVA with the Tukey post hoc test. Panel (*E*) A large number of genes that are significantly different between wild-type and *Alox15*^−/−^ wounds at Day 7 highly correlate across the whole timecourse, indicating coordinated regulation. Genes that were found to be significantly differentially expressed at Day 7 were analyzed in Cytoscape, using their expression levels for the entire time-course, with correlation [r] > 0.8 shown.

To identify transcriptional networks modulated by *Alox15^−/−^,* RNA-seq was performed on day 0 (healthy tissue), and on days 4 and 7 postwounding. At Day 0, 143 genes were significantly different between the two strains (adjusted *P*-value < 0.05) (*SI Appendix,* Table S6). Ingenuity Pathway Analysis (IPA) identified lipid metabolism as highly represented, and a subset of genes in that network is shown (Fig. 4*C*). Significant downregulation of *Adipoq* (adiponectin) and *Pparg* (PPARγ) was seen, associated with a reduction in genes that either control or are controlled by these (Fig. 4*C*) (47–58). PPARγ is a transcription factor that induces adiponectin (59), and it responds directly to oxylipin ligands generated by *Alox15,* including several HODEs, HETEs, and HDOHEs (13, 16–19) upregulating transcription through classical PPAR-response element (PPRE) signaling. Adiponectin is a hormone and adipokine centrally involved in metabolism, protective against a number of inflammatory conditions, such as atherosclerosis and type 2 diabetes (60, 61). Both are crucial in mediating anti-inflammatory actions, such as inhibition of pro-inflammatory NF-κB signaling and NLRP3 (62, 63). Additional down-regulated genes in the network include regulators of lipid metabolism, such as *Slc27a1* (import of long-chain fatty acids), *Acsm5* (acyl-CoA synthetase medium-chain family member 5), and *Gpd1* (which regulates lipid metabolism). Two genes that regulate adipose tissue development are also down-regulated: *Adig* (adipogenin) and *Lgals12*. Importantly, reduced basal expression of *Adipoq* and *Pparg* indicates that *Alox15^−/−^* tissues would struggle to mount the anti-inflammatory response required to counterbalance inflammation during the later wound healing phase in which PPARγ signaling is known to play a role (64). Wounding itself did not stimulate PPRE transactivation since *Adipoq*, *Adig,* and *Slc27a1* were not induced in either wild type or *Alox15^−/−^*wounds, instead falling to levels similar to *Alox15^−/−^* wounds (*SI Appendix,* Fig. S8). At day 4, a comparison between WT and *Alox15^−/−^* wounds showed that around 42 genes were significantly different (*SI Appendix,* Table S7). These genes were not obviously functionally related. However, the classic inflammatory response, measured by genes that include *Tnfa, Il1b, IFNg, Nlrp3, Cxcl2, Ccl4*, and *Il6* was preserved in both strains, indicating normal onset of inflammation (Fig. 4*D*).

### Late Wound Healing in *Alox15^−/−^* Wounds Shows a Failure of Inflammation to Reduce to Basal Levels, with Many Pro-Inflammatory Genes Remaining Upregulated.

**Fig. 5.**
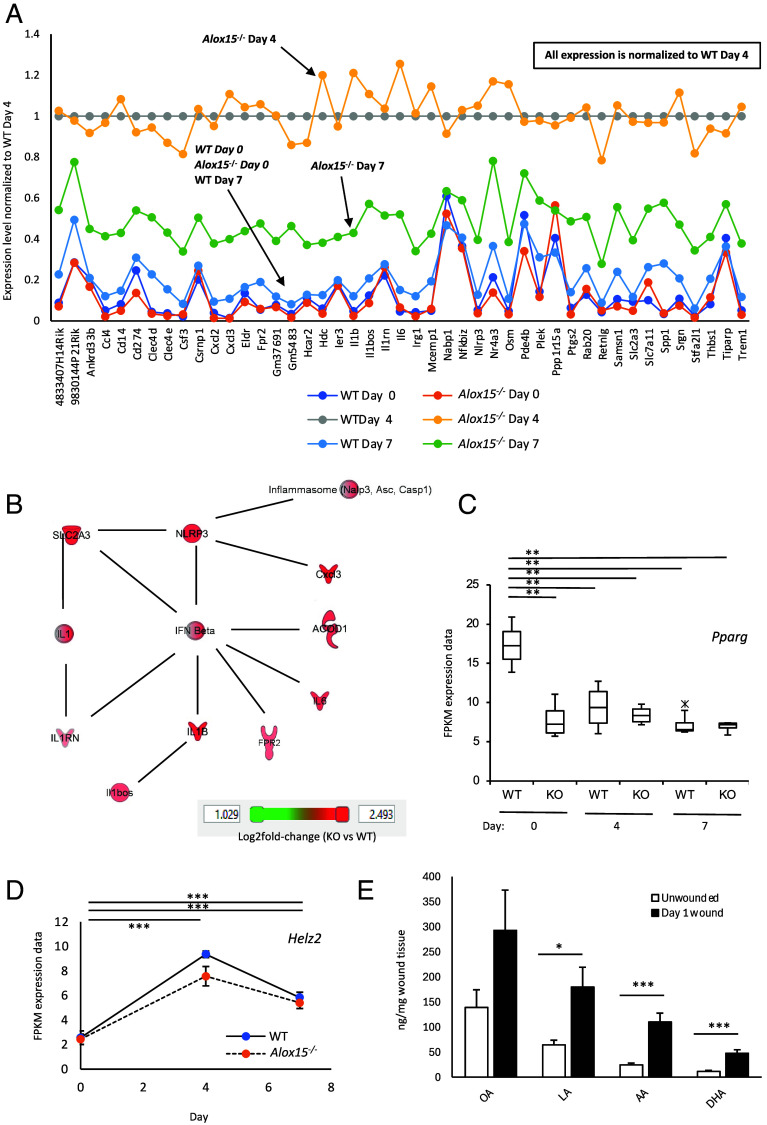
Genes that fail to revert at day 7 in *Alox15^−/−^* wounds remain 50% elevated above baseline, and many are controlled through NLRP3, IFNβ and IL-1, PPARγ expression is not upregulated during wounding, while *Helz2* and unsaturated fatty acids are increased. Panel (*A*) All genes from the highly correlated network in Fig. 4*E* typically remain 50% elevated. Data from gene expression of highly correlated genes were averaged and normalized to day 4, wild-type mean (the inflammatory response level) (n = 3 to 4 per group). Panel (*B*) IPA network analysis of genes that are significantly different at Day 7 reveals significantly higher levels of Inflammasome, IFNb, and IL-1 pathways. Gene expression data from Day 7 were analyzed using IPA. Panel (*C*) *Pparg* expression are not increased during wounding. Transcriptional data on *Pparg* were compared across the timecourse (n = 3 to 4 per group). For all gene expression data, Student’s *t* test, followed by Benjamin Hochberg correction: *P < 0.05, **P < 0.01, ***P < 0.005. Panel (*D*) Data from gene expression are shown for WT and Alox*15^−/−^* wounds during the time course (n = 3 to 4 per group). For all gene expression data, Student’s *t* test, followed by Benjamin Hochberg correction: *P < 0.05, **P < 0.01, ***P < 0.005. Panel (*E*) UFA are significantly elevated in early wounds. Lipids were extracted from unwounded and wounded (day 1) skin, extracted and derivatized as outlined in *Methods* before UFA were quantified using LC/MS/MS (n = 3, unwounded, 4, day 1 wounded).

At day 7, 79 genes were significantly different between the strains, with 60 higher in *Alox15^−/−^*wounds than WT (*SI Appendix,* Table S8). A Cytoscape analysis was performed using the whole-time course dataset for the 79 genes (day 0, 4, 7, and both strains), and a subgroup of 45 was seen to strongly correlate, suggesting their behavior was coordinated during the entire wounding and healing process (Fig. 4*E*). These included several pro-inflammatory genes such as *Ccl4, Cd14, Cd274, Clec4d, Clec4e, Csf3, Cxcl2, Cxcl3, Fpr2, Il1b, Il6, Irg1, Nfkbiz, Nlrp3, Ptgs2, Retnlg, Trem1,* and *Osm*. Many are involved in macrophage-driven inflammation, and all were highly upregulated in both WT and *Alox15^−/−^*wounds on day 4. In *Alox15^−/−^*, these all failed to return to basal levels by day 7, with their transcription remaining around 50% of the day 4 levels (Fig. 5*A* and *SI Appendix,* Fig. S9). This contrasts with WT wounds, where these fully returned to basal levels by day 7 (Fig. 5*A* and *SI Appendix,* Fig. S9). IPA of this subgroup of genes demonstrated that many are upregulated through common mechanisms, such as NFκB. For example, within this group, an IPA subnetwork predicted higher activity of the IL-1, IFNβ and Inflammasome pathways in the *Alox15^−/−^*wounds. Importantly, IL-1, IFNβ and Inflammasome are all well-known to be downregulated by PPARγ (Fig. 5*B*). Of the 45 genes identified, 27 are either directly induced by or are activators of NF-κB/NLRP3 (*SI Appendix,* Table S9), while several are down-regulated by activation/induction of PPARγ either directly or via inhibition of NF-κB, including *Il6, Nlrp3,* and *Il1b* (65–67). Last, it was seen that while *Pparg* expression was not induced by wounding, its expression fell in WT wounds to levels similar to those seen in *Alox15^−/−^* (Fig. 5*C*). This suggests that the lack of PPARγ signaling during the healing response results from a relative deficiency in 12/15-LOX-derived ligands, explaining why their supplementation could reduce the fibrotic phenotype in *Alox15^−/−^* wounds.

### TGF-β Pathway Is Upregulated by Wounding in Both Strains, but miR-21 Fails to Return to Basal Levels in *Alox15^−/−^*.

The fibrosis phenotype seen in *Alox15^−/−^* wounds is consistent with elevated TGF-β signaling, which is known to be inhibited by ligand-activated PPARγ (68–70). In both WT and *Alox15^−/−^* strains, significantly increased TGF-β expression was seen along with TGF-inducible genes, *Serpine1*, *Tnc,* and *miR-21a* following wounding (*SI Appendix,* Fig. S10). This indicates upregulation of TGF-β signaling, consistent with previous studies (71–73). However, this response was not affected by *Alox15* deficiency, apart from the pro-fibrotic microRNA, miR-21a, which completely failed to return to basal levels by day 7 (*SI Appendix,* Fig. S10). This suggests that the increased collagen, pSMAD3, and pSTAT3 in late *Alox15*^−/−^ wounds are not due to dysregulated induction of TGF-β during the early wound response, but rather a failure of miR-21 to return to basal levels during resolution.

### “High Oxylipins” Generated during the Wound Response Induce Transcriptional Activity of PPARγ in a Reporter Assay.

To determine whether the abundant oxylipins generated by 12/15-LOX during wounding activate PPARγ, the *high oxylipin* mixture was tested in a reporter assay, with HEK293 cells expressing mouse PPARγ and *Firefly* luciferase under control of 3x Ppar Responsive Element (PPRE) (40) exposed to the lipids for 24 h. Doses were informed by oxylipin amounts detected in wounds in this study and studies which show in vitro oxylipin activation of PPARγ in the 10 to 100 μM range (13, 15–18). First, amounts detected in a wound on Day 1 were tested (*SI Appendix,* Table S1). These were diluted into 50 μL media (0.4 to 1.2 μM total oxylipins). However, at these doses, PPARγ was not reliably activated (not shown). Thus, we next tested amounts previously shown to activate PPARγ in vitro. The most abundant lipid in our mixture was 12-HETE, ranging from 12 to 36 μM at the doses tested, with total oxylipins at 40 to 120 μM (*SI Appendix,* Table S1). PPARγ activation was seen for all oxylipin doses, with 80 μM showing a significant increase (*SI Appendix,* Fig. S11*A*). This is in line with previous reports that many individual 12/15-LOX products can act as low-affinity PPARγ ligands at these concentrations and that *Alox15* deficiency leads to loss of macrophage PPARγ activation (13, 15–19). Overall, the mixture of *Alox15*-oxylipins can activate PPARγ in a complex mixture, in full agreement with previous literature. As amounts of added oxylipins were higher than believed generated in vivo, we next tested whether coexpression of RXR along with its ligand 9-cis-retinoic acid could sensitize PPARγ to oxylipin activation in the reporter assay. However, while overall there was higher PPARγ activity in the presence of RXR/9-cisRA, there was no sensitization to 17-HDOHE seen (*SI Appendix,* Fig. S11*B*).

### Wounding Induces Significant Increases in Gene Expression of the PPARγ Coactivator Pdip1/*Helz2*/Pric285 and Levels of Unsaturated Fatty Acid PPARγ Ligands.

We noted that amounts of *Alox15* oxylipins used in vitro were higher than detected in vivo. However, exact wound oxylipin concentrations are not possible to determine, and it is not known how much enters the cells to bind and activate PPARγ in vitro. Furthermore, the ability of oxylipins to activate PPARγ may be influenced by protein coactivators in vivo or additional ligands such as unsaturated fatty acids (UFA) (74–78). First, the expression of known coactivators was interrogated in transcriptional data (75). Several were detectable at the mRNA level at all time points, including *Rxra, Rxrb, Rxrg, Ep300, Ncoa1, Ncoa2, Ncoa3, Ppargc1b, Plcl1, Helz2, Chd9,* and *Smarcd3* (*SI Appendix,* Fig. S11*C*). While most did not change significantly during wounding, one showed consistent and significant upregulation in both WT and *Alox15^−/−^* wounds on both days 4 and 7, compared to day 0 (Fig. 5*D*), with the highest expression on day 4. PPARγ-DBD-interacting protein 1a, HELZ2/PRIC285 (*Helz2*) is a helicase that binds DNA binding domains of PPARγ through its C-terminal region and can enhance PPARγ activation by troglitazone directly (79). Whether HELZ2/PRIC285 can similarly enhance the ability of oxylipins to bind and activate PPARγ remains to be tested. Next, we tested whether wounding alters levels of UFA in tissues and found that oleic (OA), linoleic (LA), arachidonic (AA), and docosahexaenoic (DHA) acids all increased between 2.2-fold and 5.3-fold, with LA, AA, and DHA all being significantly elevated (Fig. 5*E*).

### Comparison of Temporal Changes in Gene Expression Indicates Additional Transcription Activators Regulated by *Alox15* beyond PPARγ, Including *Elf4*, *Cebpb*, and *Tcf3*.

Last, RNA-seq was further interrogated for additional *Alox15*-modulated transcriptional regulators, using a temporal analysis. Here, we characterized individual strains separately and identified three candidates (see *SI Appendix*, *Supplementary Methods and Data* for complete analysis). *Elf4* is a known anti-inflammatory transcription regulator that targets several genes in the list, including *Anln, Asf1b, Ccnb2, Cdca3, Cenpa, Cenpe, Cks2, E2f8, Hmmr, Kif4a, Mcm10, Ndc80, Oip5, Rrm2, and Tpx2* (80). *Tcf3* promotes cell migration and wound repair (81), and *Cebpb* is involved in macrophage repair responses and inflammation (82, 83). Many genes mapping to networks that regulate cytokines were also identified, further evidencing the impact of *Alox15* on inflammatory signaling and identifying numerous targets for further study (*SI Appendix*, *Supplementary Methods and Data*).

### Pro-Resolving Signaling by Nrf2 and LXR Is Not Dampened by *Alox15* Deficiency, Confirming Specific Dysregulation of PPARγ.

Aside from PPARγ, other transcriptional networks that are considered anti-inflammatory include the Antioxidant Response Element (Nrf2) and the Liver X Receptor (LXR). To test whether these were also impacted by *Alox15* deficiency, RNA-seq data were interrogated for alterations in genes known to be upregulated by these transcription factors, e.g., *Hmox1, Gclm, Nqo1* (Nrf2) and *Abca1, Srebf1, Oasl1, Trim34a, Trim34b, Marco, Ctla2b, Lpl* (LXR) (84, 85). Aside from *Lpl*, which was higher basally in *Alox15^−/−^* wounds, no significant changes were found between strains, although several, mainly LXR-responsive genes, showed transient and often significant increases on day 4 in both strains (*Abca1, Oasl1, Trim34a, Trim34b, and Ctla2b, Hmox1*) (*SI Appendix,* Fig. S12). These data suggest that while LXR may also be activated by injury, only PPARγ is selectively impacted by *Alox15* deficiency and required for dampening inflammation in the model.

## Discussion

In this study, lipidomics and transcriptomics of *Alox15^−/−^* wounds reveal significant pathways impacted by the enzyme during wound healing. Overall, many pro-inflammatory genes highly induced by wounding fail to return to basal expression in *Alox15^−/−^* wounds. Taken with our phenotypic data and the response to known PPARγ ligands from this pathway, an intrinsic anti-inflammatory action of 12/15-LOX, driven by PPARγ, is lost in *Alox15^−/−^*mice. This was mapped to a failure to dampen NLRP3/inflammasome and TGF-β signaling during resolution, which together will support an uncontrolled fibrosis response, almost identical to that previously reported in several studies on PPARγ-deficient mice (described below) (67, 86, 87). At day 7, *Alox15^−/−^* wounds express significantly higher levels of several genes that drive fibrosis, for example, IL-1β and IL-6 which also work in tandem with TGF-β (88–90). These cytokines directly induce miR-21 (91, 92) a well-known microRNA promoter of fibrosis and cell proliferation (93), which is induced by TGF-β (94), and which we found also failed to resolve in *Alox15^−/−^* wounds. miR-21 increases fibrosis by enhancing TGF-β activity, through regulating SMAD expression (93), and downregulating Spry1 which increases ERK-MAP kinase signaling (95, 96). In resolving skin wounds, while initially requiring TGF-β (for its induction), we suggest that miR-21 then acts later by regulating protein translation, and/or induction of fibrosis genes, as seen from day 7 onward. The higher levels of miR-21 are consistent with our observations of elevated collagen and pSMAD at day 7 in *Alox15^−/−^* wounds (97, 98). Supporting this idea, PPARγ can negatively regulate miR-21 through preventing its expression (99). Taken together, our data propose a positive feedback loop involving IL-6, IL-1β and miR-21 working together to drive fibrosis in *Alox15^−/−^* wounds, in the absence of PPARγ’s anti-inflammatory ligands. Transcription factors, including *Elf4*, *Tcf3,* and *Cebpb* may also play important roles, but their functions remain to be established, while role for LXR or Nrf2 appears unlikely.

We found that acute upregulation of *Alox15* was likely dependent on IL-4/IL-13, both of which are well known as potent inducers of the enzyme in macrophages (41, 42). Furthermore, previous studies directly linked these cytokines to oxylipin-dependent PPARγ activation, including acting through *Alox15* (13, 100). IL-4 plays an essential anti-inflammatory role in tissue repair, including in healing mouse skin wounds. In agreement with our study, monocytes recruited to sites of injury show a dynamic increase in IL-4Ra expression, and the lack of IL-4Ra in mice leads to a failure of the repair response, characterized by altered collagen fibril formation and crosslinking (43). Significantly, IL-4 also induces PPARγ in macrophages directly (13).

12/15-LOX generates abundant mono-oxygenated oxylipins, eoxPL, and in concert with other LOXs, is proposed to generate rarer, multiply oxygenated SPM via transcellular biosynthesis. However, the lipids primarily responsible for the effects of 12/15-LOX during physiological healing and resolution have been unclear. Its lipids are not generated in isolation but in complex mixtures of varying abundance. However, studies testing their role in inflammation have usually added them singly (21–24). To test the role of the enzyme under physiological conditions, we employed a well-characterized model of wounding that fully heals within 14 d. Transcriptomic, phenotypic, and lipidomic approaches revealed that *Alox15* deletion leads to an accelerated healing response, resulting in a “fibrotic” phenotype. During this period, higher collagen deposition, increased stem cell proliferation and differentiation are observed, along with higher levels of inflammatory markers such as IL-6/pSTAT3, pSMAD3, and IFN-γ. A failure of pro-inflammatory gene expression to reduce back to baseline during the later remodeling phase was found. Our transcriptional data identified a basal deficiency of PPARγ expression and activity and also showed that genes which are upregulated by transcription factors such as NLRP3 or NF-kB (both antagonized by PPARγ) did not revert to basal levels postwounding. Linked with this, wild-type wounds generated large amounts of several known PPARγ ligands via 12/15-LOX (e.g., 17-HDOHE, 12-HETE, 15-HETE, 12-HEPE, 13-HOTrE) during the early inflammatory phase, and critical features of normal wound healing could be restored in *Alox15^−/−^* by supplementing with physiological levels of a mixture of these (13, 15–20, 101). Furthermore, several oxylipins that were deficient in *Alox15^−/−^* wounds are known to dampen IL-6 [12-HETE, 15-HETE, 14-HDOHE, 15-HEPE (7, 15, 102)] and NLRP3 [13-HOTrE (103)], with these effects also likely mediated by PPARγ. Based on our staining data, the most likely cellular sources of *Alox15*-derived lipids will be F480^+ve^ macrophages and stem cells at the base of hair follicles.

While the mixture of oxylipins could activate PPARγ PPRE transactivation in vitro, concentrations needed, although in line with many other studies on PPARγ, appeared to be around 100-fold higher than those found in vivo. This suggests that *Alox15* products alone are not sufficient, and other factors may be required. These could include other oxylipins, UFA, or protein coactivators known to sensitize PPARγ to agonists such as RXR, or proteins encoded by *Ep300, Ncoa1, Ncoa2, Ncoa3, Ppargc1b, Plcl1, Helz2, Chd9,* and *Smarcd3.* Several UFA, along with COX and 5-LOX products known to activate PPARγ were significantly upregulated during wounding, including AA, DHA, LA, PGE_2_, PGD_2_, 15d-PGJ_2_, and LTB_4_ (76–78, 104–106). UFA were considerably more abundant than oxylipins but may be less potent PPARγ activators (77). Also, *Helz2* was upregulated significantly following wounding (79), with transcripts for most other protein regulations detected basally in skin, and present throughout. Apart from RXR, which we ruled out experimentally, any of these could modulate activation of PPARγ by *Alox15*-derived oxylipins in vivo. Last, PPARγ has a recently described alternative binding domain which binds ligands that prevent phosphorylation and inactivation of PPARγ, for example the plant-derived terpenoid, saikosaponin A (107). A role for this was not tested.

PPARγ’s ability to dampen inflammation (via downregulation of TGF-β or NLRP3/inflammasome) is completely distinct from PPRE-transactivation, instead representing ligand-dependent transrepression. Here, PPARγ acts as an E3 ubiquitin ligase, inducing ubiquitination and degradation of NF-κB/p65 (108), as well as interacting directly with NLRP3 to prevent its activation (109). TGF-β signaling inhibition by ligand-activated PPARγ has been suggested to involve dampening Smad-dependent signaling (68–70), with this proposed to occur via epigenetic mechanisms. The effective concentrations of oxylipins that support transrepression by PPARγ are unknown, but if lower than required for PPRE, this could explain the discrepancy. In support, the oxylipins/FFA generated during acute wounding did not induce acute PPRE activity in WT skin in our model, despite being sufficient to mount a significant anti-inflammatory/antifibrotic activity.

Basally, *Alox15^−/−^* skin expressed significantly lower PPARγ. Several reports show that ligands, including oxylipins from *Alox15* can induce PPARγ mRNA and protein, while the PPARγ antagonist T0070907 reduces its expression (110). For example, exogenous 12- or 15-HETE upregulate PPARγ in ischemic rat brain (17), while PPARγ mRNA is induced by dexamethasone in a celecoxib-inhibitable manner. These studies, along with several others, also found that Rosiglitazone or 15d-PGJ_2_ directly induces PPARγ (67, 111–114). In one, the pro-inflammatory phenotype of diabetic wounds was not reversed when PPARγ-deficient wounds were tested (67). Thus, PPARγ ligands, including oxylipins, can induce its expression consistent with the observation that lack of *Alox15* results in loss of the protein, likely through a mechanism involving deficiency of oxylipins. This is consistent with the observations that elicited peritoneal macrophages lacking *Alox15* express constitutively reduced levels of the PPARγ target gene, *Cd36* and that IL-4 induces PPARγ in mouse macrophages (13). Considering that this coincided with reduced levels of many other PPARγ-inducible genes, a role for ligand-activated PPRE transactivation by oxylipins in maintaining basal expression is highly likely. Here, PPARγ is considered an autoregulated gene, responding to ligand activation through a positive feedback loop (115). This particularly relates to isoforms 1,3 and 4, but not 2, and was suggested to involve changes in promoter activity and mRNA stability, although the mechanisms are not fully clarified (111, 112).

Notably, the phenotype seen here is almost identical to that of PPARγ^-/-^ mice, where increased actin, collagen pSMAD3, and an accelerated healing/fibrotic phenotype in skin were described (86, 87) as well as sustained IL-1β expression (67). Also, loss of PPARγ in skin fibroblasts is associated with elevated pSMAD3, while PPARγ agonists directly reduce actin and collagen expression (68, 87). Furthermore, PPARγ blockade elevates MMP1 and MMP9 in fibroblast-like synoviocytes (116), while its activation dampens fibroblast proliferation and differentiation (117). PPARγ also inhibits IL-6, IFN-γ and pSTAT3 expression and prevents fibroblast pSMAD3-dependent collagen synthesis and deposition (118–121). The reduction in CD206 is also consistent with decreased PPARγ activity since it is already known that this transcription factor directly promotes macrophage mannose receptor (CD206) expression (122). This was stimulated by IL-13 and dependent on phospholipase A2 and peroxidation, implicating oxylipins. Furthermore, CD206 was induced by adding high amounts of the PPARγ ligand 15-deoxy-PGJ_2_, while IL-13 strongly induces *Alox15* in murine peritoneal macrophages (1). As further evidence, the induction of CD206 by IL-13 was demonstrated in a follow-up study (123).

Thus overall, the *Alox15^−/−^* fibrotic phenotype most likely results from a simple failure to generate abundant PPARγ ligand mixtures during the acute response to injury, as well as reduced basal PPARγ expression. In line with this, anti-inflammatory and pro-healing actions of pharmacological PPARγ agonists such as rosiglitazone in mouse woundγ models of diabetes and obesity have been described (124, 125). Sustained activity of NLRP3/NF-κB and TGF-β-pathway activity in *Alox15^−/−^* wounds at day 7 was seen. This is entirely consistent with PPARγ deficiency, with a large body of published work linking PPARγ, NLRP3 and TGF-β across multiple tissues and diseases (67–70, 87, 126–129). Indeed, directly supporting a mechanistic link between PPARγ and NLRP3 mediated by *Alox15^−/−^*, several 15-LOX metabolites suppress NLRP3 through activation of PPARγ (130). Separately, macrophage deletion of PPARγ prolongs inflammation and delays wound healing in mice (consistent with our data and using the same model we used) (67). There, prolonged accumulation of IL-1β was also seen, directly evidencing NLRP3 involvement.

As well as free oxylipins, eoxPL were detected, elevating significantly during remodeling, with 12-HETE-containing isomers reduced almost to basal levels in *Alox15^−/−^* wounds. eoxPL are also known as PPARγ ligands, although here, they did not restore MMP/collagen, most likely due to insufficient amounts (131, 132). Apart from very low levels of RvD5, SPM were not detected. A recent study on murine cutaneous wounds reported several RvDs, including RvDs1,2,3,4,5,6 and two 17*R*-isomers and proposed a central role for the lipids in repair using reconstitution studies with exogenous lipids (133). There, administration of individual RvDs at around 100 ng/wound/day demonstrated a pharmacological effect on healing (133). Oxylipin amounts administered here (6.5 ng total dose/wound/day) were considerably lower. Thus, in the previous study, the mechanism could involve SPM acting *via* low-affinity PPARγ binding and stimulating anti-inflammatory effects, consistent with other oxylipins. In this regard, the closely related RvD1 (7*S*,8*R*,17*S*-triHDOHE) was reported to activate PPARγ in a mouse model of acute lung injury (134). Alternatively, a recent study demonstrated that RvDs allosterically activate the PGE_2_ receptor, EP4, with RvD5 sensitizing at nM concentrations (34). In our study, 7,17-diHDOHE (RvD5) was detected at extremely low amounts (max amount 1.5 pg/wound, equating to around 4 fmol/wound). Although it is not possible to calculate local concentrations, these amounts appear too low to mediate EP4 sensitization or PPARγ activation.

PPARγ binds and is activated by many diverse lipid ligands with relatively low affinity and little differentiation of enantiomeric structure. Thus, the concerted action of many agonists generated in relatively high amounts during the healing process is consistent with the known role of PPARγ in mouse wound healing (67, 86). Here, our studies using *Alox15^−/−^* mice support the idea that abundant lipids generated by the 12/15-LOX pathway act in concert to promote the well-known anti-inflammatory actions of this transcription factor, preventing uncontrolled fibrosis through directly dampening NLRP3- and TGF-β-dependent inflammation. This is in line with the long-known action of many 12/15-LOX monohydroxy ligands as PPARγ ligands and previous reports of defective PPARγ signaling in *Alox15^−/−^* macrophages (13), and supports consideration of therapies targeting this pathway in defective wound healing in patients.

## Supplementary Material

Appendix 01 (PDF)

Dataset S01 (XLSX)

## Data Availability

RNASeq data have been deposited in BioStudies (E-MTAB-12845) (135).
