# Supplementary material for: 12/15-lipoxygenase orchestrates murine wound healing via PPARγ-activating oxylipins acting holistically to dampen inflammation
Source: Proc Natl Acad Sci U S A. 2025 Sep 4;122(36):e2502640122. doi: 10.1073/pnas.2502640122 (PMC12435204; doi:10.1073/pnas.2502640122)

Supplementary Methods and Data

Supplementary Methods

Animal model

Mice (8-12 weeks old C57/B6/J) were purchased from Charles River UK (Margate, UK), while *Alox15*$^{-/-}$ mice were bred in house (F11, C57BL/6J) in isolators. All animal experiments were performed in accordance with the United Kingdom Home Office Animals (Scientific Procedures) Act of 1986, under License (PPL 30/3334). Male and female mice were used for all studies except RNASeq where only males were used to reduce variation. Mice were housed in scantainers on a 12-hour light/dark cycle at 20 - 22 ºC, with free access to regular chow and water. Mice were anaesthetized using 3 - 3.5 % isoflurane delivered in 2 L per min 100 % oxygen. Once areflexic, mice received a sub-cutaneous dorsal injection of 10 µl Temgesic/Buprenorphine (1 µg). They were shaved and re-tested to confirm the areflexic state. Spinal midline was drawn before rotating to one side. Skin was folded using the spinal midline and two punch biopsy needles (BD pharma, UK) used to create 4 wounds (2 wounds per 4 mm biopsy needle)(1). Wounds were trimmed using clean scissors, and wounds photographed for size. Mice were transferred to a warming box until regaining consciousness, before transfer into cages lined with paper towels. Mice were monitored at 1 - 3 hours post wounding, and before the end of the light cycle. At 24 hours, mice were transferred into their original cages. In some experiments, mixtures of lipids were applied to wounds to determine their impact on healing. Two preparations were used, either “high-abundance” or “oxPL”. Here, immediately post-wounding, lipids were added (as described in Supplementary Table 1) in a final volume of either 50 µl (days 0,2) or 25 µl (days 4,6), with amount of lipids consistent across all days. Lipids were added in ethanol:Tween80:sterile water (1:1:18) as vehicle(2-4). On days 2, 4, 6, mice were briefly anesthetized as above to enable lipid application. Vehicle or lipids were added topically to the wound and allowed to sit in the wound surface for 15 min, before mice were allowed to recover consciousness. On various days post wounding, mice were euthanized and wounds collected (typical weights from 5-10 mg per wound) and either processed for histology as outlined below using

paraformaldehyde fixation or snap frozen in liquid nitrogen and then stored at -80 ^{0}C. For lipid supplementation studies, mice were euthanized at day 7.

Generation of histological tissue sections

At various time points up to 14 days, mice were killed using CO_2 (Schedule 1). The wound site and a small area of surrounding tissue was dissected and placed in 4 % paraformaldehyde for 72 hours, then 70% ethanol (to prevent further crosslinking), then the tissue processed for paraffin wax embedding. Wounds were placed into plastic cassettes and in a Leica tissue processor using the following parameters: ethanol (60 %) under ambient temperature under vacuum for 90 min with agitation. Ethanol (70 %) under ambient temperature under vacuum for 90 min with agitation. Six separate steps of ethanol (100 %) under ambient temperature under vacuum for 60 min with agitation. Xylene (100 %) at 37 °C under vacuum for 120 min with agitation. Xylene (100 %) at 45 °C under vacuum for 120 min with agitation. Two separate steps of wax (100 %) at 60 °C under vacuum for 120 min with agitation. Wax (100 %) at 60 °C under vacuum for 60 min with agitation. Wax (100 %) at 60 °C under vacuum for 45 min with agitation. Post-processing, plastic cassettes were removed, and wounds were cast side on in molten (60 °C) wax, which was allowed to harden to form tissue wax blocks. Wax blocks were fastened to a microtome stage and secured. 10 µm slices were taken from each revolution and transferred to a 40 ^{o}C water bath and allowed to unfold and float for 30 - 60 seconds before being placed onto a glass slide. Sections were left to dry at room temperature, then at 55 ^{o}C overnight**.**

DAB Immunohistochemistry (F480, LY6G)

Slides were placed in Histoclear (Fisher Scientific) for 3 min at room temperature then into ethanol at 100 %, 90 % and 70 % for 3 min each before being placed into running tap water for 5 min. Next, slides were dried with a paper towel, and a hydrophobic pen used to draw borders around each section. Antigen retrieval was performed by addition of 5µg/ml proteinase K in PBS for 7 mins at R.T. Slides were then rinsed in running tap water and H_2O_2 (3 %) was added and the sections incubated for 10 min at room temperature. Slides were washed in running tap water. Block solution (500 µl PBS + 1 % bovine serum albumin + 0.1 % fish gelatin + 0.5% Triton X-100) and avidin block (Avidin/Biotin block kit vector labs) were added and sections incubated for 30

min at room temperature. Block solution was removed, and slides washed twice with PBS. Primary antibody (Supplementary Table 2) or isotype controls made up in antibody solution (50 ml PBS + 1 % bovine serum albumin + 0.1% fish gelatin + 0.5% Triton X-100 and biotin block solution) was then added F4/80 Ab at 1/400 and Ly6G at 1/200. Slides with antibody solution were left for an extended period (14 hours or overnight) then washed with PBS 0.1% Tween 2 times for 5 mins. Biotin conjugated secondary antibody solutions were made up at 1:500 in block solution and added after the washes for 30mins. Secondary antibody was removed and sections washed 2 times as done before. Avidin-Biotin Complex (ABC) kit solution (Vector Labs) was then made up (as per manufacturers' instructions) 30 min before use, this was added for 30 min before being removed with one 5 minute wash. Sections were treated with a 3,3'Diaminobenzidine (DAB) solution (Vector Labs) for 1-2 min depending on staining intensity. The sections were washed in running tap water for 5 mins, then placed in hematoxylin for 15 secs. Sections were washed for 5 min in running tap water. Slides were then dipped 3 times in acid alcohol and then washed in running tap water for 1 min. Finally slides were placed in Scotts Tap water for 18 seconds, followed by a final wash in running tap water for 5 mins. Slides were then placed into increasing concentrations of ethanol (70 %, 90 %, 100 %) for 30 secs each, then placed into Histoclear for 2x 30 sec incubations. Sections were removed from Histoclear and dried carefully using paper towels. Slides were treated with 2 drops (50 μl) of distyrene and xylene (DPX solution) and covered with a glass cover slip and put in an oven at 60°C overnight.

DAB Immunohistochemistry (all other antigens).

Slides were placed in Histoclear (Fisher Scientific) for 2 min at room temperature then into ethanol at 100 %, 90 % and 70 % for 5 min each before being placed into DDH_2O for 5 min. Slides were then placed in citrate buffer (10 mM sodium citrate, 0.05 % Tween 20, pH 6.0) and incubated at 96 ^oC for 1 hr. They were cooled to room temperature over 30 min, then placed back into DDH_2O for 30 min. Next, slides were dried with a paper towel, and a hydrophobic pen used to draw borders around each section. Phosphate buffered saline (PBS, 1 ml) was added to sections for 2 min before removal using a Pasteur pipette. H_2O_2 (3 %) was added and the sections incubated for 10 min at room temperature. Slides were washed twice using PBS. Block solution

(500 µl PBS + 1 % bovine serum albumin + 0.1 % fish gelatin) and avidin block (Avidin/Biotin block kit vector labs) was added and sections incubated for 45 min at room temperature. Block solution was removed, and slides washed twice with PBS. Primary antibody (Supplementary Table 2) or isotype controls made up in antibody solution (50 ml PBS + 1 % bovine serum albumin + 0.1% fish gelatin and biotin block solution) was then added. Slides with antibody solution were left for an extended period (14 hours or overnight) then washed with PBS 5 times with 10-minute section submersion in between washes. Secondary (HRP conjugated) antibody solutions were made up at desired concentration (Supplementary Table 2) in block solution and added after the washes for two hours. Secondary antibody was removed and sections washed 5 times as done before with 10-minute gaps between wash steps, Avidin-Biotin Complex (ABC) kit solution (Vector Labs) was then made up (as per manufacturers' instructions) 15 min before use, this was added for 45 min before being removed with 5 washes. Sections were treated with a 3,3'Diaminobenzidine (DAB) solution (Vector Labs) for 5 - 7 min depending on staining intensity. The sections were washed multiple times with DDH_2O, then placed in hematoxylin for 2.5 minutes. Sections were washed for 5 min in DDH_2O. then placed into increasing concentrations of ethanol (70 %, 90 %, 100 %) for 2 min each, then placed into xylene for 20 sec. Sections were removed from xylene and dried carefully using paper towels. Slides were treated with 2 drops (50 µl) of distyrene and xylene (DPX solution) and covered with a glass cover slip and stored 24 hrs to dry.

Fluorescence Immunohistochemistry.

Slides were placed in Histoclear (Fisher Scientific) for 2 min at room temperature then into ethanol at 100 %, 90 % and 70 % made up in DDH_20 for 5 min each before being placed into DDH_2O for 5 min. Slides were placed in citrate buffer (10 mM sodium citrate, 0.05 % Tween 20, pH 6.0) and incubated at 96 ^oC for 1 hr. They were cooled to room temperature during 30 min, then placed into DDH_2O for 30 min. Next, slides were dried with a paper towel, and a hydrophobic pen used to draw borders around each section. PBS (1 ml) was added to sections for 2 min before removal using a Pasteur pipette. Block solution (500 µl PBS + 1 % bovine serum albumin + 0.1 % fish gelatin) was added and sections incubated for 45 min at room temperature. Block solution was removed and primary antibody (Supplementary Table 2) or isotype controls made up in antibody solution (50 ml PBS + 1 % bovine serum albumin + 0.1

% fish gelatin) were then added. Slides with antibody solution were left for an extended period (14 hours or overnight). The slides were then washed with PBS 5 times with 10-minute section submersion in between washes. Secondary antibody solutions were made up at 1:300 in block solution and added after the washes and left for 2 hrs at room temperature. Secondary antibody was removed and sections washed 5 times as done before with 10-minute gaps between wash steps. Sections were counterstained with DAPI solution (Invitrogen, 10ng/ml) and True Black (Cambridge BioSciences, Cambridge UK) as per manufacturer's instructions. Sections were washed 5 times in DDH_20 and then allowed to dry overnight and then coverslipped with ProLong™ Diamond Antifade mounting medium (Invitrogen).

Collagen staining using Masson trichrome

Collagenous fibres were visualized using Masson trichrome stain (Abcam, ab150686). Paraffin embedded sections were re-hydrated in decreasing concentration of ethanol (100 %, 96 %, 70 % in DDH_2O, then manufacturer's instructions followed: placing sections in Bouin's fluid, Weigert's iron hematoxylin solution, Biebrich scarlet/acid fuchsin solution, phosphomolybdic/phosphotungstic acid solution, aniline blue solution and acetic acid solution. Sections were dehydrated in increasing concentrations of ethanol (70 %, 96 %, 100 %), before being rinsed in xylene, dried and coverslipped with DPX mounting media. Sections were visualized under a light microscope Leica DM 2000 with a Leica DMC 2900 camera.

Image acquisition and analysis

Images were acquired with ether a light microscope (as detailed above) or epifluorescent microscope (EVOS M5000). Images were transferred to ImageJ software for post-acquisition analysis.

(i) For DAB images, contrast was enhanced by 5 % before isolation of the brown (DAB) staining channel after a color devolution set to 'H DDAB' (hematoxylin and DAB staining). Brown (dab staining) channels were then inverted and set to 'rainbow smooth' allowing localization and intensity of the DAB staining to be determined. Pixel count of the image was determined using a live histogram with data only from 70-255 (grey scale) to avoid background (hematoxylin) staining.

(ii) For fluorescent imaging pixel count or mean grey intensity (for Texas red or CY5 channels) was used. Isotype controls used are provided in Supplementary Figure 13.

RNASeq

Wound tissue dissected from 2 mice (8 wounds in total, 4 wounds per mouse) to generate one sample were snap-frozen in liquid N_2 before being stored at -80 ^{0}C. For WT and *Alox15*$^{-/-}$ a total of four samples were generated at each timepoint. Wounds were placed frozen into a pre-cooled pestle and mortar, then ground into a fine powder with liquid N_2 added. Tri reagent (1 ml, Sigma-Aldrich, UK) was added. The sample was then transferred to RNAse-free tubes and bromochloropropane (200 µl, Sigma-Aldrich, UK) added before vortexing. Samples were placed on ice for 5 min, before being centrifuged at 15,000 g for 15 min at 4 ^{o}C. The upper aqueous phase was transferred to a new tube and 250 µl of 3 M sodium acetate (pH 5.5), 700 µl (100 %) propanol and 10 µl glycogen added, before incubation at -80 ^{0}C overnight. Samples were centrifuged at 15,000 g for 15 min at 4^0 C to pellet the RNA. The pellet was washed 3 times using 70 % ethanol, then allowed to air dry for 5 min. RNAse-free water (50 µl) was added. Samples were cleaned using an RNeasy MinElute Cleanup Kit (Catalogue number 74204 Qiagen, MD, USA), using a column based clean up step. Sample (1 µl) was analyzed using a NanoDrop™ 2000/2000c Spectrophotometer (ThermoFisher Scientific, Newport UK), and to ensure samples were free of contamination. All samples had absorbance 260/230 ratios of between 1.7 and 2.0, and 260/280 values between 1.8 to 2.1. Each RNA sample (5ul) was used to determine RNA integrity analysis (RIN). Total RNA quality was assessed using the Agilent 4200 TapeStation with RNA ScreenTape® (Agilent Technologies) and quantity with the Invitrogen™ Qubit-iT™ RNA HS Assay Kit (Fisher Scientific) according to the manufacturer's instructions. Libraries were prepared from 300ng of total RNA with a RIN value > 7. Total RNA was depleted of ribosomal RNA and sequencing libraries prepared with the Illumina®TruSeq Stranded Total RNA Library Prep Gold (Illumina, Inc) kit using TruSeq CD Index Adapters1 (Illumina, Inc). The steps included the depletion of cytoplasmic and mitochondrial rRNA, depleted RNA fragmentation, 1st strand cDNA synthesis, 2nd strand cDNA synthesis, adenylation of 3' ends, adapter ligation, DNA fragment enrichment by PCR amplification (10-cycles) and validation.

The manufacturer's instructions were followed except for the cleanup after the ribozero depletion step where Ampure®XP beads (Beckman Coulter) and 80% Ethanol were used. The libraries were validated using the Agilent 4200 TapeStation® with high-sensitivity D1000 ScreenTape® (Agilent Technologies) to ascertain the insert size, and the Invitrogen™ Qubit™ dsDNA HS Assay Kit (Fisher Scientific) used to perform the fluorometric quantitation. Following validation, the libraries were normalized to 4 nM, pooled together and clustered on the cBot™2 (Illumina, Inc) following the manufacturer's recommendations. The pool was then sequenced using a 75-base paired-end (2x75bp PE) dual index read format on the HiSeq4000 (Illumina, Inc) according to the manufacturer's instructions. These combinatorial dual (CD) index adapters were formerly called TruSeq HT.

Lipid extraction

At time points < 14 days, mice were killed using CO_2 (Schedule 1). The wound site and a small area of surrounding tissue was dissected. Wounds were placed into 0.3 ml buffer containing 100 μM diethylenetriamine pentaacetate. Ceramic beads (15 – 20, 2.8 mm) were added (approx. 10/sample) and tubes placed into a bead homogenizer (Bead Ruptor Elite v1.1, Omni International). Wounds were homogenized using two 15 sec cycles (with a 30 sec delay) at 7.1 ms^{-1}, 4 ^oC. The tissue and beads were transferred into a glass extraction vial containing 1.25 ml hexane/isopropanol/glacial acetic acid (30:20:2). Tubes were rinsed with an additional 0.3 ml of buffer, then were vortexed and this was then added to the extraction vials. Internal standards (5 ng each of PC 14:0_14:0 and PE 14:0_14:0) and 5 ul of eicosanoid internal standards was added to each sample to give final concentration 75 nM (Supplementary Table 3). Following vortexing (1 min), hexane (1.25 ml) was added followed by vortexing, then centrifugation for 5 min at 1500 rpm, 4 °C. The upper organic phase was recovered into a clean tube. Hexane (1.25 ml) was added to the lower phase, which was again vortexed and centrifuged as above. The upper layer was combined with the previous hexane extract. A mixture of chloroform:methanol (1.9 ml, ratio 1:2) was added to the remaining aqueous phase. Following vortex, chloroform (0.625 ml) was added and samples again vortexed. HPLC grade water (0.625 ml) was added, and samples vortexed. Last, samples were centrifuged as above. The lower phase was carefully harvested and added to the hexane extracts obtained above.

Samples were dried using a RapidVap (RapidVap, Labconco®) then resolubilized in methanol (200 µl), with careful vortexing. The samples were split in half (2 x 100 µl samples, sample A and sample B), with half being stored for eoxPL analysis, and the remainder analyzed for oxylipins (see below). Samples were stored at -80 ºC until LC/MS/MS as described later.

Oxylipin extraction

100 µl methanol was added to sample B (listed above) followed by 1.655 ml HPLC-grade water. Glacial acetic acid (45 µl) was added to acidify and samples were mixed gently. Sep-Pak (C18 Waters) columns were loaded into a positive pressure (N_2) manifold, then pre-conditioned using 100 % MeOH (12 ml), followed by acidified water (6 ml with 0.4 % glacial acetic acid). Samples were loaded onto the preconditioned columns and allowed to drip through using gravity. Acidified water (10 ml) was passed through the column followed by 6 ml hexane (under nitrogen pressure). Columns were allowed to dry for 30 min. Oxylipins were eluted from the column using methyl formate (8 ml) into glass extraction tubes then solvent was evaporated under vacuum. Lipids were reconstituted using methanol (100 µl) and stored at -80 °C until LC/MS/MS as described below.

LC/MS/MS analysis of oxylipins

Lipids were quantified using reverse phase LC/MS/MS. They were separated using a gradient of 30 – 100 % B over 20 min (A: water:mobile phase B 95:5 + 0.1% acetic acid, B: acetonitrile:methanol, 80:15 + 0.1% acetic acid) on an Eclipse Plus C18 Column (Agilent), and analyzed on a Sciex QTRAP® 6500(5). Source conditions: TEM 475 °C, IS -4500, GS1 60, GS2 60, CUR 35. Chromatographic peaks were integrated using Multiquant 3.0.2 software (Sciex). The criteria for LOQ was signal:noise of at least 5:1 and with at least 5-6 points across a peak. Lipids were quantified using a standard curve generated and run at the same time as the samples and multiple reaction monitoring (MRM) channels and all assay parameters are provided in Supplementary Table 3 and(5). Each oxylipin was expressed per mg of skin tissue. Example chromatograms are provided in Supplementary Figure 14.

LC/MS/MS (reverse phase) analysis of oxidized phospholipids

Lipid extracts were separated using reverse-phase HPLC on a Luna 3 μm C18 150 × 2 mm column (Phenomenex, Torrance, CA) with a gradient of 50 – 100 % B over 10 min followed by 30 min at 100 % B (A: methanol:acetonitrile:water, 60:20:20 with 1 mM ammonium acetate, B: methanol, 1 mM ammonium acetate) with a flow rate of 200 μl min^{-1}. Lipids were analyzed in MRM mode on a 6500 Q-Trap (Sciex, Cheshire, United Kingdom), monitoring transitions from the precursor to product ion (dwell 75 ms) with TEM 500 °C, GS1 40, GS2 30, CUR 35, IS − 4500 V, DP − 50 V, EP − 10 V, CE − 38 V and CXP at − 11 V. The peak area was integrated and normalized to the internal standard. For quantification of HETE-PEs, standard curves were generated with PE 18:0a/5-HETE, PE 18:0a/8-HETE, PE 18:0a/11-HETE, PE 18:0a/12-HETE and PE 18:0a/15-HETE synthesized as described previously(6). Information on MRM transitions and *m/z* values are presented in Supplementary Table 5. HETE-PEs were quantified using standard curves with DMPE used as internal standard, with LOQ at signal:noise 5:1. Due to the limited standards available, identifications for some lipids are putative, based on the presence of characteristic precursor and product ions, and retention times. Example chromatograms are provided in Supplementary Figure 15.

Chiral analysis of oxylipins

Lipid extracts were separated using a Chiralpak IA-U column (50×3.0 mm, Diacel) in reverse phase mode, with flow rate 300 ml/min, at 40 °C, according to(7), on a 6500 Q-Trap (Sciex, Cheshire, United Kingdom). Mobile phase A was water:0.1 % acetic acid, and B was acetonitrile:0.1% acetic acid, and the gradient was 10 % B raised to 100 % B over 20 min followed by a 2 min hold then decrease to starting conditions over 2 min. MRM transitions and instrument parameters were as used for oxylipin reverse phase analysis.

Fatty acid analysis

Wounds were weighed, then homogenized with ceramic beads in 1 ml anti-oxidation buffer (100μM diethylenetriaminepentaacetic acid (DTPA), 100μM M butylated hydroxytoluene (BHT) in phosphate buffered saline) using a Bead Ruptor Elite for 2 x 30 second intervals at 6 m/s, under cooled nitrogen gas (4°C). Samples were spiked with 30 ng palmitic acid-d2 and stearic acid-d35. Lipids were extracted by adding a 2.5 ml solvent mixture (1 M acetic acid/isopropanol/hexane; 2:20:30, v/v/v) to 1 ml tissue homogenates in a glass extraction vial and vortexed for 30 sec. 2.5 ml hexane was

added to samples and after vortexing for 30 seconds, tubes were centrifuged (1500 rpm for 5 min at 4 °C) to recover lipids in the upper hexane layer (organic phase), which was transferred to a clean tube. Aqueous samples were re-extracted as above by addition of 2.5 ml hexane, and upper layers were combined. Lipid extraction from the lower aqueous layer was then completed according to the Bligh and Dyer technique using sequential additions of methanol, chloroform and water, and the lower layer recovered following centrifugation as above and combined with the upper layers from the first stage of extraction. Solvent was dried under vacuum and lipid extract reconstituted in 100 µl HPLC grade methanol. The lipid extract (100 µl) was derivatized using 3-nitrophenylhydrazine, by adding 50 µl 200 mM 3-nitrophenyl-hydrazine (50:50, methanol,H_2O) and 50 µl 120 mM N-ethyl-N'-(3-dimethylaminopropyl) carbodiimide hydrochloride and 6 % pyridine (50:50, methanol:H_2O). Samples were vortexed and incubated for 30 min at 40°C. Excess derivatization reagents were quenched by the addition of 0.5 % formic acid (100 µl; 75:25, methanol:H_2O) and incubation at 40 °C for 30 min. Samples were aliquoted into HPLC vials for LC-MS/MS. For every sample batch, six blanks of antioxidant buffer were extracted and derivatized to account for background levels of lipids. LC–MS/MS analyses was performed on a Nexera liquid chromatography system (Shimadzu) coupled to a QTRAP 6500 mass spectrometer with an ESI source (AB Sciex) in multiple reaction monitoring (MRM) mode. Lipid separation was achieved using a Kinetex Polar C18 reverse phase column (100 Å, 100 × 2.1 mm, 2.6 µm particle size; Phenomenex) and gradient elution of two mobile phases (A: 100% H_2O + 0.1% formic acid, B: 100% methanol + 0.1% formic acid) at 0.2 ml/min with injection volume 1 µl; column temperature 50° C. Source settings CUR 20, IS -4500, TEM 400, GS1 25, GS2 25. Fatty acids were detected in negative ion mode. Calibration curves were run alongside sample analyses. Peak areas were integrated using MultiQuant software (AB Sciex). Lipids concentrations were derived from comparisons with an external calibration curve of the ratio of the lipid primary standard to a deuterated internal standard. Limit of quantitation used signal:noise of >5:1 and at least 6-7 data points per peak, while ensuring that all peaks were within the linear dynamic range of the assay.

Gel zymography for MMP activity

On day 7, wounds were harvested, snap frozen in liquid N_2 and stored at -80 ^{O}C until processing. Tissue was homogenized using ceramic beads in a Bead Ruptor Elite v1.1 (Omni International) using two rounds at 8 m/sec for 15 sec with a 30 sec dwell time, in 0.3 ml ice cold lysis buffer with protease inhibitors (50 mm Tris-HCl, 150 mm NaCl, 1 % Nonidet P-40, 0.1 % SDS, 0.1 % deoxycholic acid, 2 µg/ml leupeptin, 2 µg/ml aprotinin, 1 mm PMSF pH 7.4). Samples were placed on a rotary carousel for 30 min at 4 ^{o}C. The homogenate was centrifuged at 15,000 × g for 5 min at 4°C. Protein was measured using the Bradford assay (Thermo fisher) and samples diluted to 15 µg/sample. Samples were diluted in sample buffer (Zymogram Sample Buffer, #1610764, Biorad) and loaded into the wells of precast gels (Novex™ 10% Zymogram Plus (Gelatin)) gels (Thermo Fisher)). Electrophoresis was performed with a Tris-glycine running buffer (LC-26754, Invitrogen), at 125 V for 140 min. The gel was incubated for 1 hr at room temperature in 3 % Triton X-100 on a rotary shaker, then incubated with development buffer (50 mm Tris base, 40 mm HCl, 200 mm NaCl, 5 mm $CaCl_2$, and 0.2 % Brij 35) at 37 °C for 18 – 30 hr (depending on experiment) on a rotary shaker. Gels were stained using 0.5 % w/v Coomassie blue G-250 in 50 % DDH_20, 40 % methanol and 10 % acetic acid for 2 hr, and then destained for 1 hr using diluent (50 % DDH_20, 30 % methanol, 10 % acetic acid). Gelatinolytic activity was observed as clear zones or bands at the appropriate molecular weights. Mouse MMP-9 and human MMP-2 (R&D Systems) were used to locate bands. Bands were quantified using the gel analysis plugin on ImageJ.

Cell transfection and reporter assays.

HEK293 cells were cultured and transfected as described previously with plasmids expressing mouse PPARγ or PPARγ together with RXRα and the *Firefly* luciferase under the control of 3x Ppar Responsive Element (PPRE)(8). The *Renilla* luciferase plasmid pRL-TK (Promega) was also included in the transfection as an internal control. At day 2, the cells underwent 24 h incubation in 50 µl media, with 1 µM rosiglitazone or DMSO (vehicle), lipid mixtures or methanol (vehicle), 1 µM 9-cis-retinoic Acid (RA) with or without 1 µM rosiglitazone or DMSO (vehicle, the same as for rosiglitazone) and 10 µM 17(S)-HDHDA or ethanol (vehicle) with or without 1µM 9-Cis-RA. For each experiment (day) there were 4-6 replicates per condition and the experiment was repeated 3 independent times to give n = 3. On day 3, lysates were

prepared, and a luciferase assay was performed using a Dual-Luciferase Reporter Assay System (Promega)

Statistical Analysis

To compare wounds using immunohistochemistry, Students T-test was used * $p<0.05$, ** $p<0.01$, *** $p<0.001$ and **** $p<0.0001$. For multi-time point analysis, data were analyzed using one-way ANOVA, with $p < 0.05$ considered statistically significant. For RNASeq, paired-end reads from Illumina sequencing were trimmed with Trimmomatic and assessed for quality using FastQC with default parameters. Reads were mapped to the Mouse GRCm38 reference genome using STAR and counts were assigned to transcripts using FeatureCounts with the GRCm38.84 Ensembl gene build GTF. Both the reference genome and GTF were downloaded from the Ensembl FTP site(8-13). Differential gene expression analyses used the DESeq2 package (13) to produce an excel output listing adjusted p-value and log2 fold change between conditions. The data were then filtered so that only genes with adjusted p-value < 0.05 were taken forward for analysis (Benjamini-Hochberg adjustment). Downstream pathway analyses and gene annotation were performed in ingenuity IPA (Qiagen IPA). Cytoscape was used to cluster genes by expression (FPKM) over all samples and timepoints(14). Pearson correlation coefficients were calculated for all possible gene pairs, and only highly significant genes retained ($|r| > 0.8$). During analysis, one wild-type (baseline) RNASeq sample failed a quality control check (*Alox15* expression data indicated it was a knockout) and was removed from further analysis. For temporal analysis of gene expression changes, lists of genes were generated using MATLAB_R2022a. A heatmap was generated using Clustergram in MATLAB, where data was standardized for each gene, so that the mean is 0 and the standard deviation is 1, and hierarchical clustering for rows performed. Data were analyzed using Ingenuity Pathway Analysis. For oxylipidomics, a two-way ANOVA was used to calculate differences within groups, ($p < 0.05$ considered significant), with Bonferroni post hoc test (https://statisty.app/two-way-anova-calculator). Heatmaps for oxylipins and oxPL were generated using an R script that processes the raw dataset, into log10 values, based on an average of 5 biological replicates, which then passes the resulting data to pheatmap (https://cran.r-project.org/web/packages/Pheatmap) to produce the final image. Box and whisker shows median with the ends of the whiskers set at

1.5*IQR above the third quartile (Q3) and 1.5*IQR below the first quartile (Q1). If the Minimum or Maximum values are outside this range, then they are shown as outliers.

Supplementary Results.

Structural analysis of resolvinD5.

While most SPM were not detected, a peak co-eluting with the resolvinD5 (RvD5, 7*S*,17*S*-diHDOHE) standard was seen (Supplementary Figure 14). RvD5 represents one stereoisomer of 4 possible 7,17-diHDOHEs. The lipid is described to originate from 12/15-LOX dependent formation of 17-HDOHE, followed by its further oxygenation by 5-LOX, following transcellular uptake of 17-HDOHE into leukocytes(15). However reverse phase LC/MS/MS is unable to fully separate these isomers, and it was not possible to obtain an MS/MS spectrum to compare with the standard, due to the low levels of the lipid present in wounds. To address this, secondary MRMs were next analyzed, with two arising from fragmentation at C7 (m/z 141, 199) and one at C17 (m/z 261, Supplementary Figure 16 A-D). For the synthetic RvD5, the three MRMs co-eluted at 10.06 min as expected (Supplementary Figure 16 C). In day 1 wound extracts, a lipid was detected at 10.06 min showing co-eluting ions for m/z 199 and 141, however the third MRM (m/z 261) eluted slightly earlier (Supplementary Figure 16 B,D). Both the putative RvD5, and other later eluting lipids that were detected using these MRMs were absent from *Alox15*$^{-/-}$ wound extracts (Supplementary Figure 16 E). Next, chiral analysis was undertaken, with synthetic RvD5 eluting at 7.23 min, with the expected MRMs also co-eluting (Supplementary Figure 16 F). Chiral analysis of the wound lipid extract showed several peaks eluting between 6.8 – 8 min (Supplementary Figure 16 G). Based on ion ratios, the large peak at 7.89 min is likely to be the same as the two seen around 11 min on reverse phase LC/MS/MS (Supplementary Figure 16 A,G). A very small peak at 7.22 min had the same retention time as RvD5 standard (Supplementary Figure 16 F,G) and similar ion ratios with the m/z 199 ion dominating. A recent study using a similar chiral separation method monitored RvD5 and its isomers after pre-isolating 7,17-diHDOHE, using the MRM m/z 359-141, and showed that RvD5 elutes slightly later than its isomers, 7*R*,17S, 7*R*,17*R* and 7*S*,17*R*-diHDOHEs(16). In mouse wounds earlier eluting peaks that could represent these isomers are seen. These have the same MRMs as RvD5, in particular the peak at 6.84 min (Supplementary Figure 16 G, inset)

indicating oxygenation at C7 and C17. Several other lipids eluted just after the putative RvD5 (7.3-7.6 min), and these may represent additional related structures such as positional isomers (Supplementary Figure 16 G). Spiking the wound lipid extract with synthetic standard showed co-elution of RvD5 with the peak at 7.2 min (Supplementary Figure 16 H,I). Overall, the data suggest the wound may contain low levels of RvD5, together with other related isomers of 7,17-diHDOHE. As for reverse phase analysis, the putative RvD5 peak along with all the other lipids detected using chiral analysis were absent in *Alox15*$^{-/-}$ wounds indicating their dependence on the enzyme (Supplementary Figure 16 J). However, 7,17-diHDOHE (coeluting with RvD5) was present at ~0.5 % the levels of 17-HDOHE. Considering this, and the presence of isomers, it is possible RvD5 may have originated from non-enzymatic secondary oxidation of 12/15-LOX-derived 17*S*-HDOHE to form 7*R*,17*S*-diHDOHE and 7*S*,17*S*-diHDOHE(RvD5) eluting at 6.84 and 7.22 min, respectively. Further studies are needed to establish the origin of the lipid in wounds, for example pharmacological/genetic inhibition of 5-LOX, and MS/MS analysis of the 7*R*,17*S*-diHDOHE epimer under our chromatographic conditions.

Comparison of temporal changes in gene expression suggest additional transcription activators regulated by Alox15 beyond PPARg include elf4, Cebpb and Tcf3.

To further interrogate *Alox15*$^{-/-}$ wounds for transcriptional regulators beyond PPARγ, a temporal analysis was performed on the RNASeq data. Here, analysis of individual strains separately allowed testing for genes behaving differently during progression of wound healing. In WT mice, 1705 transcripts significantly increased > 50 % on Days 0 and 4, while by Day 7, they reduced by > 25 % compared to Day 4 (Supplementary Figure 17 A, List 1). Thus, these elevate on acute injury, then return close to normal after one week (Supplementary Table 10). Interrogating these in *Alox15*$^{-/-}$ mice, 154 did not increase on Day 4 by > 25 % compared to Day 0 (Supplementary Figure 17 B, List 2, Supplementary Table 11). Thus, these failed to elevate during acute inflammation in *Alox15*$^{-/-}$. Using IPA analysis, several transcription factors were identified as possible upstream regulators. *Elf4* is a known anti-inflammatory transcription regulator of inflammation, which targets several genes in the list, including *Anln, Asf1b, Ccnb2, Cdca3, Cenpa, Cenpe, Cks2, E2f8, Hmmr, Kif4a, Mcm10, Ndc80, Oip5, Rrm2, Tpx2(17)*. In support of this idea, we found that

expression of *Elf4* was significantly increased by wounding in both WT and *Alox15*$^{-/-}$ mice (Supplementary Figure 17 D). This indicates that while *Elf4* is upregulated by wounding, it may not be transcriptionally active in the absence of 12/15-LOX. Additional transcription regulators strongly associated with the initial response to wounding included *Tcf3* and *Cebpb*. *Tcf3* promotes cell migration and wound repair (18), and *Cebpb* is involved in macrophage repair responses and inflammation (19, 20). Expression data for these genes showed *Cebpb* is upregulated on Day 4, significantly in *Alox15*$^{-/-}$, while reduced back to baseline expression by Day 7. However, *Tcf3* expression was unaffected by wounding in either strain (Supplementary Figure 18 A,B).

Last, genes in List 1 were re-interrogated to identify transcripts that reduced < 25 % on Day 7 compared to Day 4 in *Alox15*$^{-/-}$. These represent genes that fail to resolve to basal levels during inflammation in *Alox15*$^{-/-}$. Here, 538 were identified (Supplementary Figure 18 C, Supplementary Table 12, List 3). IPA analysis of these proposed "lipopolysaccharide" as the top upstream regulator, consistent with the failure of many known pro-inflammatory genes which respond to this bacterial product to reduce back to basal levels as shown in our earlier analysis, e.g., *Il6*, *Ptgs2*, and *Il1b* (Figure 5). Similarly, IPA also proposed the top affected canonical pathway for List 3 as "Pathogen Induced Cytokine Storm Signaling" which includes 33 genes which failed to fully resolve. These are shown in a heatmap, comparing Days 4 or 7 with Day 0 in both WT and *Alox15*$^{-/-}$ mice. Three distinct groups are seen (Supplementary Figure 18 C):

(i) Genes that elevate by Day 4 and then are reduced by Day 7 in WT. They elevate to a similar level in *Alox15*$^{-/-}$ but do not fall back to baseline by Day 7 or elevate further by that time (*Fos, Ddx58, Stat1, Ccr3, Nlrc4, Naip1, Faslg, Gsdmd, Clec7a, Itb, Lif, Stat4)*

(ii) Genes that elevate by Day 4 then reduce by Day 7 in WT, while elevating higher in *Alox15*$^{-/-}$ at Day 4, and not falling back to baseline by Day 7 (*Cxcl3, Aim2, Ccl4, Ccl3l3, Cxcl10, Pgf, Nos2, Cxcl2, Tlr2, Mlkl, Sting1, Cxcr4, Csf2rb, Zbp1, Cklf*)

(iii) Genes that elevate far higher in WT than *Alox15*$^{-/-}$ but reduce back by Day 7 in both (*Il1r1, Ccl7, Cxcl6, Col13a1, Ccr5, Irf7*).

Analysis using STRING 11.5 showed that group (i) genes are members of networks that regulate cytokines, including IFN-I, IL-12, IL-21, IL-35, IL-20 family and IL-6 family

signaling networks. For groups (ii) and (iii) the main KEGG pathways were “cytosolic DNA-sensing” and “IL-17 signaling”, respectively. Notably this analysis confirms our earlier data which indicates that inflammatory signaling is strongly impacted by *Alox15$^{-/-}$* deletion, while identifying a large number of novel targets for further study.

Supplementary Figure Legends

Supplementary Figure 1. Mouse wounds show significant elevation of IL-4 and IL-13 receptor proteins. Gene expression for components of the IL-4 and IL-13 signaling pathway was measured using RNASeq as outlined in methods (n = 3 – 4 wounds per group). For all gene expression data, students t-test, followed by Benjamin Hochberg correction: * p <0.05, ** p < 0.01, *** p < 0.005.

Supplementary Figure 2. *Alox15$^{-/-}$* wounds show reduced wound bed macrophages, and increased smooth muscle actin, SSEA3 and Ki-67 expression. *Panel A. Alox15 deletion leads to reduced macrophage influx*. F480+ve cells were measured in wounds as described in Methods (n= 6–8/group, 4-9 fields per wound). *Panel B. Representative images from Panel A*. *Panel C. Neutrophil cell numbers (visualized with by Ly6g DAB positive staining) are similar in wildtype and Alox15$^{-/-}$ mice*, (n = 7–8/group, 4-9 fields per wound). *Panel D. The myofibroblast marker smooth muscle actin alpha was increased in Alox15$^{-/-}$ wounds*. Cells were stained with anti- alpha smooth muscle actin and visualized with DAB staining (n = 7-8/group). For panels B,H: unpaired t-test, * p < 0.05, *** p<0.005. *Panel E. Representative images of smooth muscle actin. Panel F. SSEA3 and Ki-67 are elevated in SSEA3 and Ki-67 Alox15$^{-/-}$ wounds***.** Expression was measured using immunohistochemistry, followed by DAB visualization. n = 6/group (SSEA3), 10/group (Ki67). *Panels G,H. Representative data from Panel F*.

Supplementary Figure 3. Epithelial proliferation and terminal differentiation of keratinocytes are not impacted in *Alox15$^{-/-}$* either basally or post-wounding*,* and IL-6 is unaffected*.* *Panel A. Cytokeratin 14 migration migrated from the wound edge in Alox15$^{-/-}$ wounds at day 4 is not impacted.* Quantification of the migratory distance

of highly proliferative non-differentiated cytokeratin 14 (green) and non-proliferative terminally differentiated cytokeratin 10 (pink) was quantified using fluorescence immunohistochemistry. n = 4/group. *Panel E. Representative images from Panel B. Panel C. Representative images showing cytokeratin 10 and 14 staining in non-wounded skin*. All panels, unpaired Students t-test, * p<0.05, *** p, 0.005. *Panel D. Alox15$^{-/-}$ wounds show unchanged IL-6 expression*. IL-6 was measured using fluorescence immunohistochemistry. n = 6/group.

Supplementary Figure 4. Heatmap and time course data for oxylipin levels during wounding shows that *Alox15$^{-/-}$* wounds generate lower levels of many oxylipins. *Panel A. A heatmap shows log10 of mean values for all lipids across all groups tested.* Oxylipins were measured using LC/MS/MS as outlined in Methods (n = 5 samples/time point, with 4 wounds pooled/sample). *Panel. Oxylipins are rapidly elevated post-wounding, but many are reduced in Alox15$^{-/-}$ wounds.* Oxylipins were measured using LC/MS/MS as outlined in Methods. n = 6 samples/time point, with 4 wounds pooled/sample. For all panels differences between groups were analyzed using two-way Anova (red stars), with Bonferroni post hoc test between individual time points (black stars), mean ± SEM, * p < 0.05, ** p<0.01, *** p < 0.005.

Supplementary Figure 5. Time course data for oxylipins generated during wounding, as in Supplementary Figure 2. Oxylipins were measured using LC/MS/MS as outlined in Methods. n = 5 samples/time point, with 4 wounds pooled/sample. For all panels differences between groups were analyzed using two-way Anova (red stars), with Bonferroni post hoc test between individual time points (black stars), mean ± SEM, * p < 0.05, ** p<0.01, *** p < 0.005.

Supplementary Figure 6. Heatmap of eoxPL generation during wounding, and individual timecourses of 12-HETE-PEs. *Panel A.* Heatmap shows expression (log10) of mean values for all lipids across all groups tested eoxPL were measured using LC/MS/MS as outlined in Methods. n = 5 samples/time point, with 4 wounds pooled/sample. *Panel B. 12-HETE-PE isomers are significantly reduced in Alox15$^{-/-}$ wounds.* Oxidized phospholipids were measured using LC/MS/MS as outlined in Methods (n = 5 samples/time point, with 4 wounds pooled/sample). Unpaired t-test, * p <0.05, ** p < 0.01, *** p < 0.005.

Supplementary Figure 7. Raw data for MMP quantitation in wounds *Panels A,B.* Gel zymography showing data used to quantify MMP activities during wounding.

Supplementary Figure 8. Mouse wounds show no elevation of PPRE-inducible genes following wounding. Gene expression was measured using RNASeq as outlined in methods (n = 3 – 4 wounds per group).

Supplementary Figure 9. Genes in the network show the same pattern of expression throughout the time course, increasing on Day 4, but with a failure to revert to baseline at Day 7 in *Alox15*$^{-/-}$ wounds. Data for several affected genes are shown, all gene expression data was normalized to its Day 0 mean value, and then expressed as fold-change (n = 3 – 4 per group). For all gene expression data, students t-test, followed by Benjamin Hochberg correction: * $p < 0.05$, ** $p < 0.01$, *** $p < 0.005$.

Supplementary Figure 10. Mouse wounds show significant increases in TGFβ signaling genes following wounding but miR-21 doesn't resolve at day 7. Gene expression was measured using RNASeq as outlined in methods (n = 3 – 4 wounds per group). For all gene expression data, students t-test, followed by Benjamin Hochberg correction: * $p < 0.05$, *** $p < 0.005$.

Supplementary Figure 11. High oxylipins activate PPARγ transcription while most PPARγ co-factors are expressed but only Helz2 is significantly increased by wounding. *Panel A. Oxylipin activation of PPARγ transcription.* Oxylipins, Rosiglitazone or vehicle controls were added to HEK293 cells expressing mouse PPRE, as described in Methods. After 24 hrs, luciferase activity was analyzed. Data are shown normalized to the relevant vehicle control (n = 3 independent experiments, mean +/- SEM, students t-test, * $p < 0.05$, *** $p < 0.005$. *Panel B. Co-expression of RXR doesn't sensitize PPARγ to 17-HDOHE*. 17-HDOHE (10 μM), Rosiglitazone, 9-cis retinoic acid (1 μM) or vehicle controls were added to HEK293 cells expressing mouse PPRE, with/without RXR, as described in Methods. After 24 hrs, luciferase

activity was analyzed. Data are shown normalized to the relevant vehicle control (n = 3 independent experiments, mean +/- SEM, students t-test. *Panel C. Expression of PPARγ co-activators during wounding.* Gene expression for protein-coactivators was measured using RNASeq as outlined in methods (n = 3 – 4 wounds per group).

Supplementary Figure 12. *Alox15*-deletion doesn't alter expression of LXR or Nrf2-dependent genes during wounding Gene expression was measured using RNASeq as outlined in methods (n = 3 – 4 wounds per group). For all gene expression data, students t-test, followed by Benjamin Hochberg correction: * $p < 0.05$, ** $p < 0.01$, *** $p < 0.005$.

Supplementary Figure 13. Isotype and no-primary controls for antibodies in the study. Left panels: To confirm target-specific DAB staining both a no primary control and isotype control were run for the antibodies F4/80, INFγ and CD206 followed by hematoxylin counter staining. Images were taken at 20x magnification, scale bar =100µm. Right panels: To confirm target specific immunofluorescence staining both a no-primary control and isotype control was run for the antibodies, Cytokeratin 10 (K10), cytokeratin 14 (K14), IL6, Phospho SMAD3, Phospho STAT3, followed by a DAPI stain. Images were taken at 10x magnification, scale bar =200µm.

Supplementary Figure 14. Representative chromatographic peaks for oxylipins detected during wounding. Screenshots were taken from Multiquant software, with the shaded area indicating the peak integrated. Wound lipids were confirmed to co-elute with primary standards in the same analytical batch.

Supplementary Figure 15. Representative chromatographic peaks for eoxPL generated during wounding. Identity was verified comparing retention time with standards as outlined in (6), based on comparison with PE 18:0a_HETE for the relevant positional isomers. Note that standards for 16:0p, 18:0p and 18:1 forms are not available, and so relative RT compared to standards is used along with MRM transitions which use internal daughter ions for all HETE positional isomers, along with LOQ of > 5 for signal:noise for peaks. The order of elution is characteristic for the different sn1 forms, as shown in (21-23).

Supplementary Figure 16. Reverse phase and chiral phase analysis of 7,17-diHDOHE suggests RvD5 along with additional isomers are present in WT mouse wounds at day 1. Lipid extracts from Day 1 wounds were pooled and analyzed using reverse and chiral phase LC/MS/MS as described in Methods. For reverse phase, the same method was used as for the oxylipin assay, but focusing on MRM transitions for RvD5, and removing scheduling. *Panels A-E. Reverse phase analysis of the synthetic RvD5 standard along with wound lipid extract from WT and Alox15$^{-/-}$ mice.* Panels A,B,D show WT lipid extract, while Panel C shows the RvD5 standard. *Panels F-J. Chiral phase analysis of synthetic RvD5 standard along with wound lipid extract from WT and Alox15$^{-/-}$ mice.* Panels G-J show wound extracts, while F shows the RvD5 standard.

Supplementary Figure 17. IPA temporal analysis shows altered regulation of several inflammatory pathways and induction of *Elf4*. *Panel A. List 1 represents 1705 transcripts in the WT condition* whose expression on Day 4 is significantly increased by > 50% compared to Day 0, i.e., with log2-fold change at least log2FC (1.5) and adjusted p-value < 0.05, but where log2FC values on Day 7 compared to Day 4 are reduced by > 25 %. *Panel B. List 2 represents a subset of List 1 consisting of 154 transcripts*, whose expression on Day 4 is not increased by > 25% compared to D0 in *Alox15*$^{-/-}$ wounds, i.e., with a maximum log2-fold change log2FC (1.25). Wilcoxon signed-rank test shows that the D4 data between the WT and *Alox15*$^{-/-}$ is significantly different. *Panel C.* List 3 comprises 538 transcripts from List 1 with a minimum log2-fold change log2FC (1.5) and adjusted p-value < 0.05 on D4 in *Alox15*$^{-/-}$ wounds, whose log2FC values are reduced by < 25 % on Day 7 compared to Day 4 in *Alox15*$^{-/-}$ wounds. Wilcoxon signed-rank test shows that the D7 data between the WT and *Alox15*$^{-/-}$ conditions is significantly different. *Panel D. Elf4 is induced on wounding.* Gene expression data was normalized to its Day 0 mean value, and then expressed as fold-change (n = 3 – 4 per group). For all gene expression data, students t-test, followed by Benjamin Hochberg correction: * p <0.05, ** p < 0.01, *** p < 0.005.

Supplementary Figure 18. *Cebpb* is upregulated during wounding, but not Tcf3, while IPA identifies groups of genes with common behaviour that don't resolve fully post wounding. *Panels A-C. Data from gene expression is shown for WT and*

Alox15$^{-/-}$ *wounds during the time course (n = 3 – 4 per group).* For all gene expression data, students t-test, followed by Benjamin Hochberg correction: * $p < 0.05$, ** $p < 0.01$, *** $p < 0.005$. *Panel C. IPA analysis shows clusters of genes with similar behaviour, which don't fully resolve.* Genes that do not fully resolve but behave in groups are plotted in this heatmap. Plotted are log2fold change data for wounds of the same strain comparing day 0 with either day 4 or day 7, within strain comparisons only.

REFERENCES

1. A. V. Kostarnoy *et al.*, Receptor Mincle promotes skin allergies and is capable of recognizing cholesterol sulfate. *Proceedings of the National Academy of Sciences of the United States of America* **114**, E2758-E2765 (2017).
2. C. A. Castro, J. B. Hogan, K. A. Benson, C. W. Shehata, M. R. Landauer, Behavioral effects of vehicles: DMSO, ethanol, Tween-20, Tween-80, and emulphor-620. *Pharmacol Biochem Behav* **50**, 521-526 (1995).
3. J. L. Wiley, J. J. Burston, Sex differences in Delta(9)-tetrahydrocannabinol metabolism and in vivo pharmacology following acute and repeated dosing in adolescent rats. *Neurosci Lett* **576**, 51-55 (2014).
4. R. Elfiyani, A. Amalia, S. Pratama, Effect of Using the Combination of Tween 80 and Ethanol on the Forming and Physical Stability of Microemulsion of Eucalyptus Oil as Antibacterial. *Journal of Young Pharmacists* **9**, s1-s4 (2017).
5. M. Misheva *et al.*, Oxylipin metabolism is controlled by mitochondrial beta-oxidation during bacterial inflammation. *Nat Commun* **13**, 139 (2022).
6. A. H. Morgan *et al.*, Quantitative assays for esterified oxylipins generated by immune cells. *Nat Protoc* **5**, 1919-1931 (2010).
7. X. Fu, Z. Xu, M. Gawaz, M. Lämmerhofer, UHPLC-MS/MS method for chiral separation of 3-hydroxy fatty acids on amylose-based chiral stationary phase and its application for the enantioselective analysis in plasma and platelets. *Journal of Pharmaceutical and Biomedical Analysis* **223**, 115151 (2023).
8. A. M. Bolger, M. Lohse, B. Usadel, Trimmomatic: a flexible trimmer for Illumina sequence data. *Bioinformatics* **30**, 2114-2120 (2014).
9. A. Dobin *et al.*, STAR: ultrafast universal RNA-seq aligner. *Bioinformatics* **29**, 15-21 (2013).
10. Y. Liao, G. K. Smyth, W. Shi, featureCounts: an efficient general purpose program for assigning sequence reads to genomic features. *Bioinformatics* **30**, 923-930 (2014).
11. https://www.bioinformatics.babraham.ac.uk/projects/fastqc/ (
12. http://www.ensembl.org/info/data/ftp/index.html/ (
13. M. I. Love, W. Huber, S. Anders, Moderated estimation of fold change and dispersion for RNA-seq data with DESeq2. *Genome Biol* **15**, 550 (2014).
14. P. Shannon *et al.*, Cytoscape: a software environment for integrated models of biomolecular interaction networks. *Genome Res* **13**, 2498-2504 (2003).
15. S. Hong, K. Gronert, P. R. Devchand, R.-L. Moussignac, C. N. Serhan, Novel Docosatrienes and 17S-Resolvins Generated from Docosahexaenoic Acid

in Murine Brain, Human Blood, and Glial Cells: AUTACOIDS IN ANTI-INFLAMMATION *. *Journal of Biological Chemistry* **278**, 14677-14687 (2003).
16. K. R. Kampschulte N, Loewen A, Schebb NH, Deducing formation routes of oxylipins by quantitative multiple heart-cutting achiral-chiral 2D-LC-MS. *ChemRxiv* 10.26434/chemrxiv-2024-tgv9t-v3 (2024).
17. P. M. Tyler *et al.*, Human autoinflammatory disease reveals ELF4 as a transcriptional regulator of inflammation. *Nature Immunology* **22**, 1118-1126 (2021).
18. Q. Miao *et al.*, Tcf3 promotes cell migration and wound repair through regulation of lipocalin 2. *Nature Communications* **5**, 4088 (2014).
19. C. Y. Ko, W. C. Chang, J. M. Wang, Biological roles of CCAAT/Enhancer-binding protein delta during inflammation. *J Biomed Sci* **22**, 6 (2015).
20. D. Ruffell *et al.*, A CREB-C/EBPbeta cascade induces M2 macrophage-specific gene expression and promotes muscle injury repair. *Proc Natl Acad Sci U S A* **106**, 17475-17480 (2009).
21. S. R. Clark *et al.*, Esterified eicosanoids are acutely generated by 5-lipoxygenase in primary human neutrophils and in human and murine infection. *Blood* **117**, 2033-2043 (2011).
22. B. H. Maskrey *et al.*, Activated platelets and monocytes generate four hydroxyphosphatidylethanolamines via lipoxygenase. *J Biol Chem* **282**, 20151-20163 (2007).
23. C. P. Thomas *et al.*, Phospholipid-esterified eicosanoids are generated in agonist-activated human platelets and enhance tissue factor-dependent thrombin generation. *J Biol Chem* **285**, 6891-6903 (2010).

Supplementary Figure 1

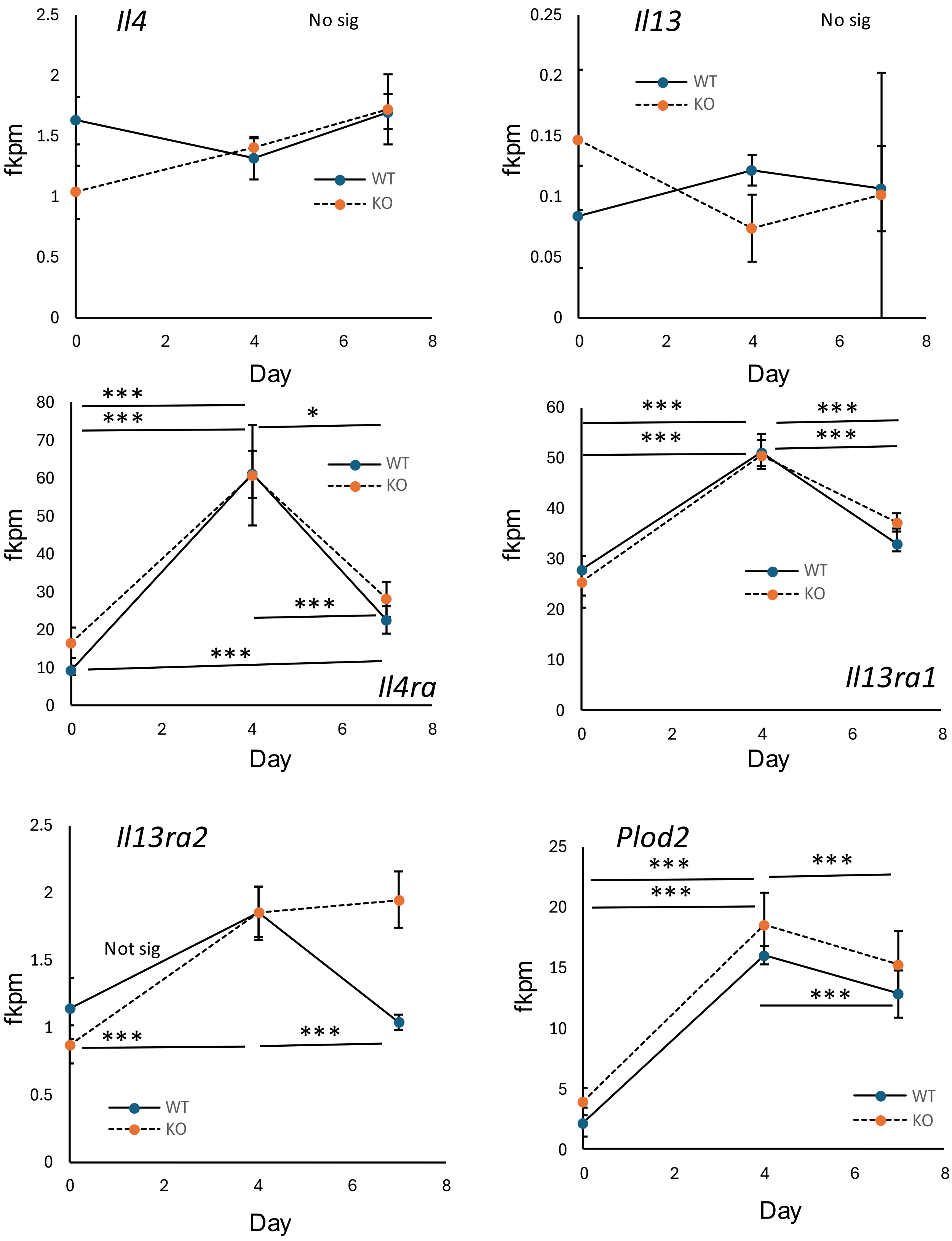

Supplementary Figure 2

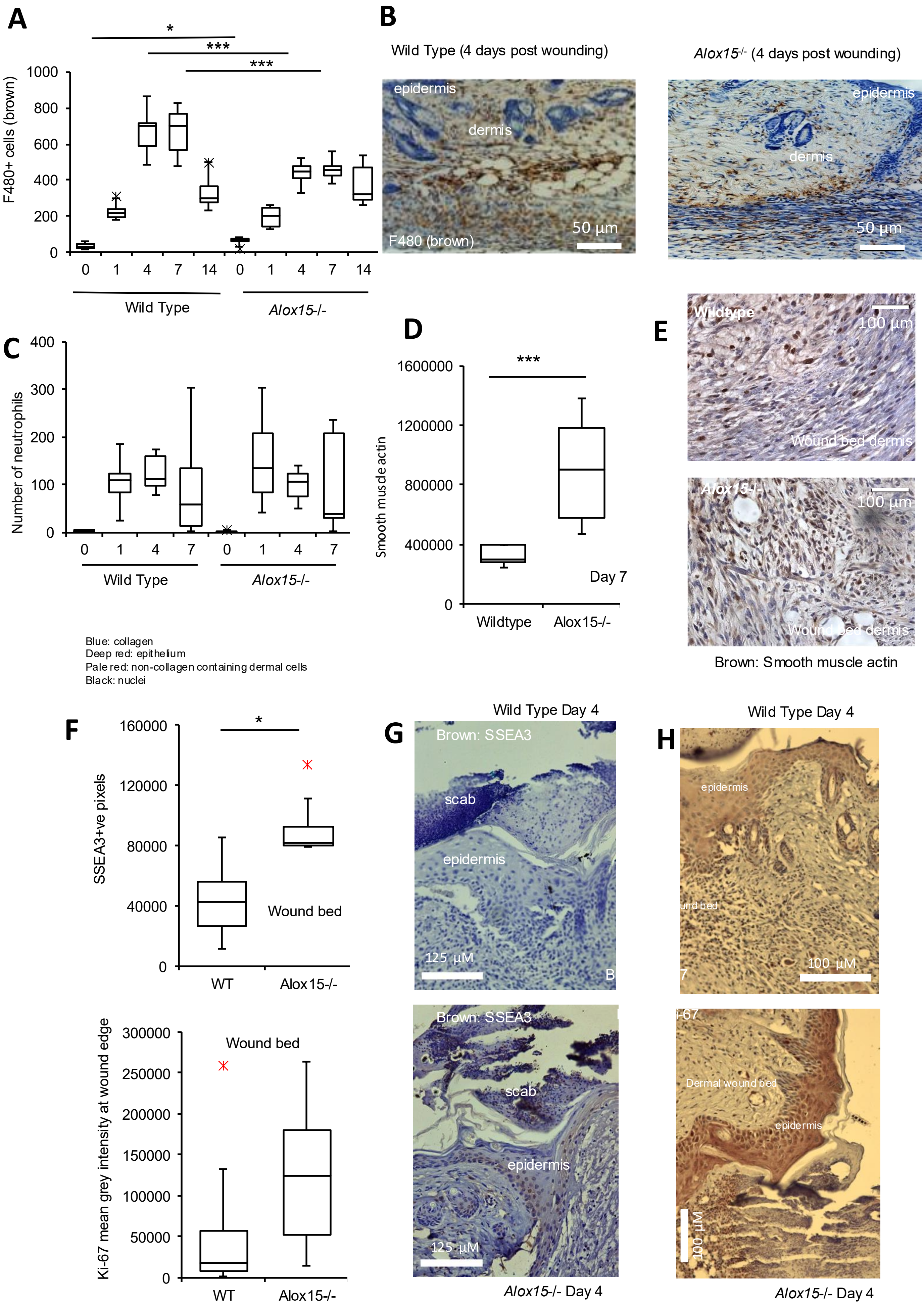

Supplementary Figure 3

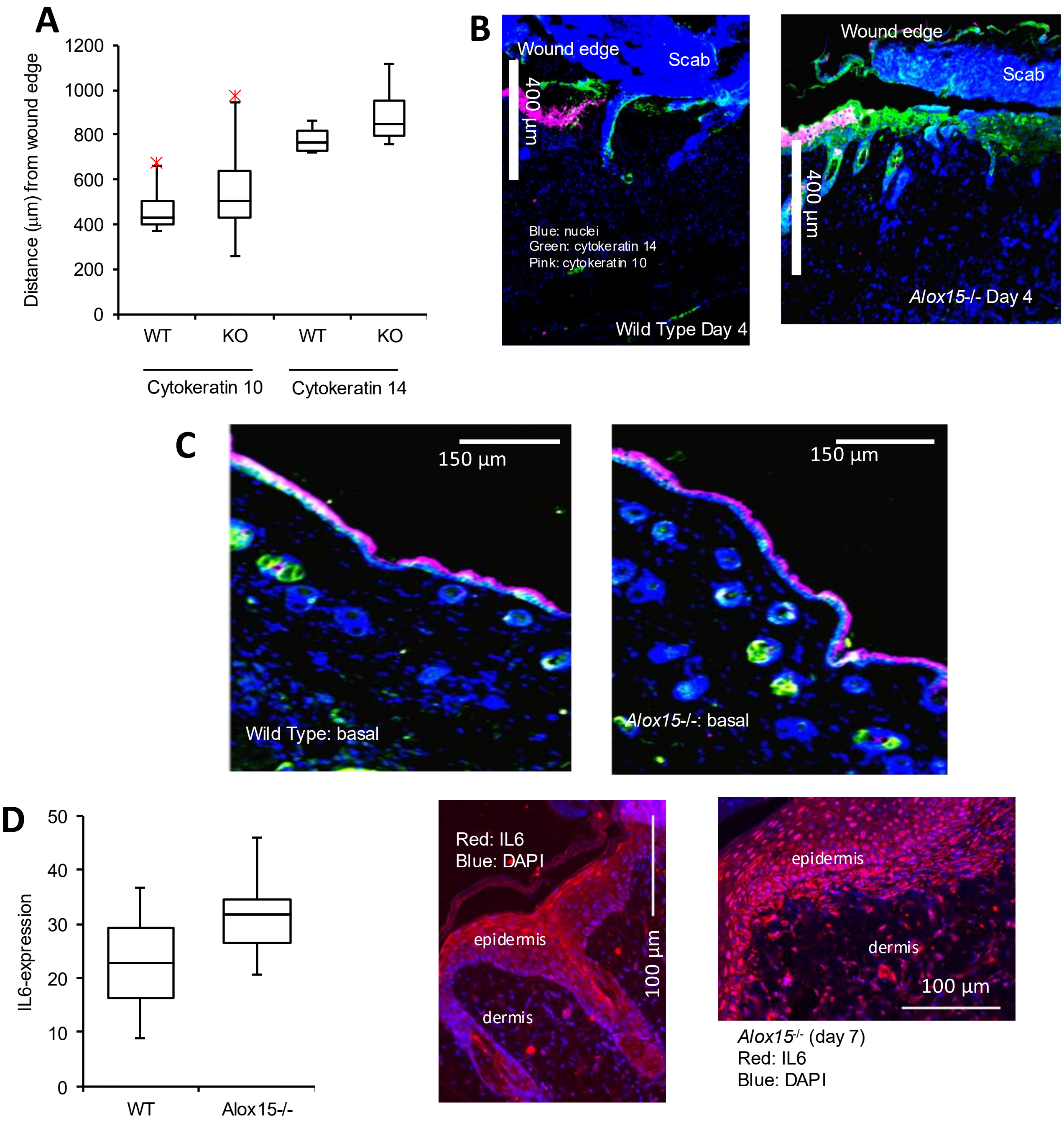

Supplementary Figure 4

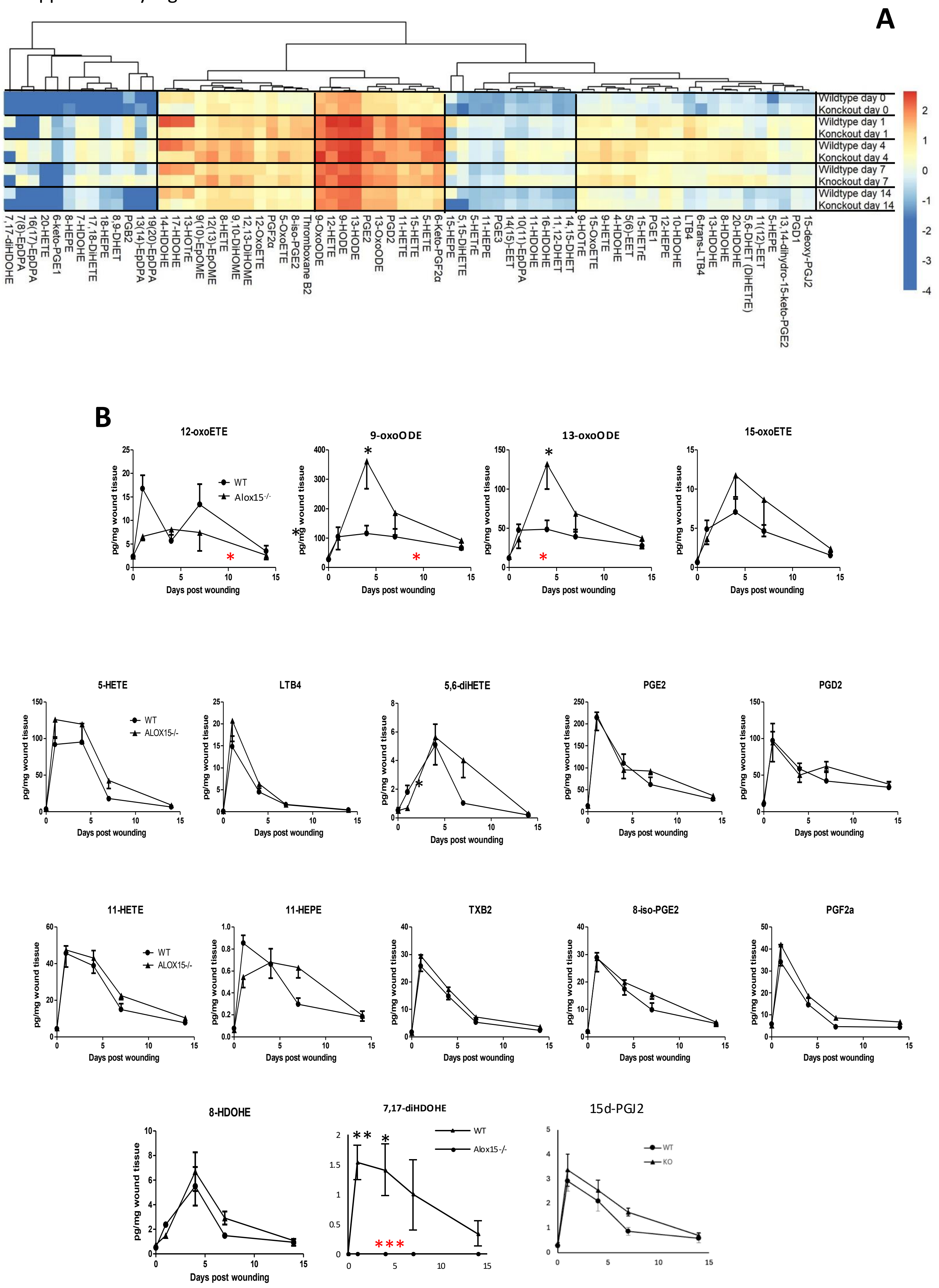

Supplementary Figure 5

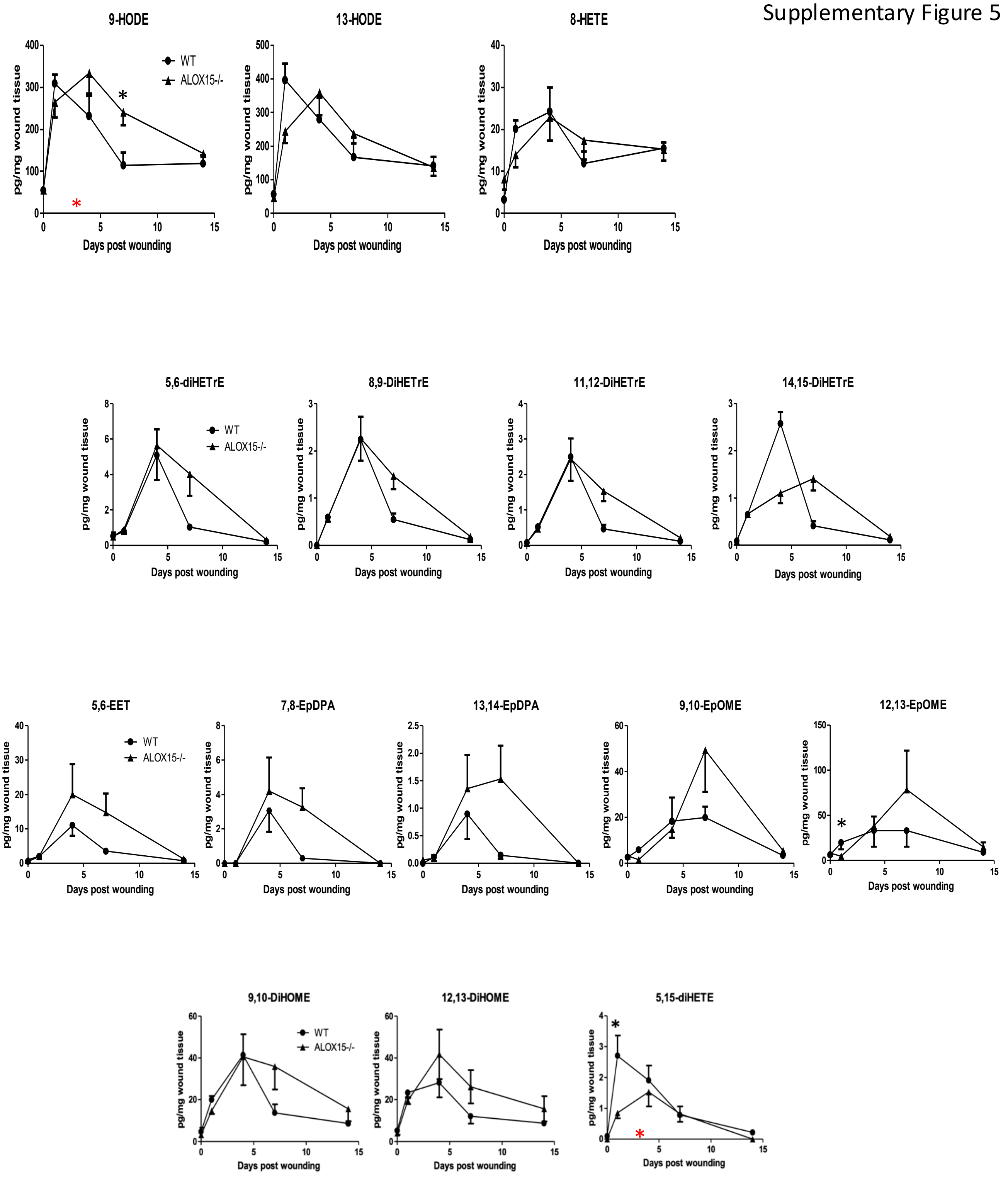

A

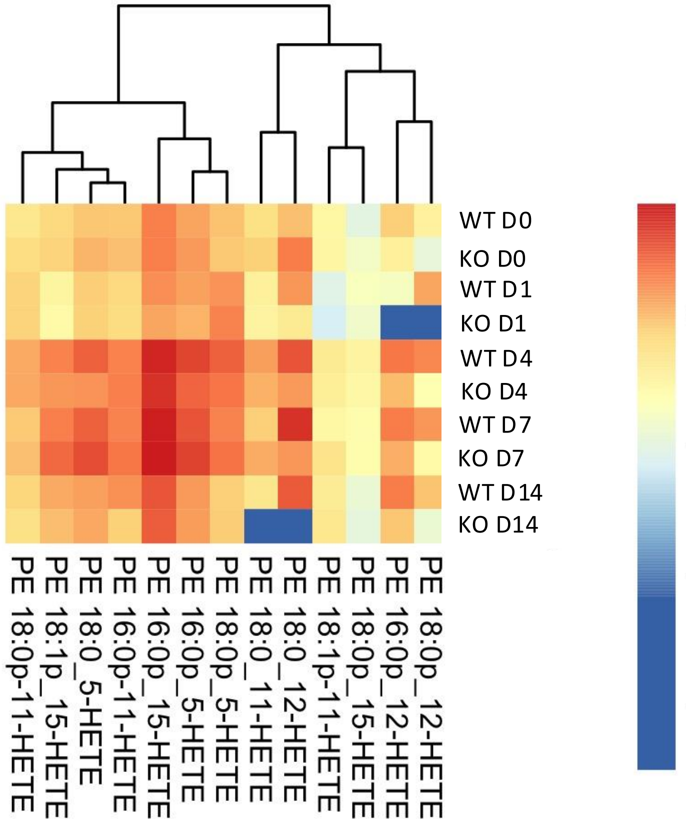
WT D0
KO D0
WT D1
KO D1
WT D4
KO D4
WT D7
KO D7
WT D14
KO D14
PE 18:0p-11-HETE
PE 18:1p_15-HETE
PE 18:0_5-HETE
PE 16:0p-11-HETE
PE 16:0p_15-HETE
PE 16:0p_5-HETE
PE 18:0p_5-HETE
PE 18:0_11-HETE
PE 18:0_12-HETE
PE 18:1p-11-HETE
PE 18:0p_15-HETE
PE 16:0p_12-HETE
PE 18:0p_12-HETE
0
-1
-2
-3

B

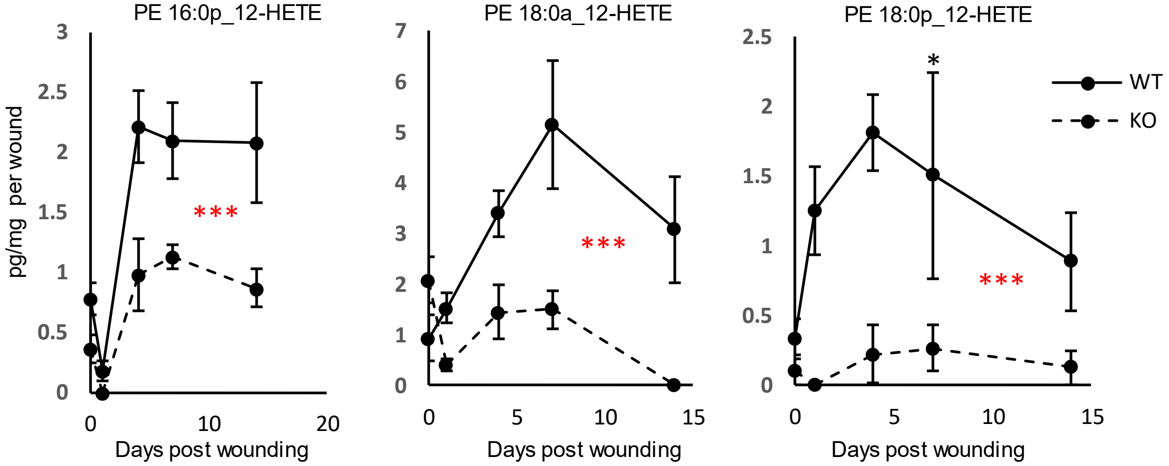
PE 16:0p_12-HETE
PE 18:0a_12-HETE
PE 18:0p_12-HETE
pg/mg per wound
Days post wounding
***
*
WT
KO

Supplementary Figure 7

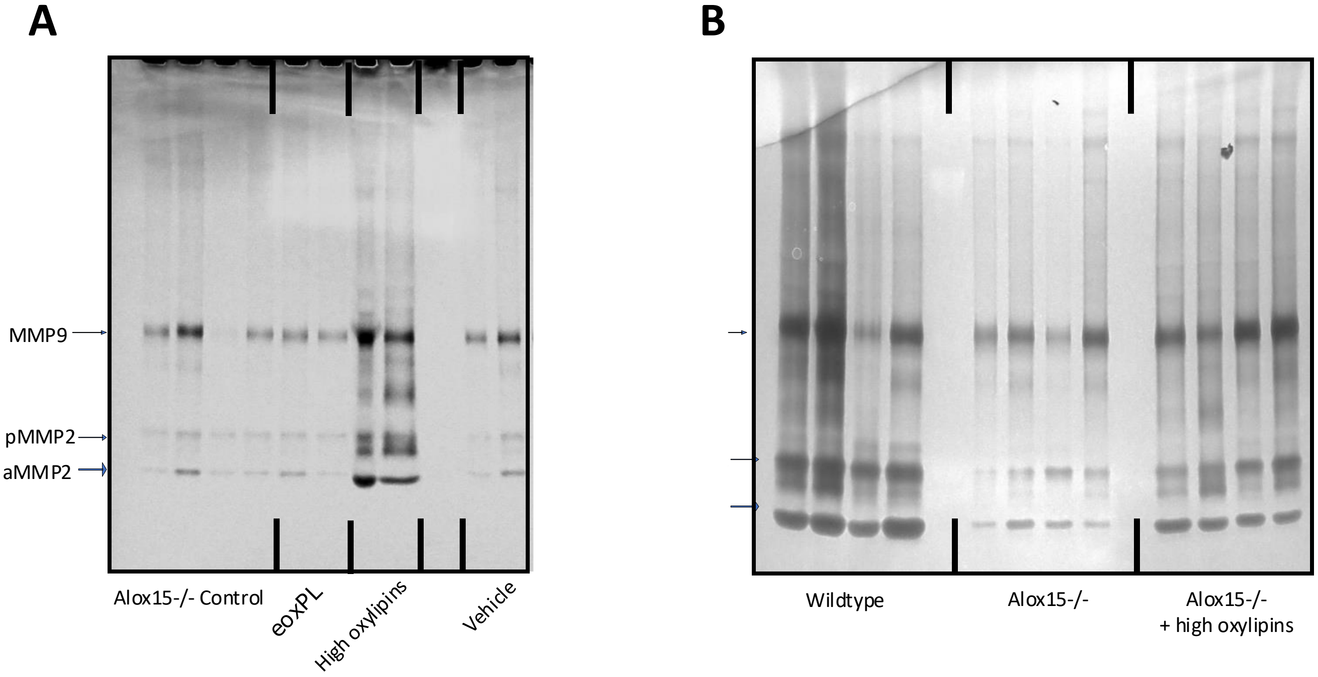

Supplementary Figure 8

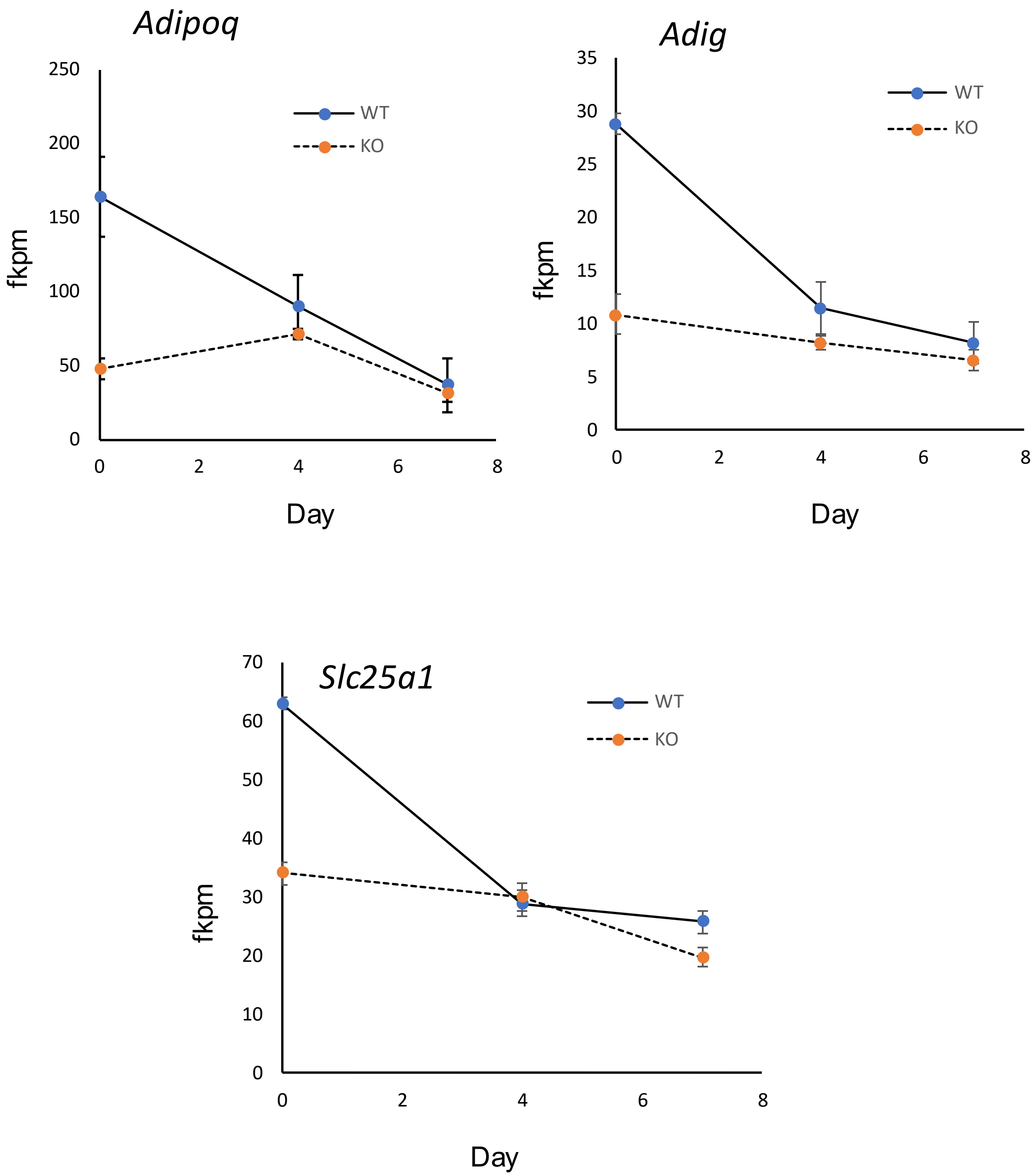

Supplementary Figure 9

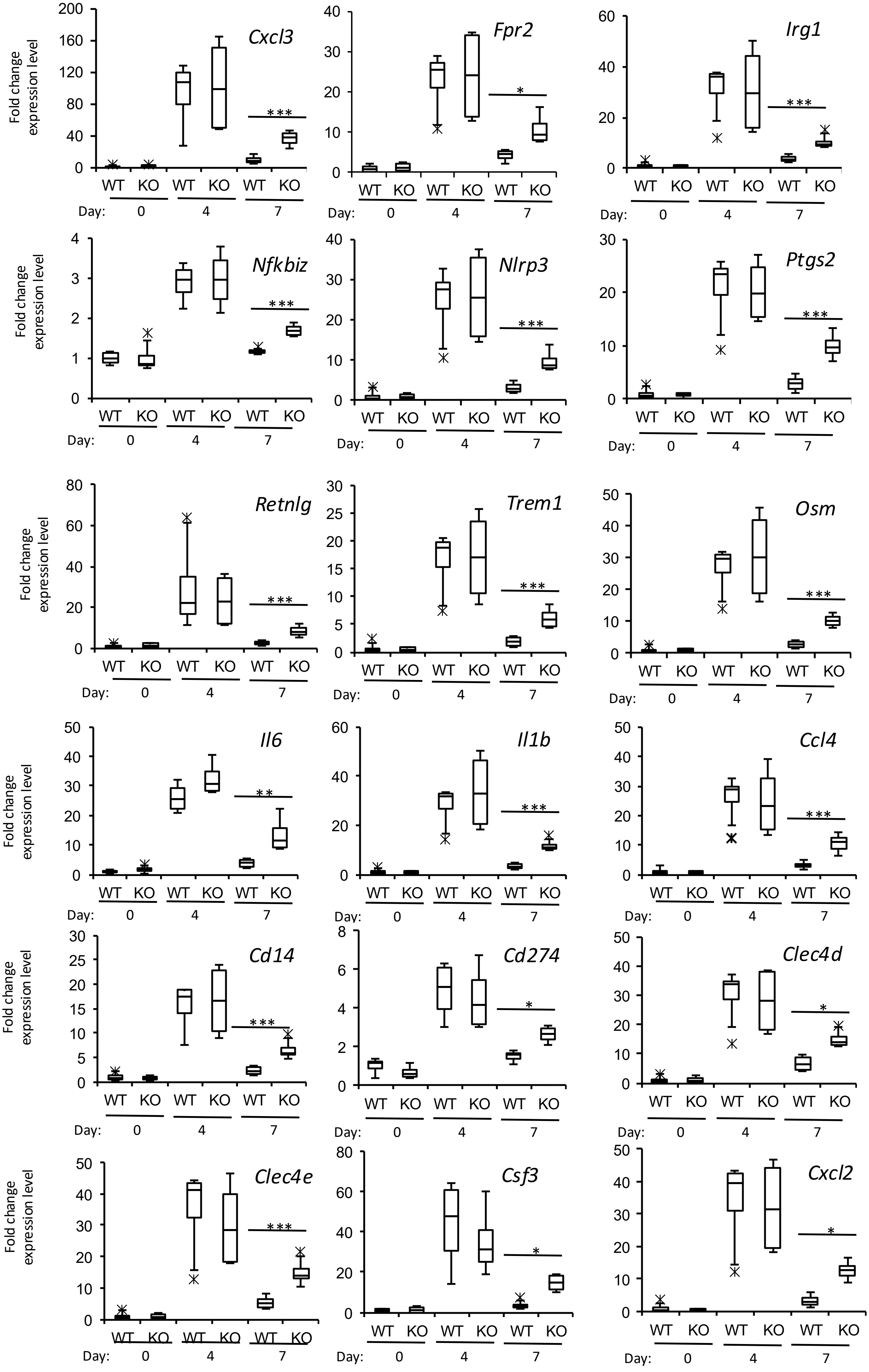

Supplementary Figure 10

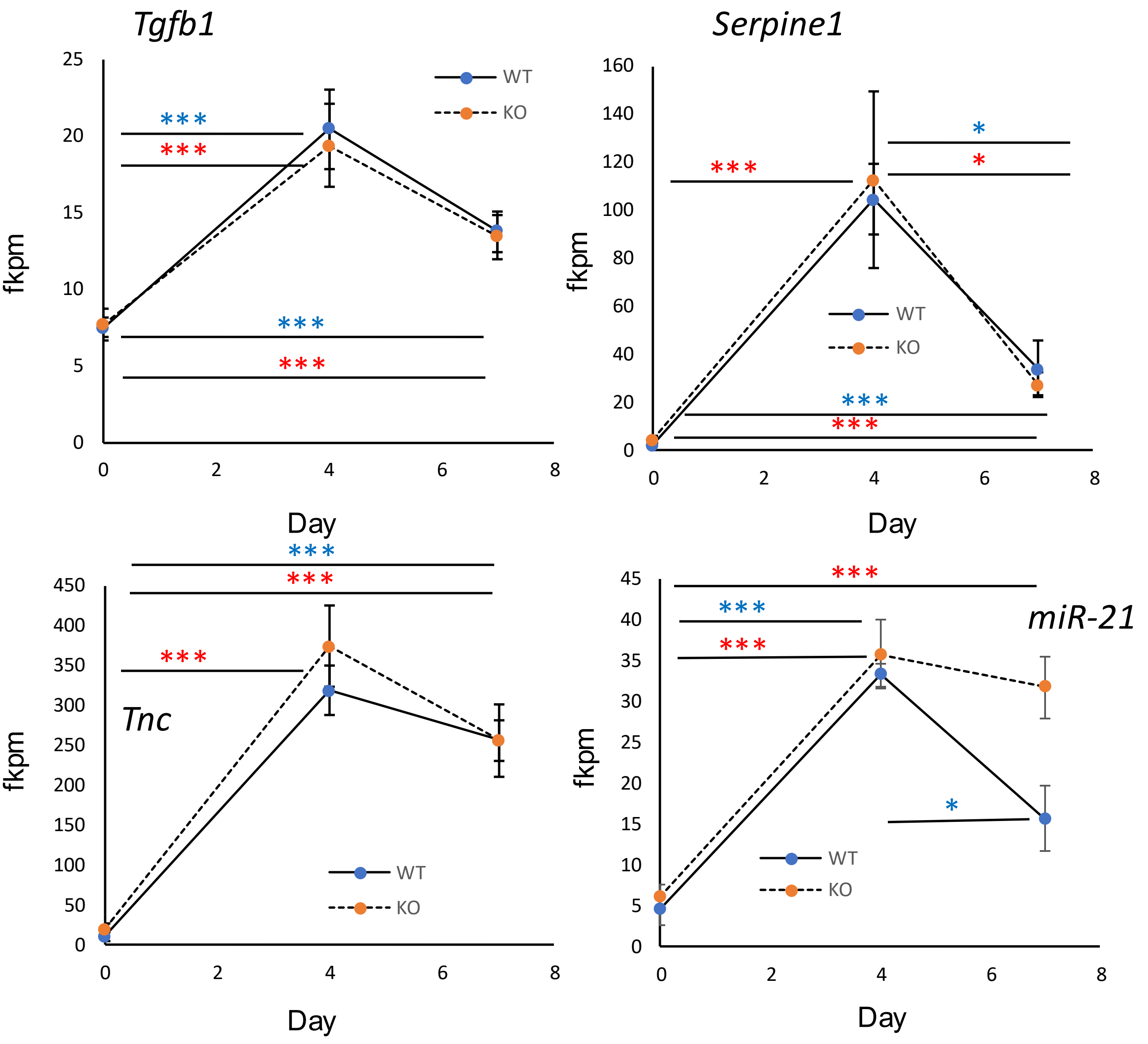

Supplementary Figure 11

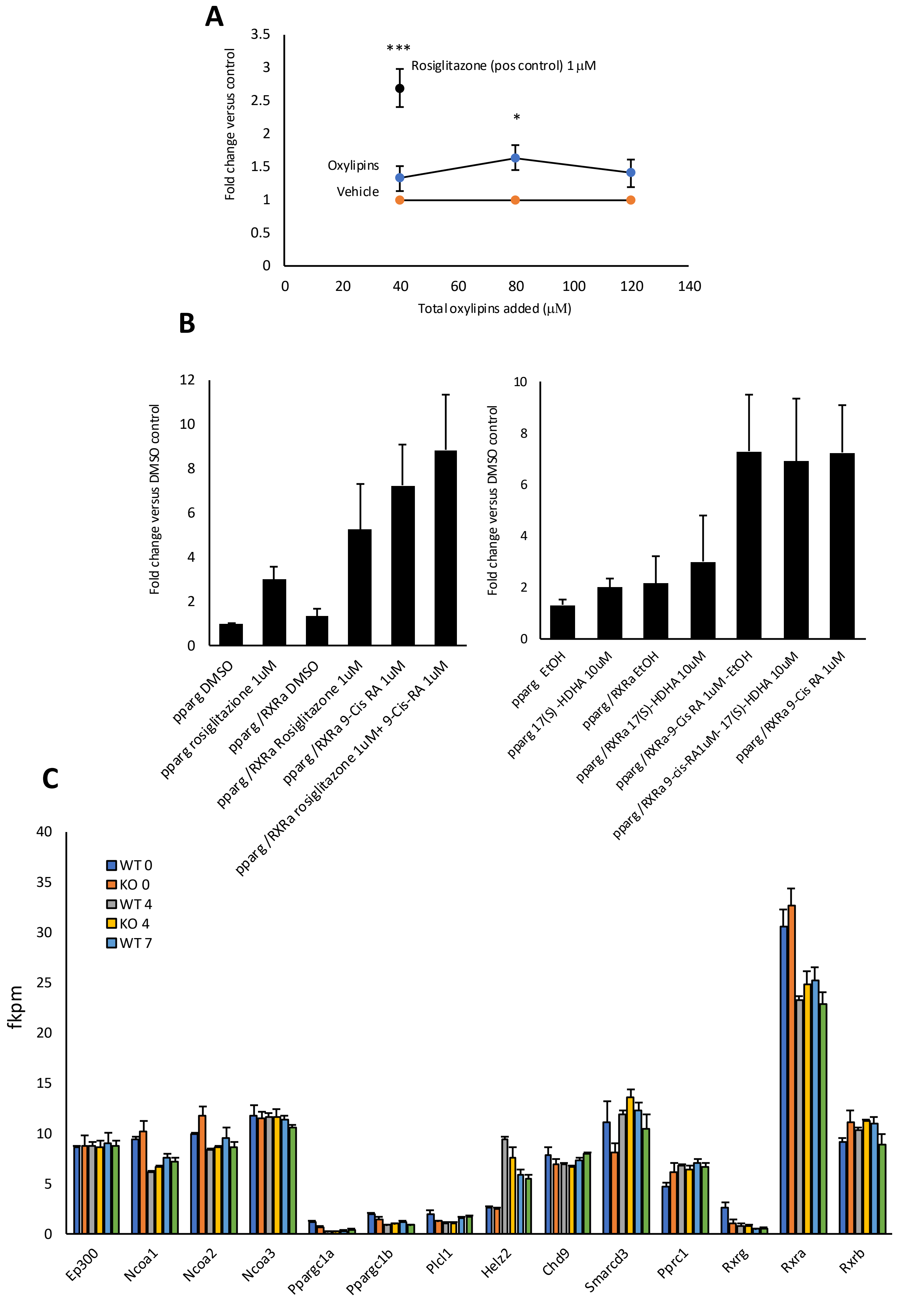

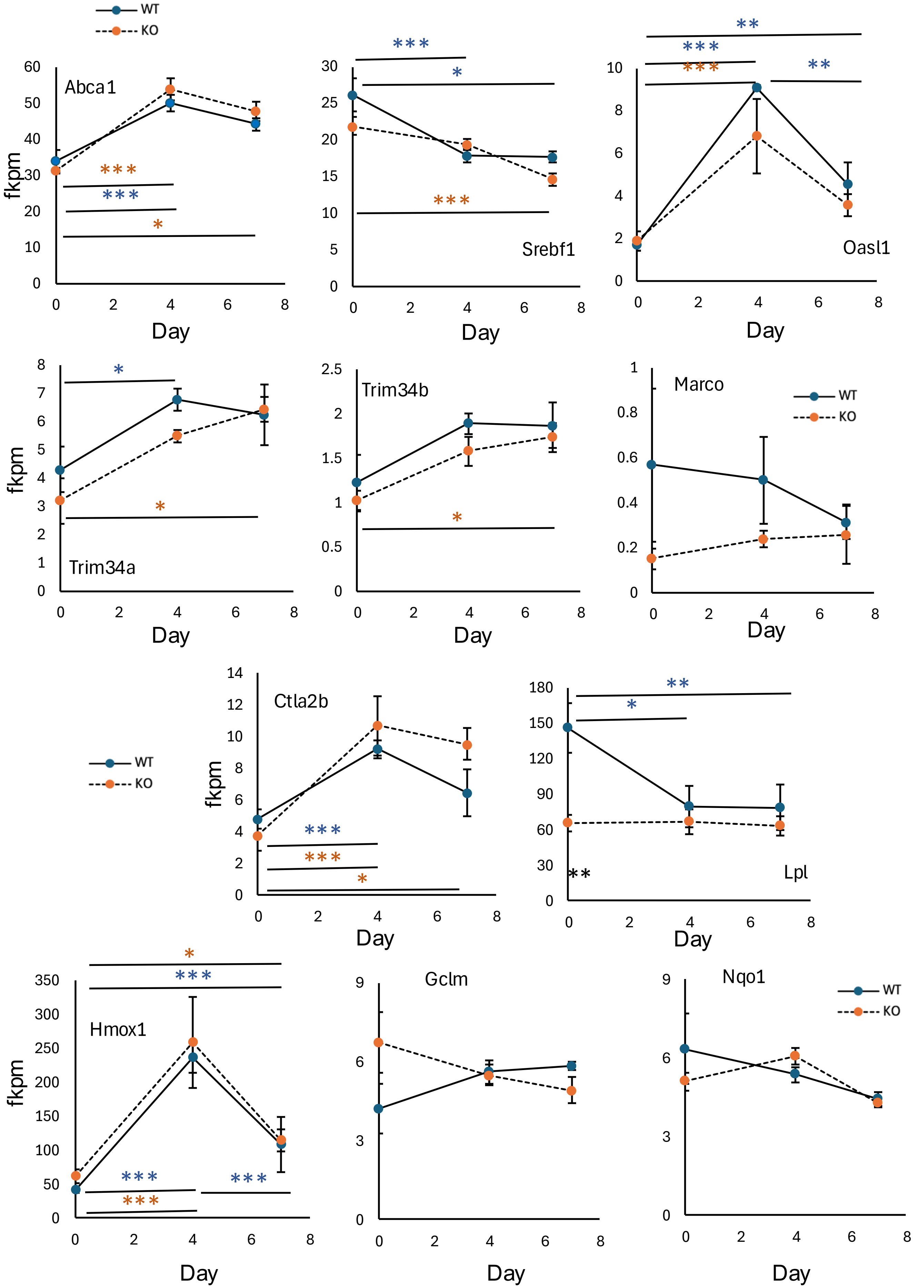
WT
KO
Abca1
Srebf1
Oasl1
Trim34a
Trim34b
Marco
Ctla2b
Lpl
Hmox1
Gclm
Nqo1
fkpm
Day

Supplementary Figure 13

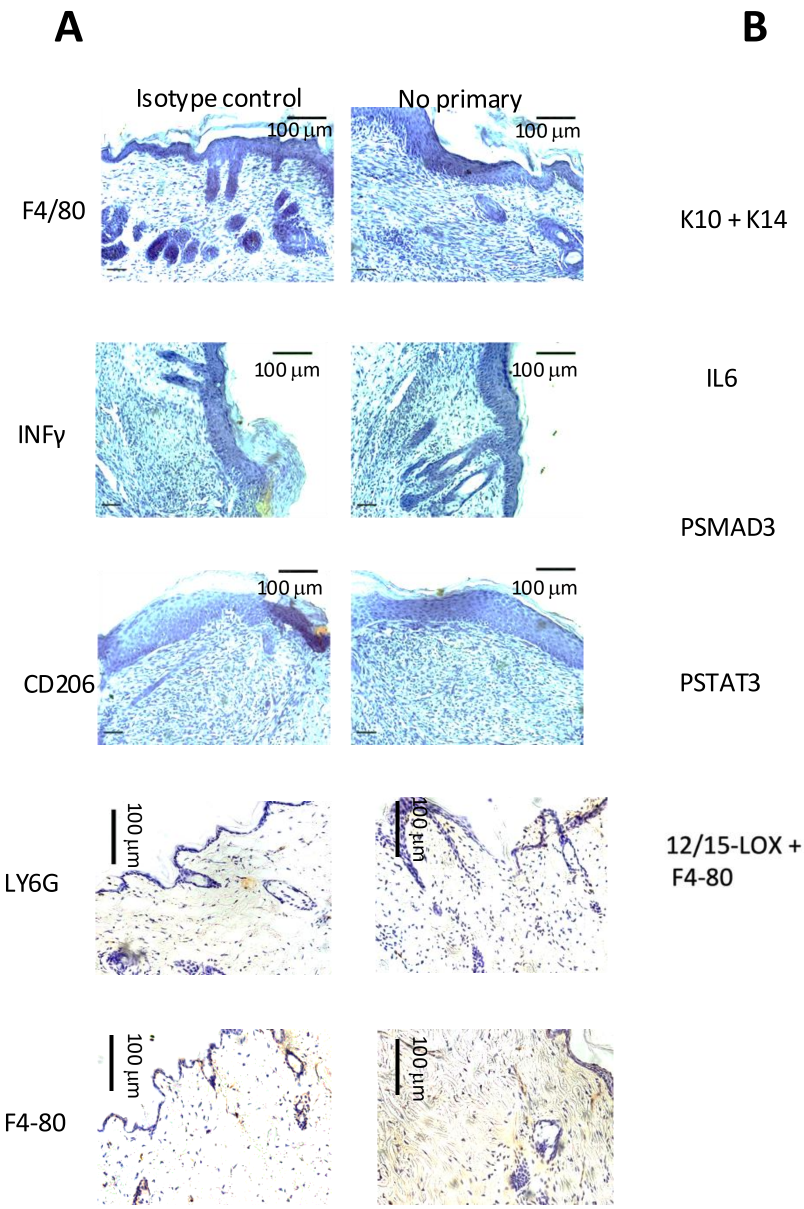

Supplementary Figure 14

5-HETE

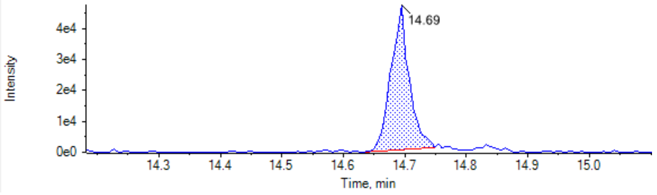

8-HETE

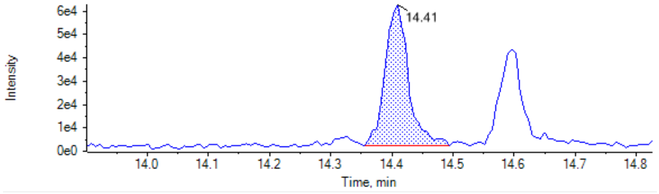

9-HETE

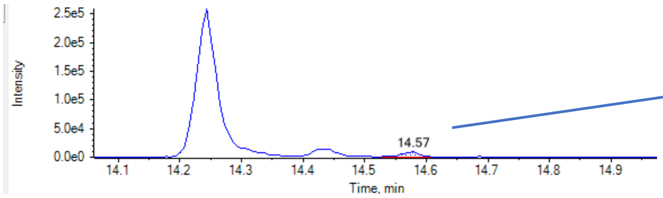

9-HETE (zoom)

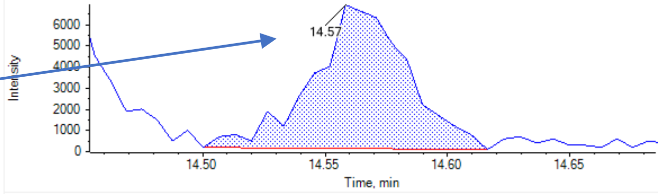

11-HETE

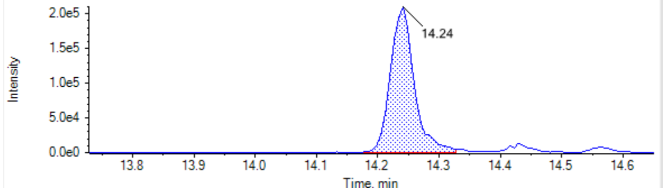

12-HETE

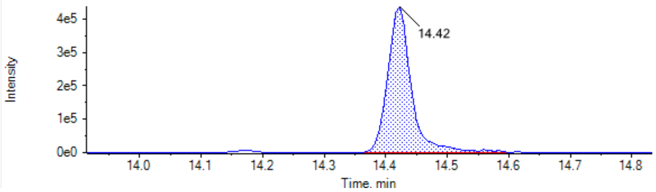

15-HETE

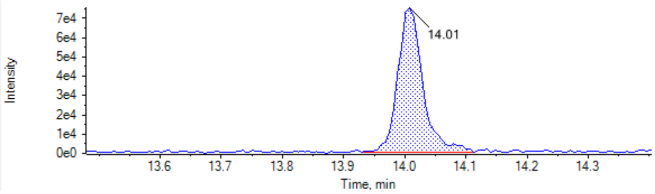

20-HETE

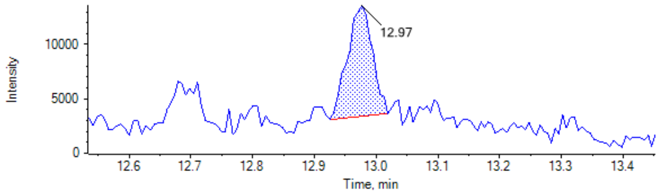

5-HEPE

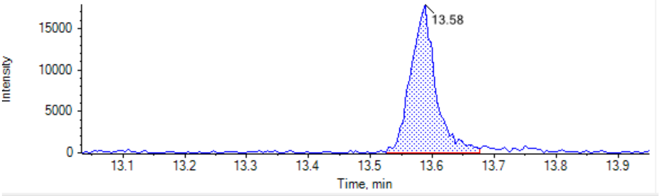

8-HEPE

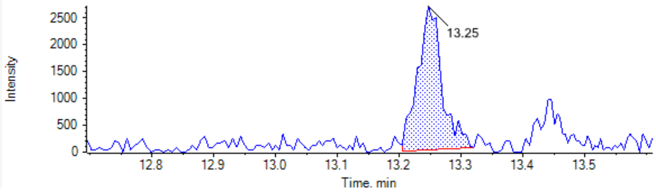

11-HEPE

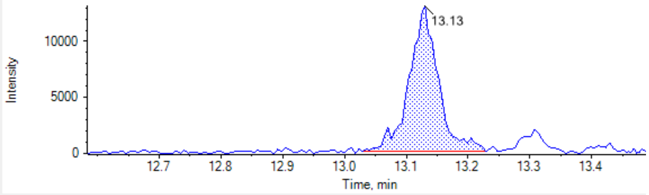

Supplementary Figure 14 (cont'd)

12-HEPE

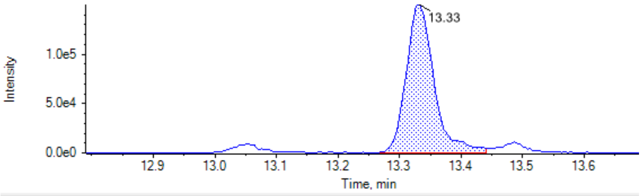

15-HEPE

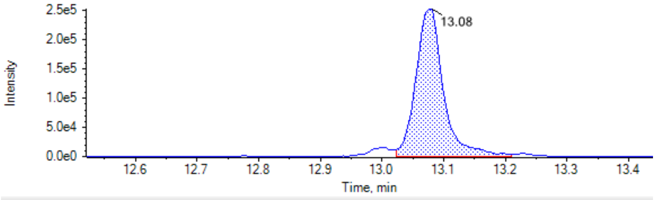

18-HEPE

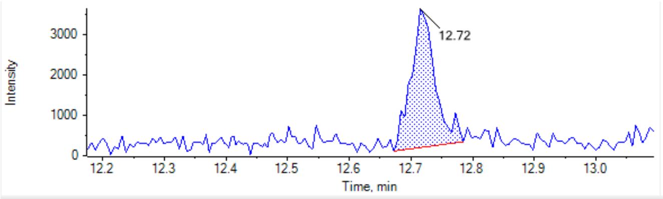

4-HDOHE

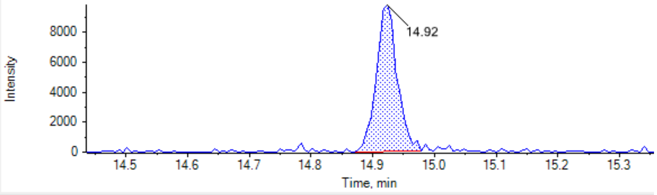

7-HDOHE

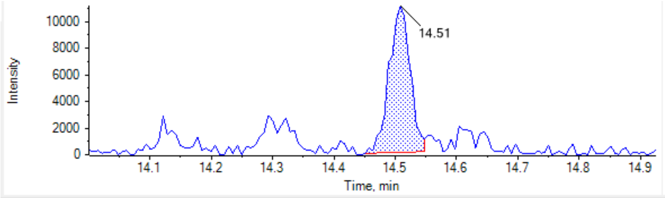

8-HDOHE

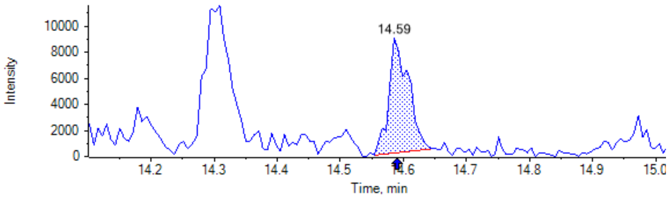

20-HDOHE

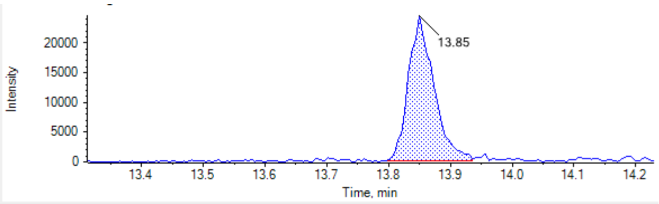

9-HODE

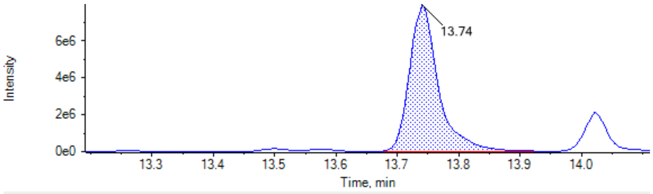

13-HODE

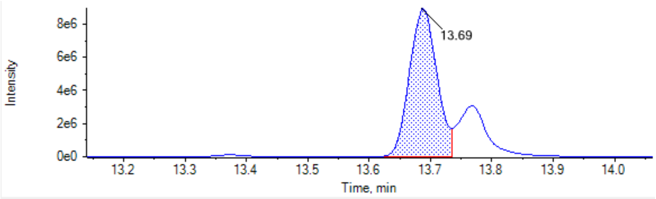

9-HOTrE

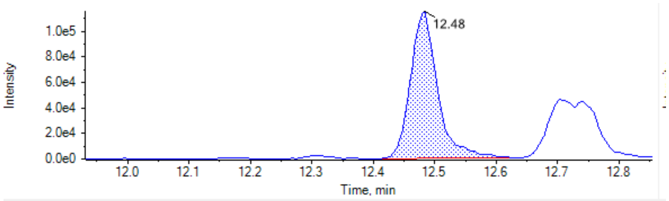

13-HOTrE

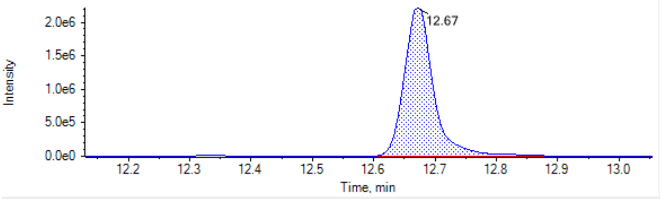

5-HETrE

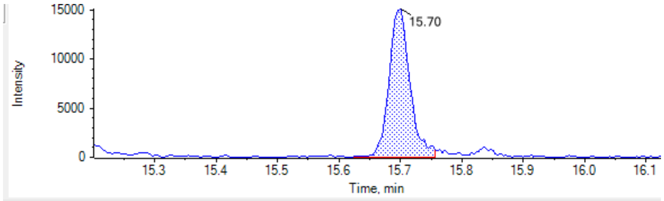

Supplementary Figure 14 (cont'd)

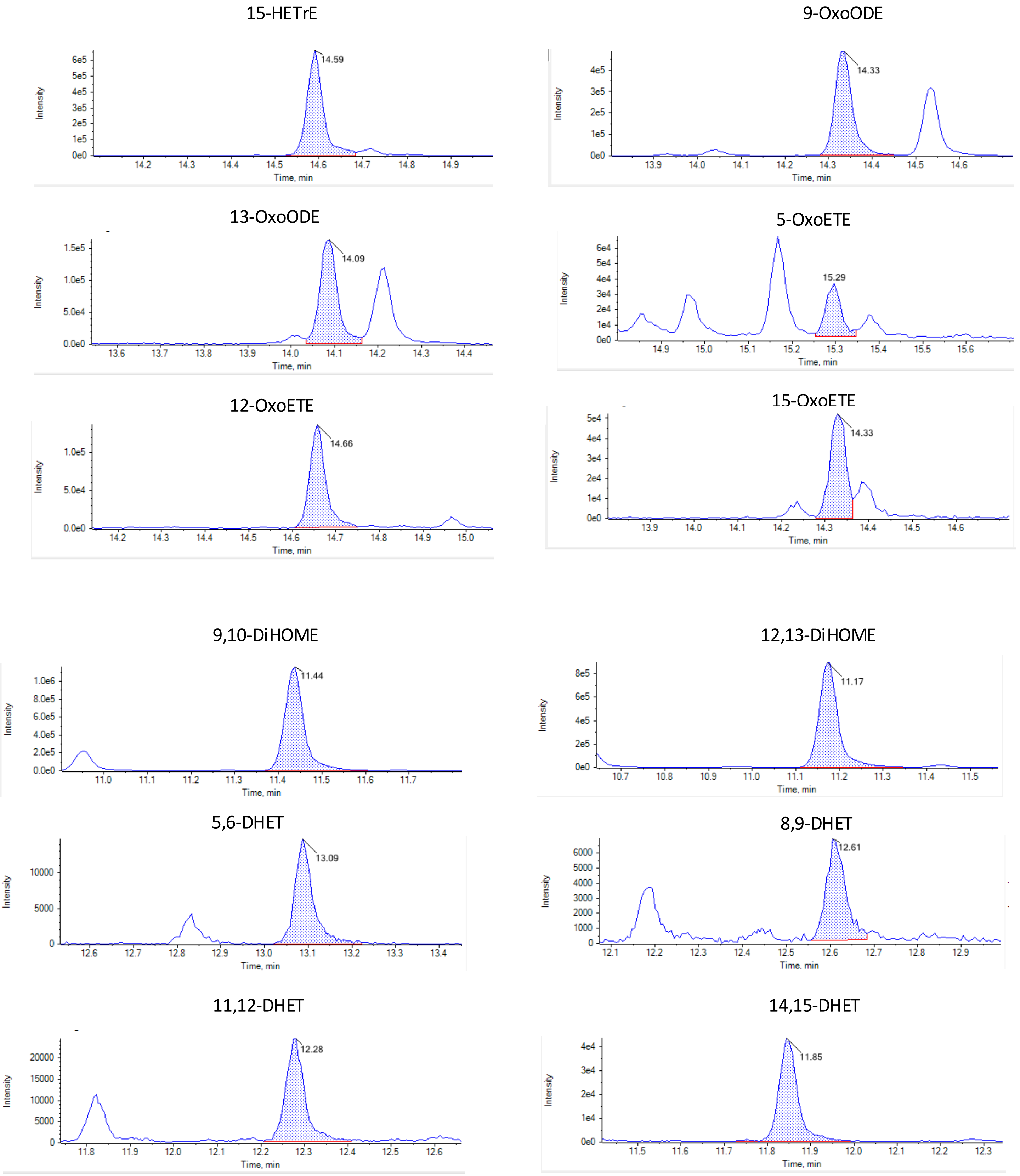

Supplementary Figure 14 (cont'd)

5,15-DiHETE

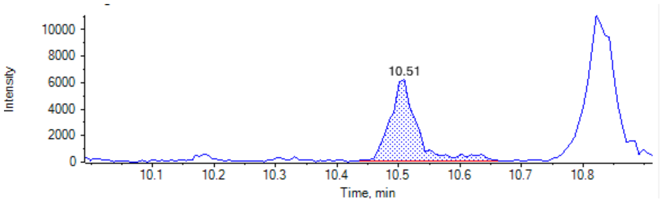

17,18-DiHETE

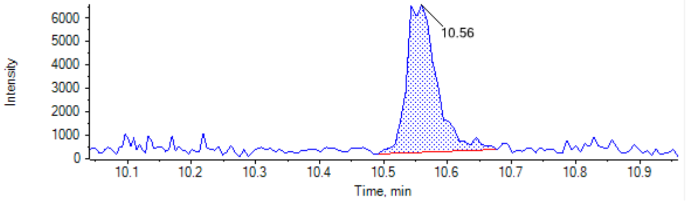

7,17-diHDOHE, co-elutes with resolvinD5 standard

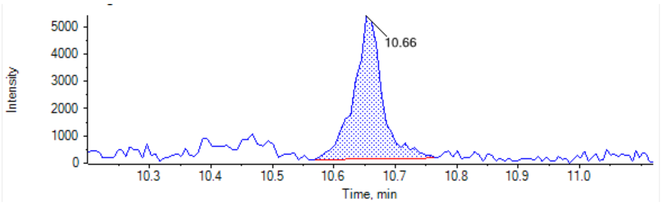

Leukotriene B4

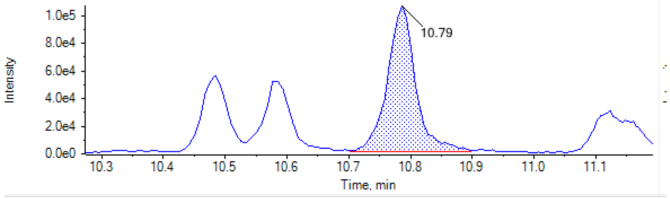

9,10-EpOME

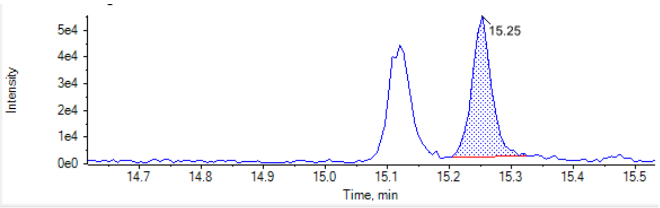

12,13-EpOME

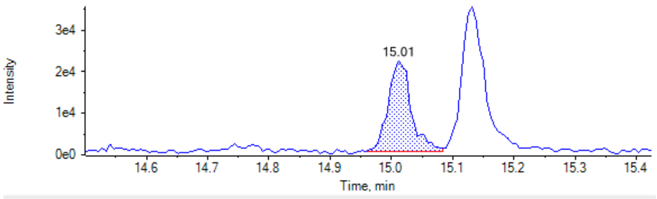

5,6-EET

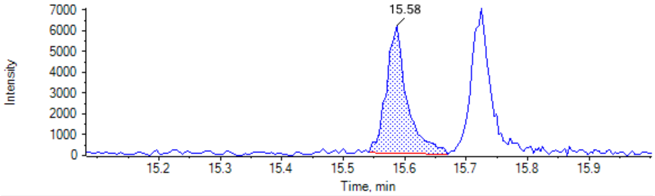

Supplementary Figure 14 (cont'd)

11,12-EET

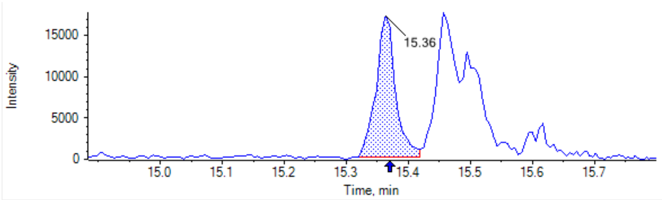

14,15-EET

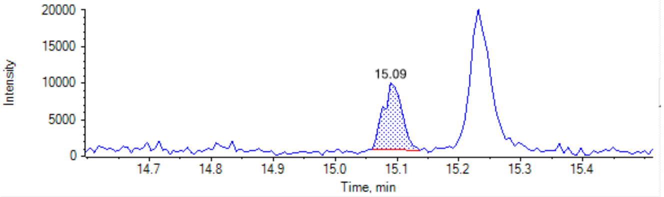

7,8-EpDPA

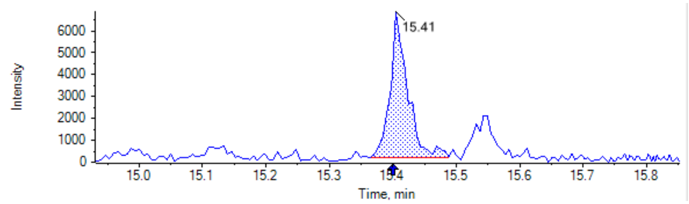

10,11-EpDPA

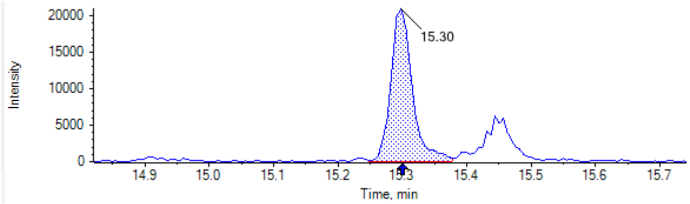

13,14-EpDPA

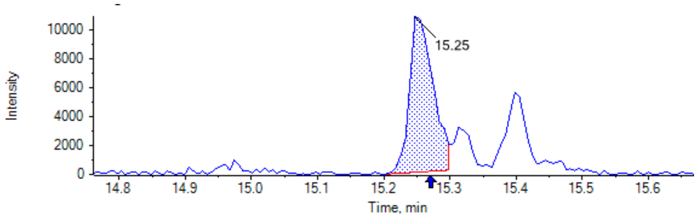

16,17-EpDPA

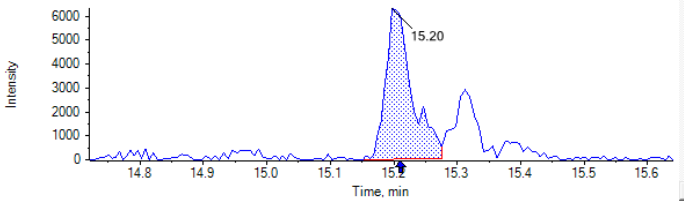

PGD2

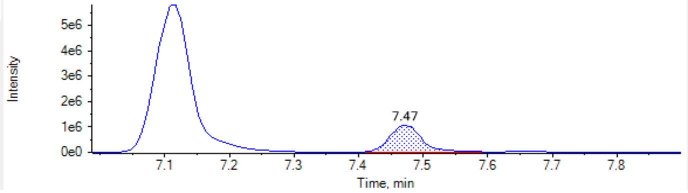

PGE1

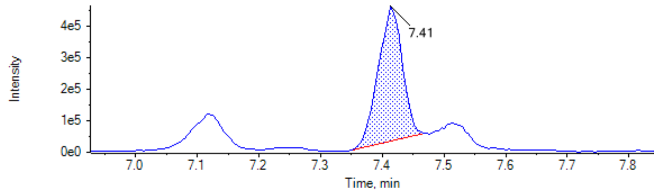

PGD1

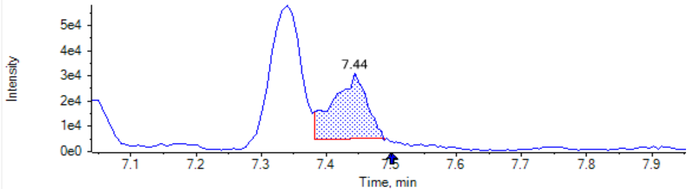

Supplementary Figure 14 (cont'd)

PGE2

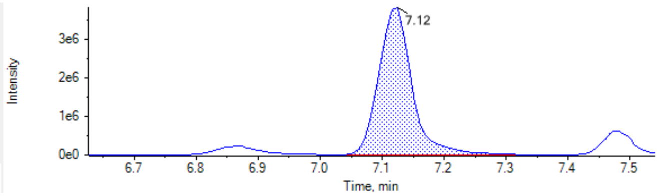

PGE3

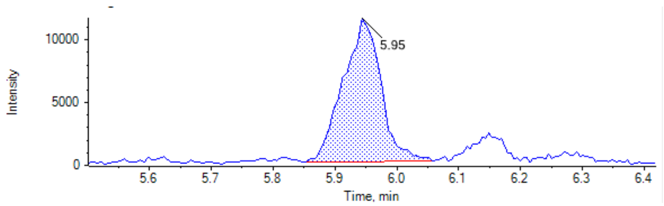

PGB2

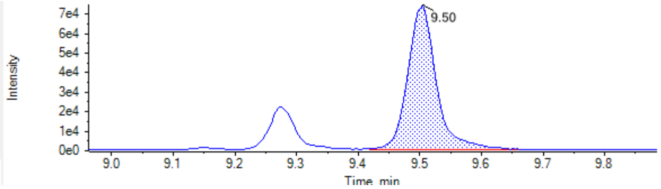

13,14-dihydro-15-keto PGE2

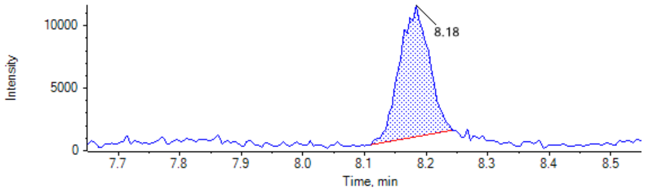

15-deoxy-PGJ2

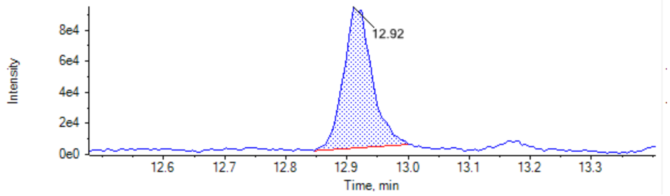

PGF2α

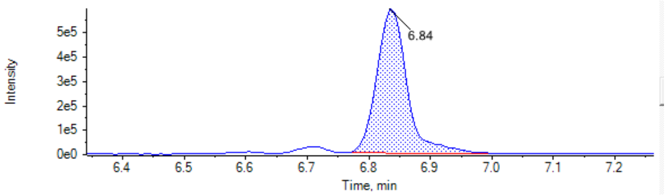

Thromboxane B2

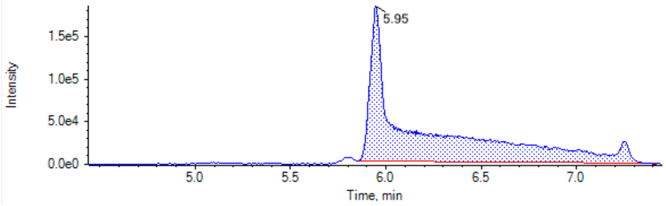

8-iso PGE2

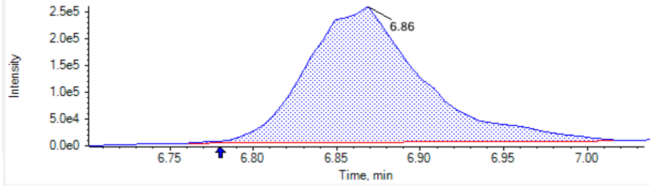

6-trans Leukotriene B4

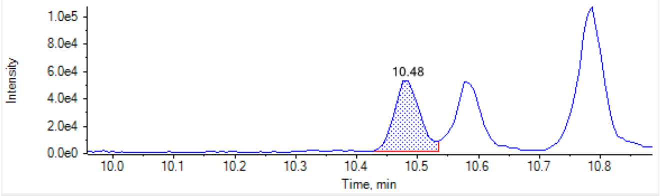

Supplementary Figure 15

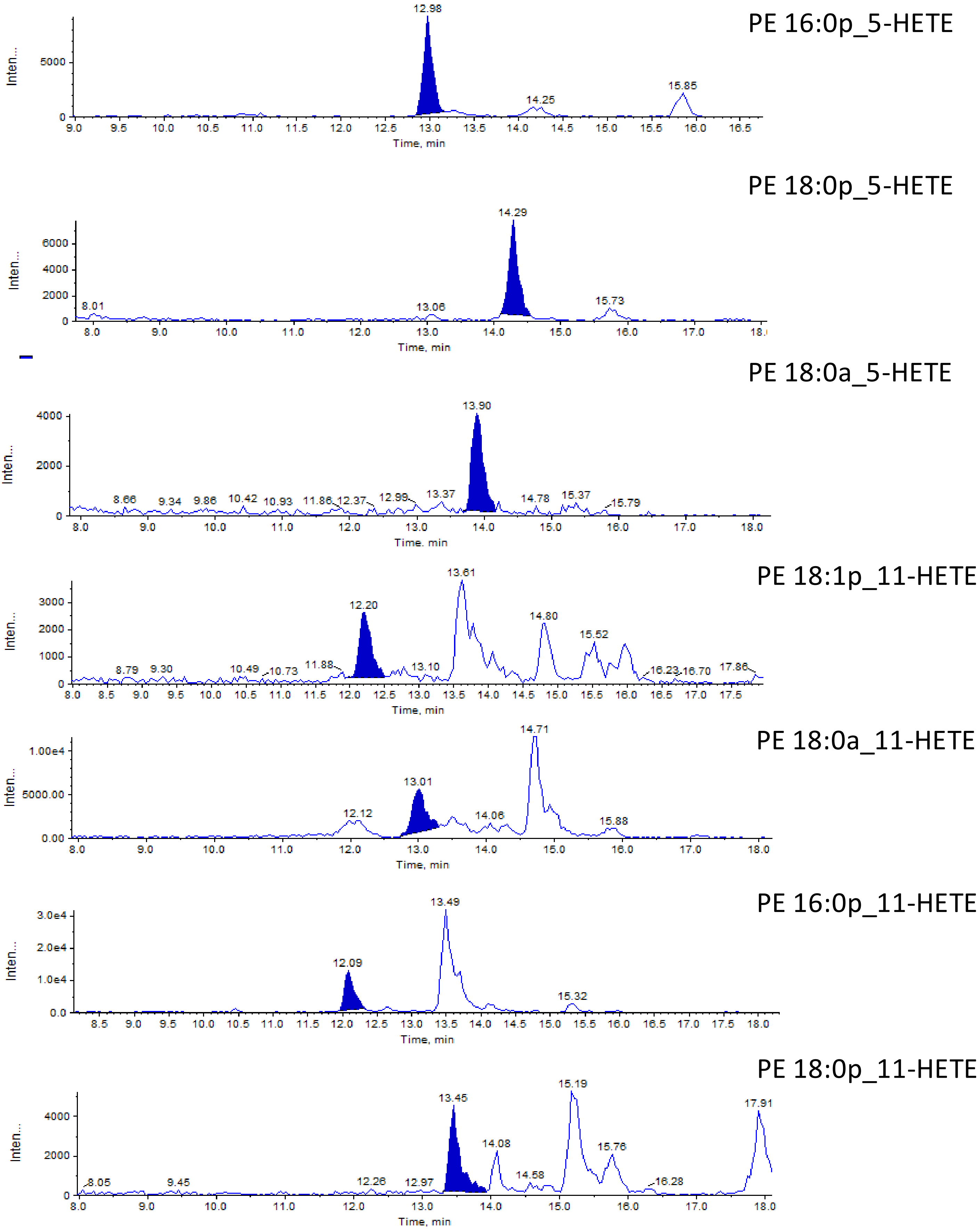

Supplementary Figure 15 (cont'd)

PE 16:0p_12-HETE

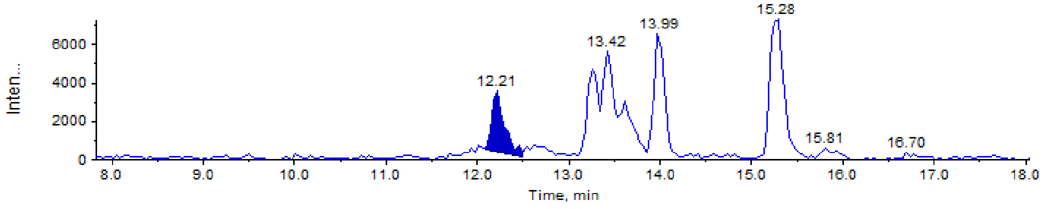

PE 18:0p_12-HETE

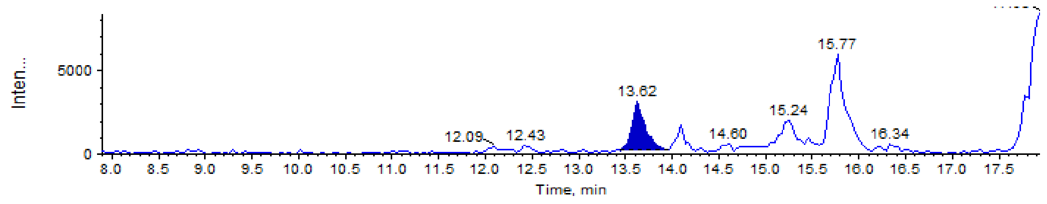

PE 18:0a_12-HETE

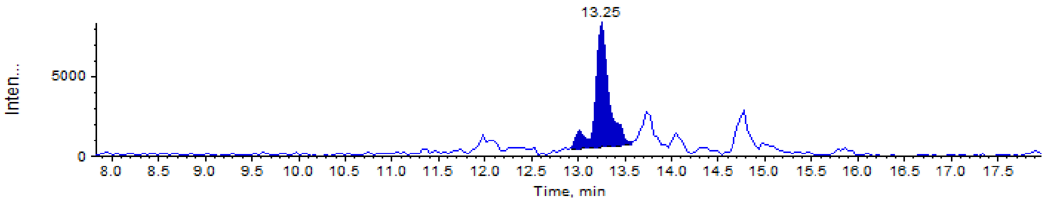

PE 16:0p_15-HETE

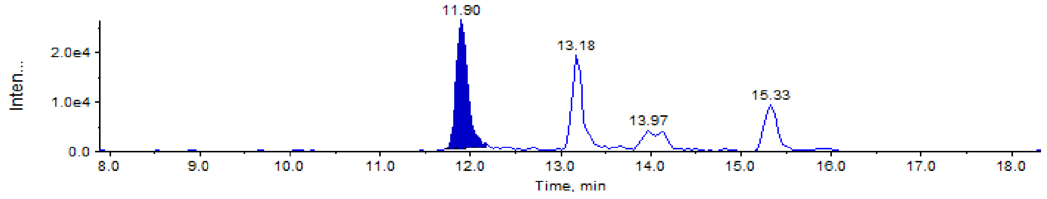

PE 18:1p_15-HETE

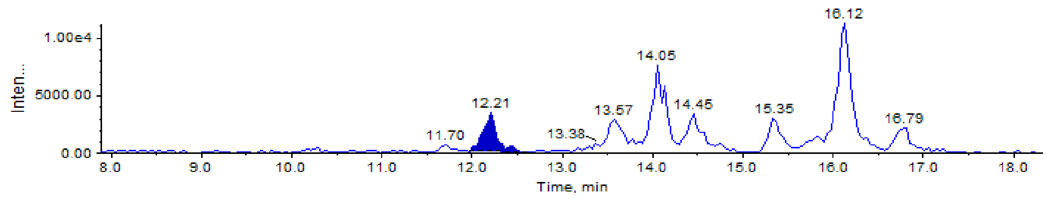

PE 18:0p_15-HETE

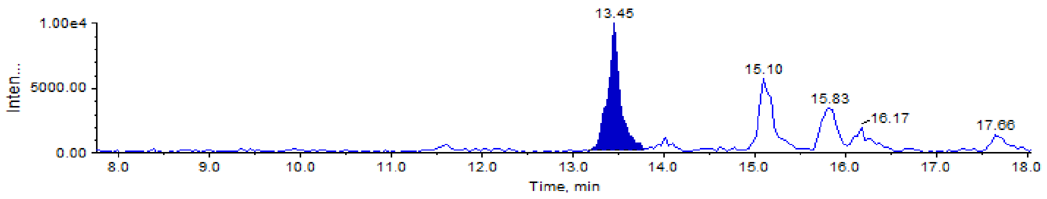

PE 18:0a_15-HETE

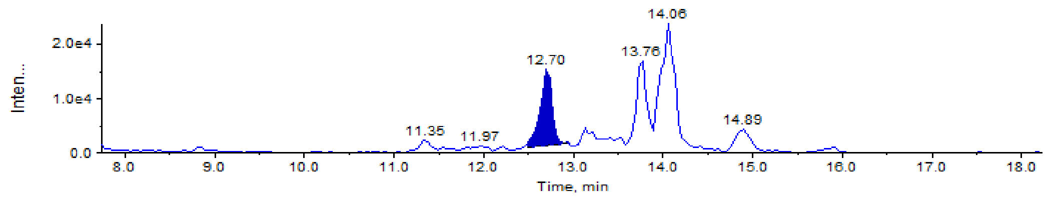

Supplementary Figure 16

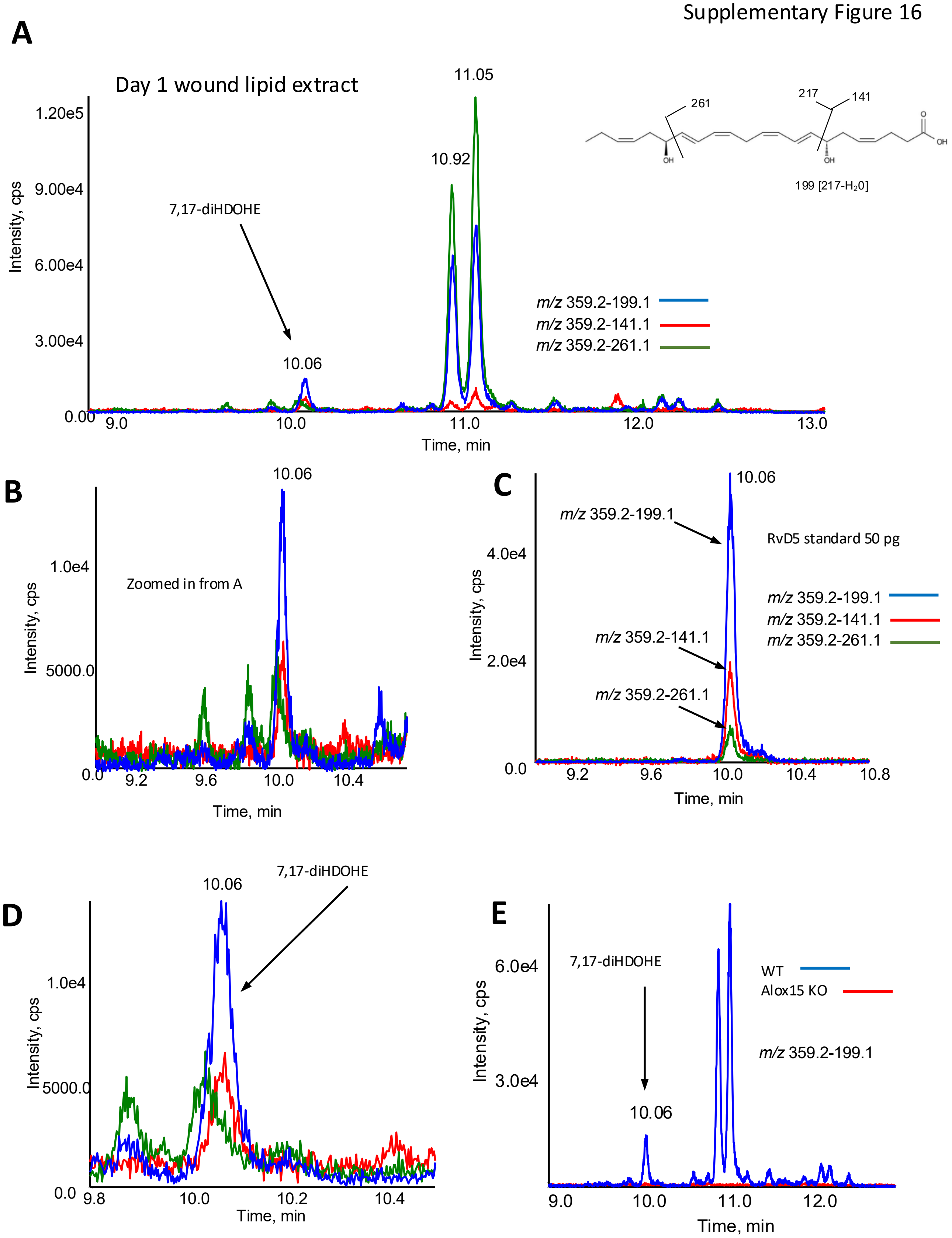

Further zoomed in showing that /z 359.2-261.1 doesn't co-elute with the other MRM transitions.

Several peaks present in WT extract are absent in *Alox15*$^{-/-}$, including 7,17-diHDOHE (RvD5).

Supplementary Figure 16 (cont'd)

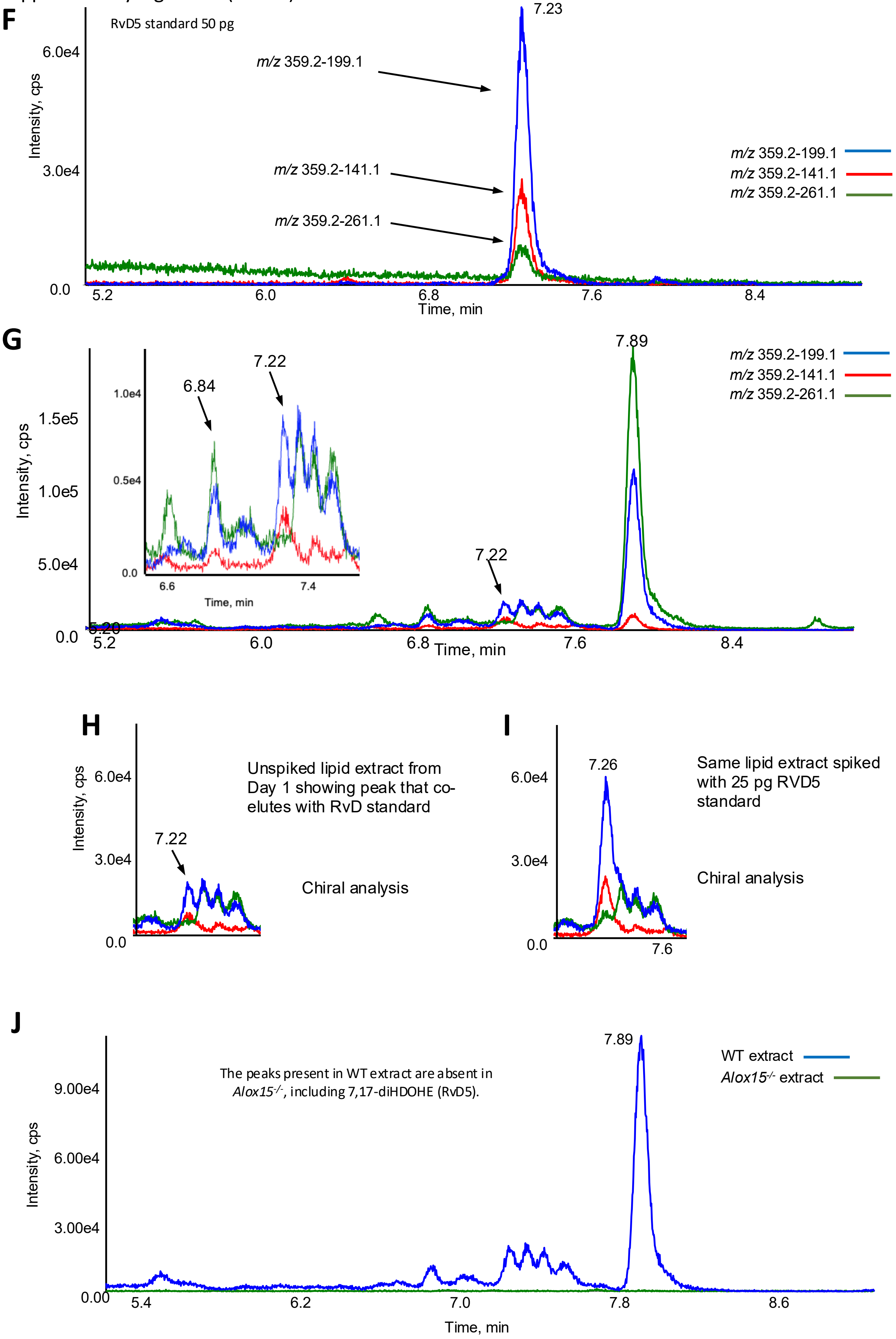

Supplementary Figure 17

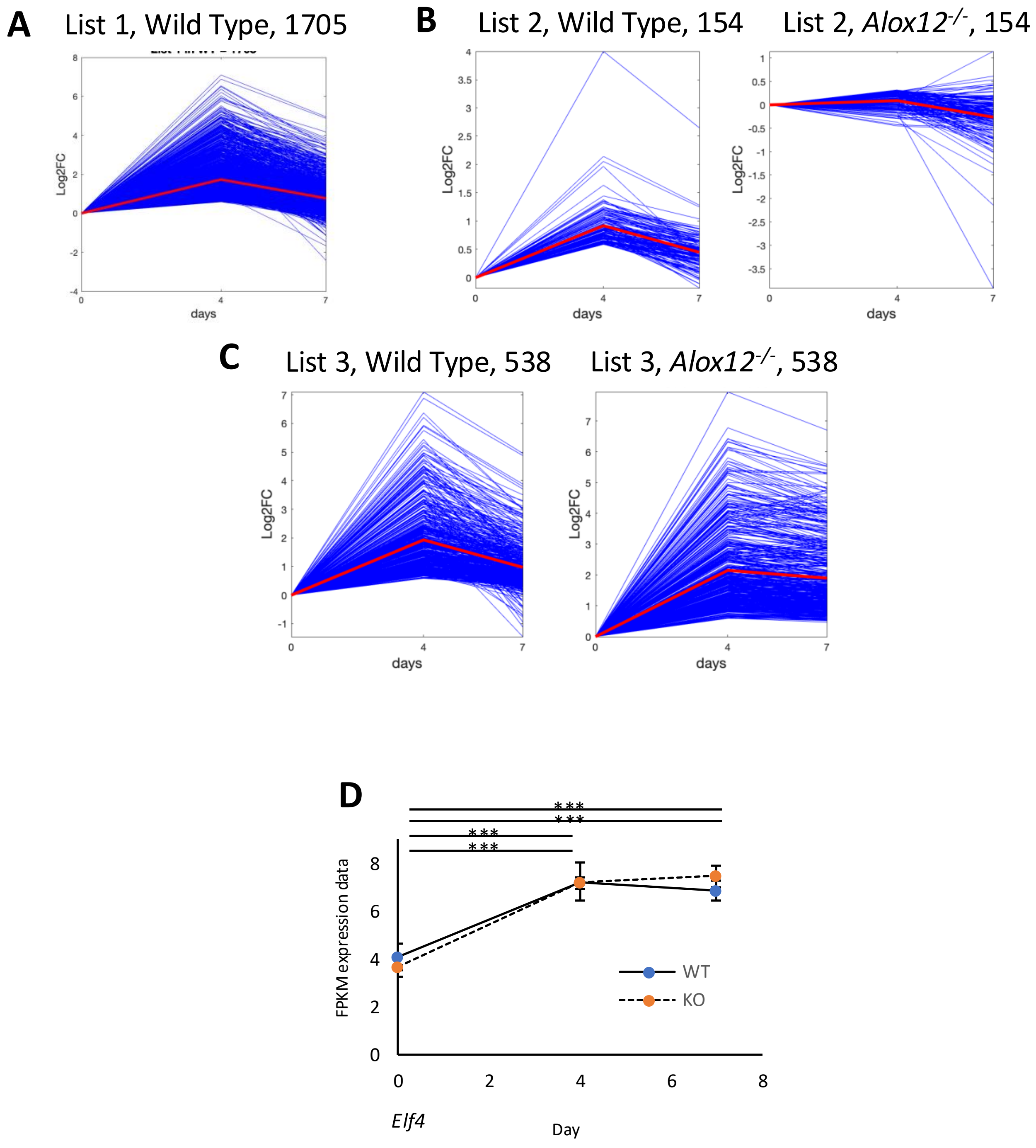

Supplementary Figure 18

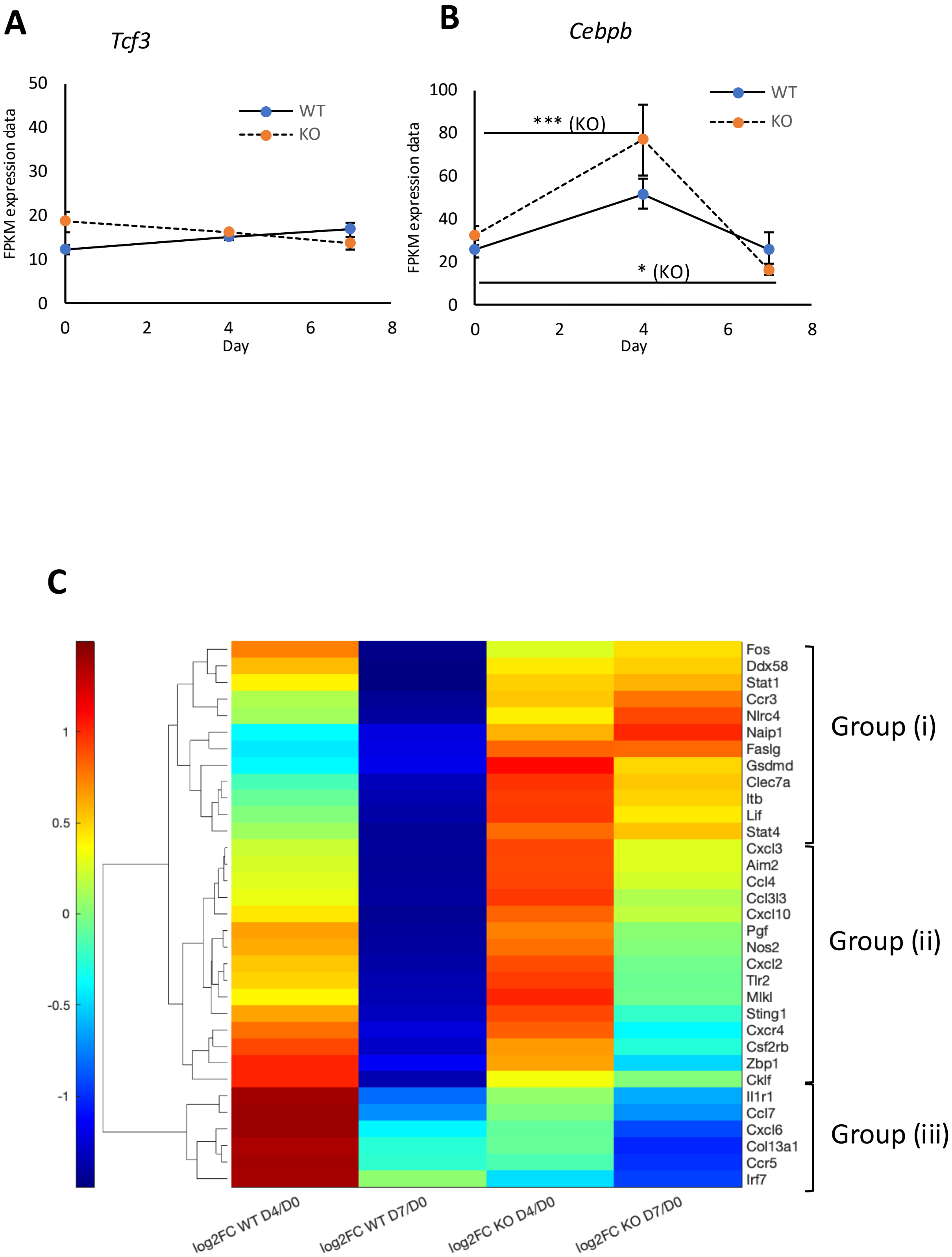

Supplement: Supplementary file 1 — Appendix 01 (PDF) [file pnas.2502640122.sapp.pdf]
